# Supplementary material for: Rivaroxaban, a direct inhibitor of coagulation factor Xa, attenuates adverse cardiac remodeling in rats by regulating the PAR-2 and TGF-β1 signaling pathways
Source: PeerJ. 2023 Sep 27;11:e16097. doi: 10.7717/peerj.16097 (PMC10541813; doi:10.7717/peerj.16097)
Supplement: Supplemental Information 2 [file peerj-11-16097-s002.zip › Raw data for western blots/raw data for Figure 3C/Data description for Figure 3C.docx]

Description for Figure 3C Data

The data for the Western blot composite of Figure 3C are from file – collagen I (lanes 1, 2 ,3,4,5 and 6 From left to right, corresponding to Sham, Sham+RIV, Sham+ FSLLRY, LAD, LAD +RIV, and LAD +FSLLRY), file collagen III (anes 1, 2 ,3,4,5 and 6 From left to right, corresponding to Sham, Sham+RIV, Sham+ FSLLRY, LAD, LAD +RIV, and LAD +FSLLRY) and file α-SMA (anes 1, 2 ,3,4,5 and 6 From left to right, corresponding to Sham, Sham+RIV, Sham+ FSLLRY, LAD, LAD +RIV, and LAD +FSLLRY). The images of A B C in each file represent three replicate experiments and their GAPDHs, respectively.

Statistical data comparisons were obtained from 3 independent replicate western blot experiments with duplicates of collagen I, collagen III, α-SMA and GAPDH. grey values of the duplicate data strips for western blot were calculated from image J and were calculated as follows. In the software，1.Image-Type-8bit，2.Process-Subtract Background: Light background. Rolling ball radius:50.0 pixel,3.Analyze-Set Measurements: Area; Min＆max gray value; Integrated density; Mean gray value,4.Analyze-set scale-Distance in pixel:0,Known distance:0,Pixel aspect ratio:1.0,Unit of length: pixel; 5.Edit-Invert-choice the target strip- analyze，and export as the data file Excel. Histograms are exported by Prism8.
