# Supplementary material for: Rivaroxaban, a direct inhibitor of coagulation factor Xa, attenuates adverse cardiac remodeling in rats by regulating the PAR-2 and TGF-β1 signaling pathways
Source: PeerJ. 2023 Sep 27;11:e16097. doi: 10.7717/peerj.16097 (PMC10541813; doi:10.7717/peerj.16097)
Supplement: Supplemental Information 2 [file peerj-11-16097-s002.zip › Raw data for western blots/raw data for Figure 8A/Data description for Figure 8A.docx]

Description for Figure 8A Data

The data for the Western blot composite of Figure 8A are from file – PAR2 (lanes 1, 2 ,3and 4, From left to right, corresponding to Sham, MI, RIV and FSLLRY), file TGF-β (lanes 1, 2 ,3,4and 5, From left to right, corresponding to Control, AngII, RIV +AngII , FSLLRY+AngII and RIV +PAR2-AP+AngII) , file p-Smad2 (lanes 1, 2 ,3,4and 5, From left to right, corresponding to Control, AngII, RIV +AngII , FSLLRY+AngII and RIV +PAR2-AP+AngII) and file p-Smad3(lanes 1, 2 ,3,4and 5, From left to right, corresponding to Control, AngII, RIV +AngII , FSLLRY+AngII and RIV +PAR2-AP+AngII). The images of A B C in each file represent three replicate experiments and their GAPDH, respectively. We are so sorry that in the course of our experiments, the gel images of p-smad2 have been cropped due to the strong background of the Mixed bands. However, we provide membrane prior to incubation with ECL in this file all the original blot images with markers (A-raw, B-raw,C-raw). Our team guarantees that all data are true, objective and reliable.

Statistical data comparisons were obtained from 3 independent replicate western blot experiments with duplicates of PAR2, TGF-β, p-Smad2 and p-Smad3. grey values of the duplicate data strips for western blot were calculated from image J and were calculated as follows. In the software，1.Image-Type-8bit，2.Process-Subtract Background: Light background. Rolling ball radius:50.0 pixel,3.Analyze-Set Measurements: Area; Min＆max gray value; Integrated density; Mean gray value,4.Analyze-set scale-Distance in pixel:0,Known distance:0,Pixel aspect ratio:1.0,Unit of length: pixel; 5.Edit-Invert-choice the target strip- analyze，and export as the data file Excel. Histograms are exported by Prism8.
