# Supplementary figures and images for: Rivaroxaban, a direct inhibitor of coagulation factor Xa, attenuates adverse cardiac remodeling in rats by regulating the PAR-2 and TGF-β1 signaling pathways (part 1 of 2)
Source: PeerJ. 2023 Sep 27;11:e16097. doi: 10.7717/peerj.16097 (PMC10541813; doi:10.7717/peerj.16097)

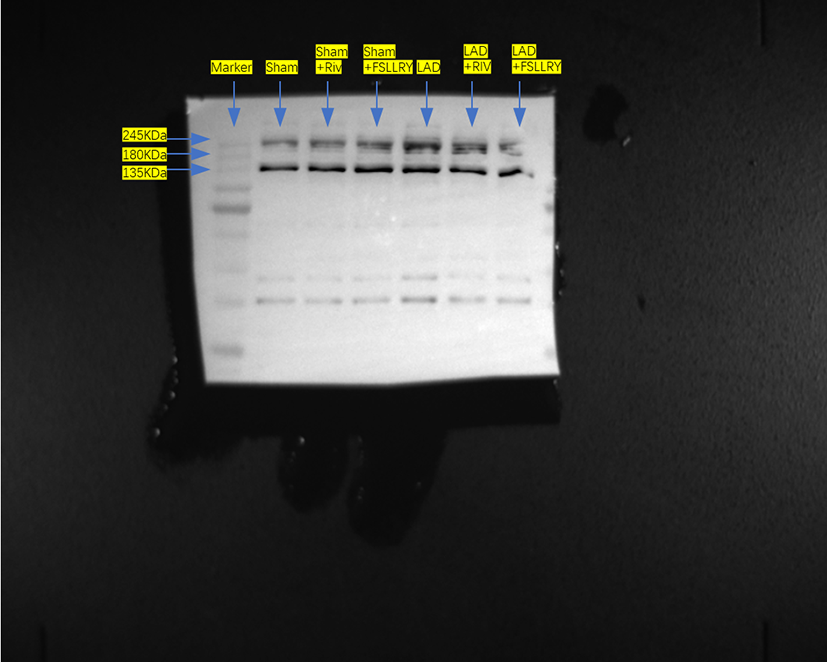

Supplement: Supplemental Information 2 [file peerj-11-16097-s002.zip › Raw data for western blots/raw data for Figure 3C/Collagen I/A.TIF]

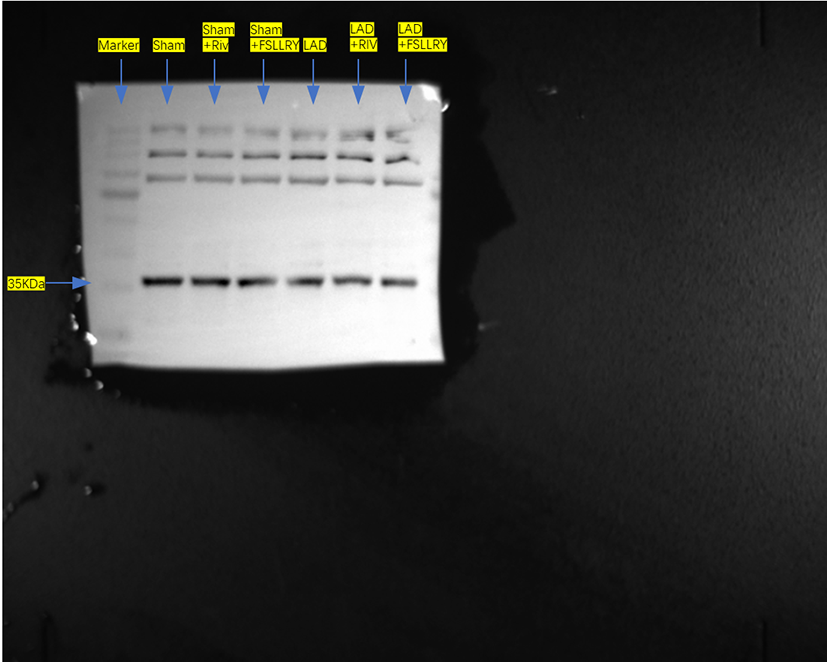

Supplement: Supplemental Information 2 [file peerj-11-16097-s002.zip › Raw data for western blots/raw data for Figure 3C/Collagen I/A+GAPDH.TIF]

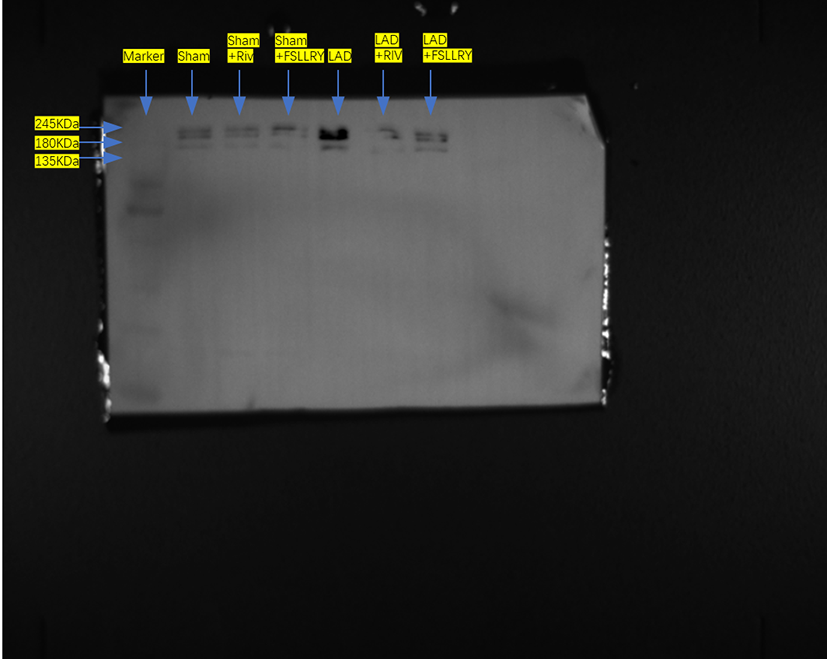

Supplement: Supplemental Information 2 [file peerj-11-16097-s002.zip › Raw data for western blots/raw data for Figure 3C/Collagen I/B.TIF]

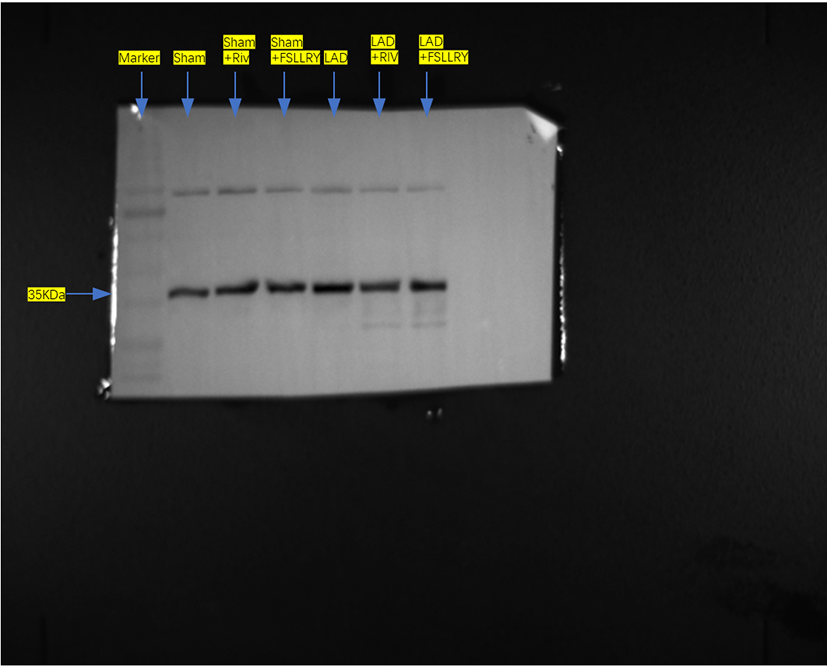

Supplement: Supplemental Information 2 [file peerj-11-16097-s002.zip › Raw data for western blots/raw data for Figure 3C/Collagen I/B+GAPDH.TIF]

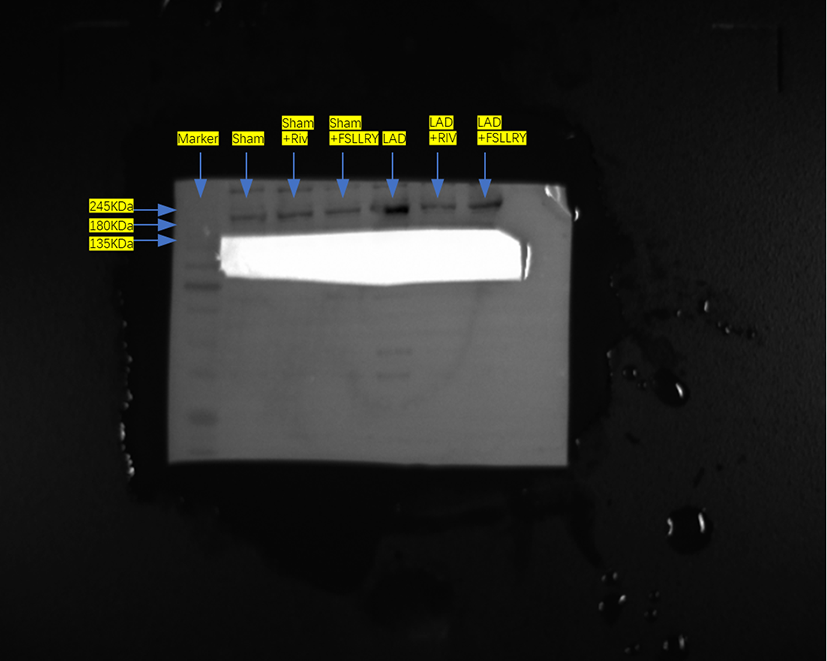

Supplement: Supplemental Information 2 [file peerj-11-16097-s002.zip › Raw data for western blots/raw data for Figure 3C/Collagen I/C.TIF]

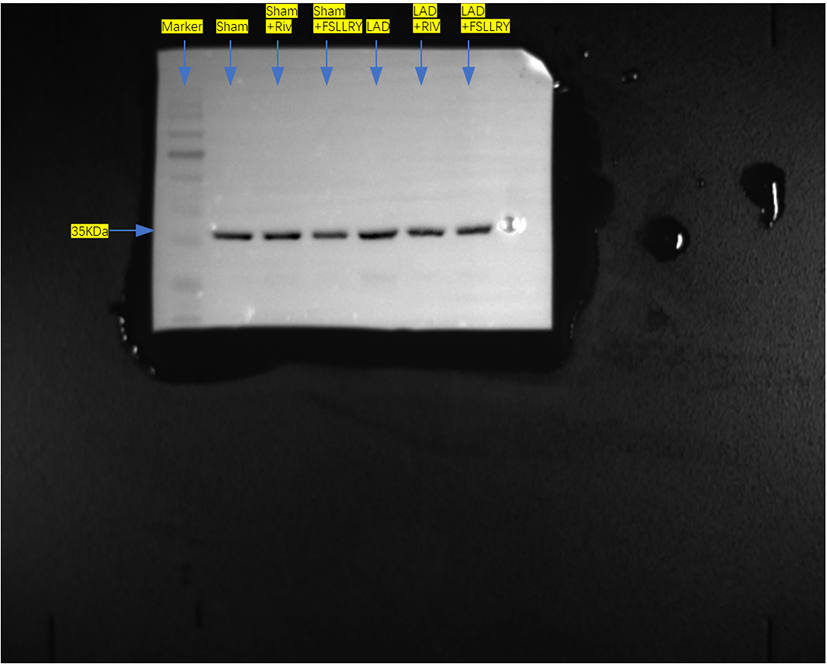

Supplement: Supplemental Information 2 [file peerj-11-16097-s002.zip › Raw data for western blots/raw data for Figure 3C/Collagen I/C+GAPDH.TIF]

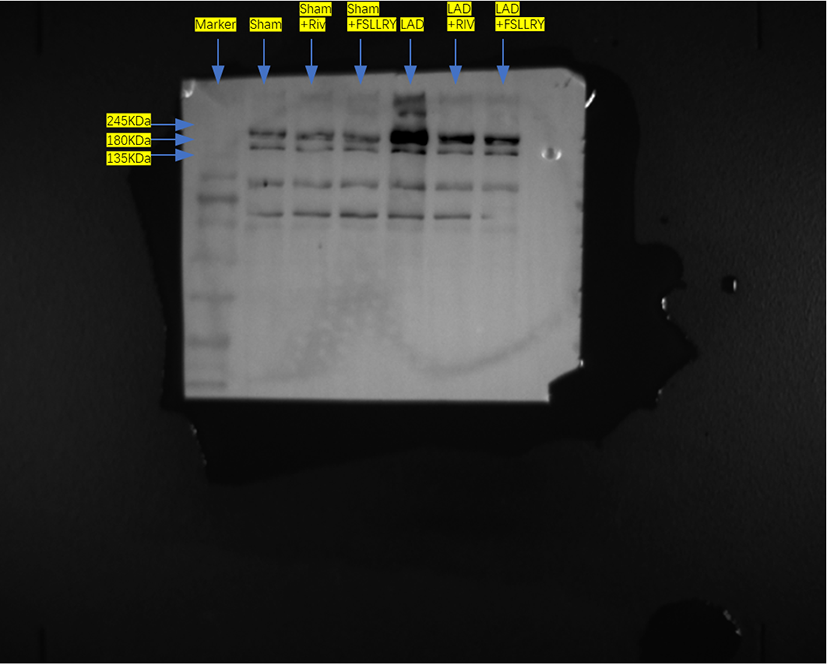

Supplement: Supplemental Information 2 [file peerj-11-16097-s002.zip › Raw data for western blots/raw data for Figure 3C/Collagen III/A.TIF]

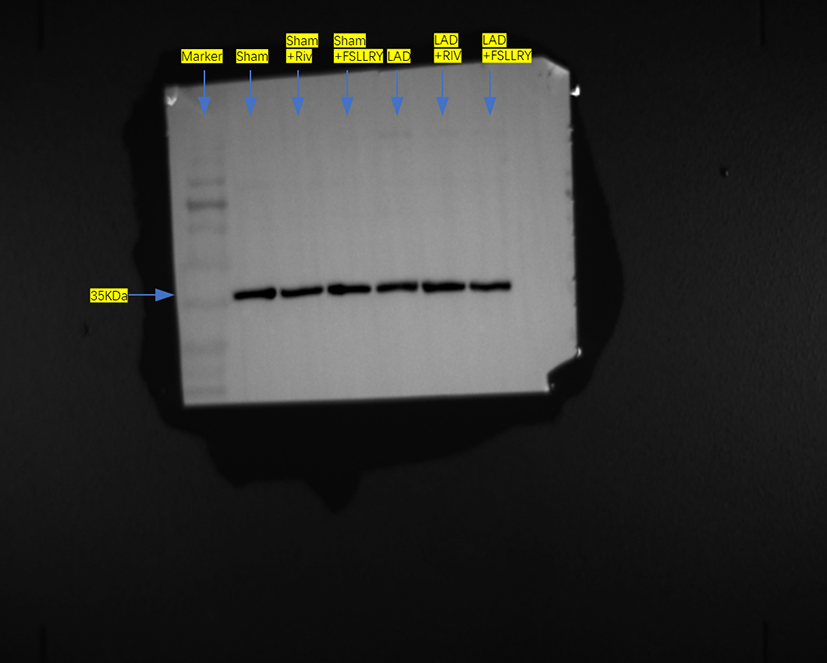

Supplement: Supplemental Information 2 [file peerj-11-16097-s002.zip › Raw data for western blots/raw data for Figure 3C/Collagen III/A+GAPDH.TIF]

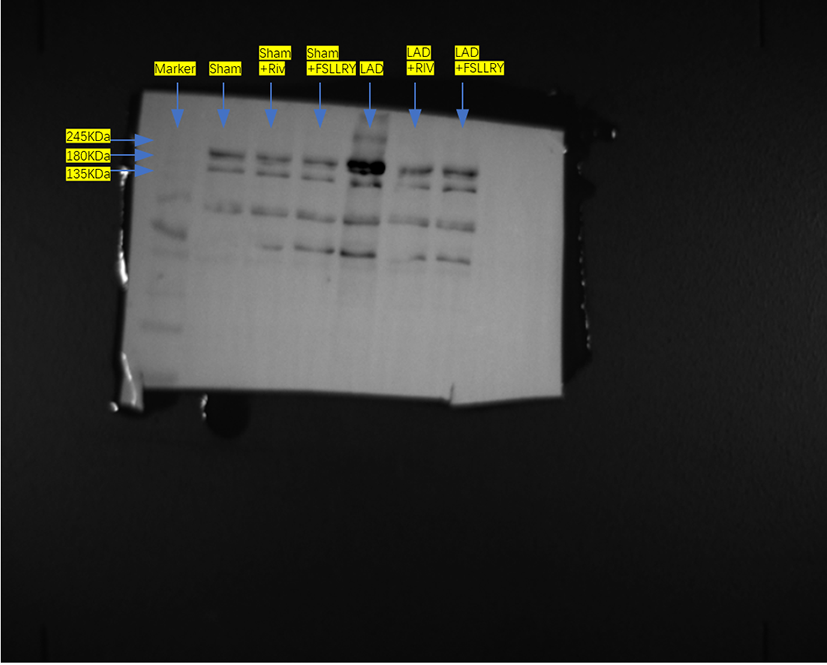

Supplement: Supplemental Information 2 [file peerj-11-16097-s002.zip › Raw data for western blots/raw data for Figure 3C/Collagen III/B.TIF]

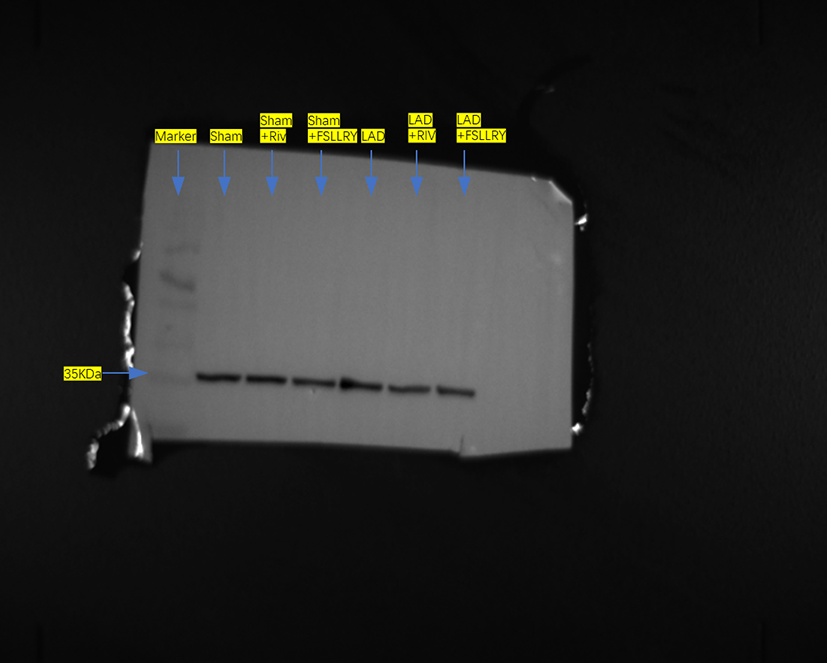

Supplement: Supplemental Information 2 [file peerj-11-16097-s002.zip › Raw data for western blots/raw data for Figure 3C/Collagen III/B+GAPDH.TIF]

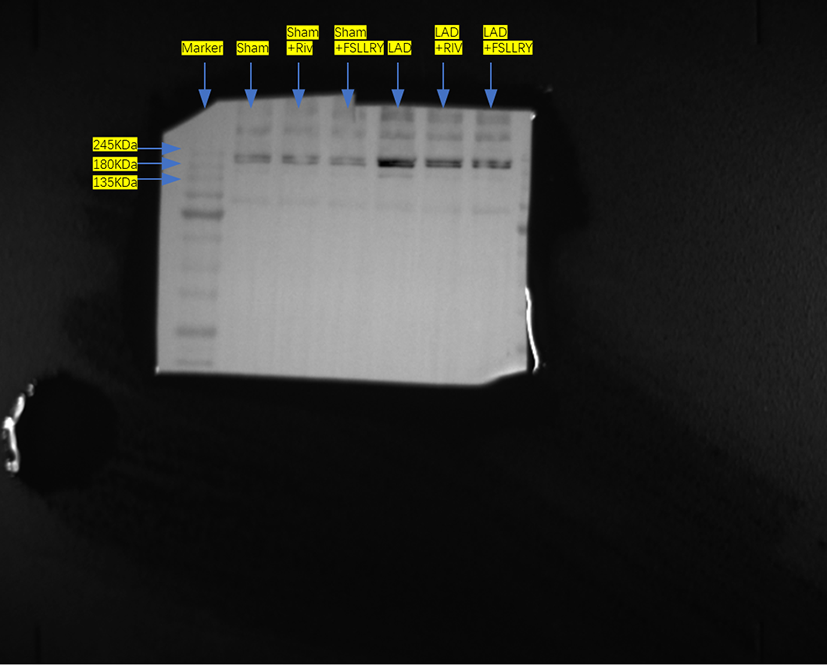

Supplement: Supplemental Information 2 [file peerj-11-16097-s002.zip › Raw data for western blots/raw data for Figure 3C/Collagen III/C.TIF]

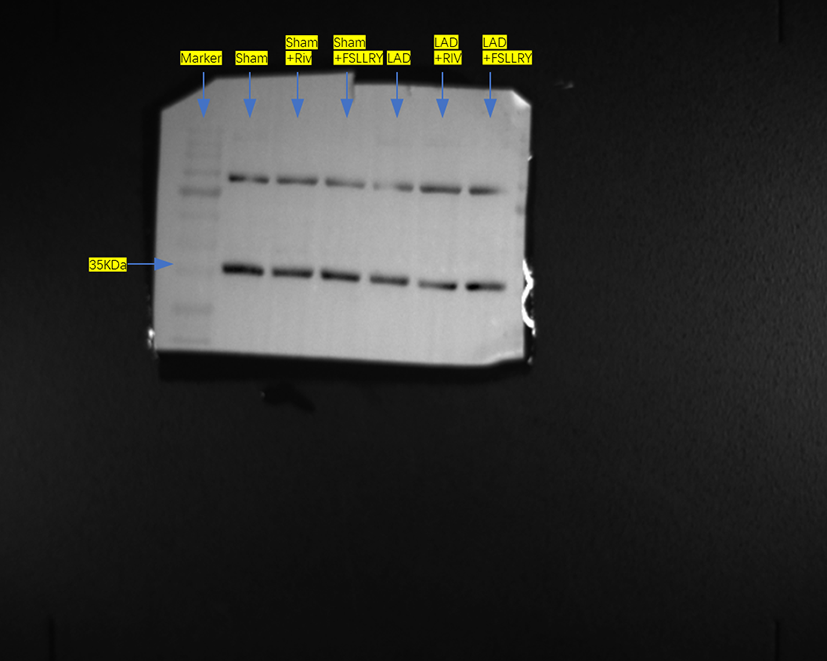

Supplement: Supplemental Information 2 [file peerj-11-16097-s002.zip › Raw data for western blots/raw data for Figure 3C/Collagen III/C+GAPDH.TIF]

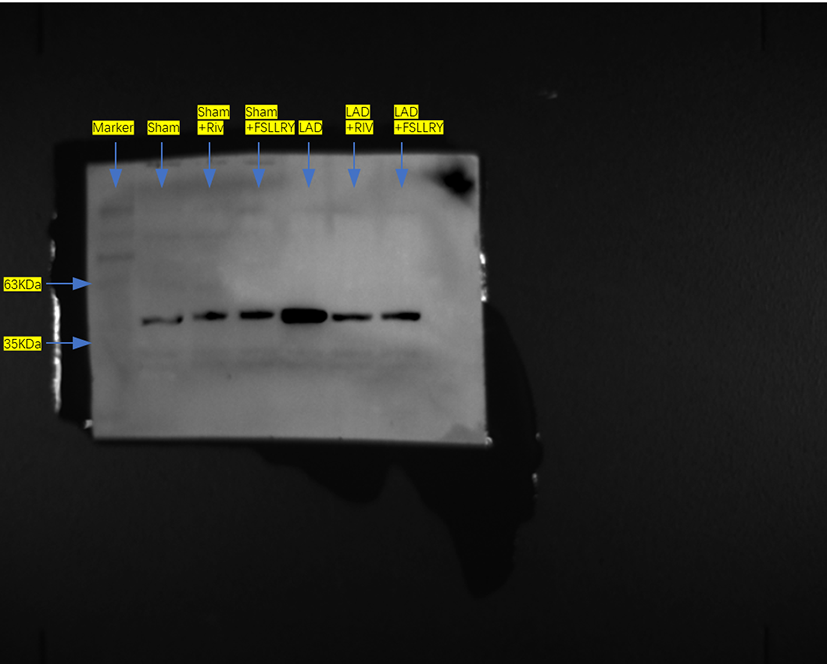

Supplement: Supplemental Information 2 [file peerj-11-16097-s002.zip › Raw data for western blots/raw data for Figure 3C/a┴-SMA/A.TIF]

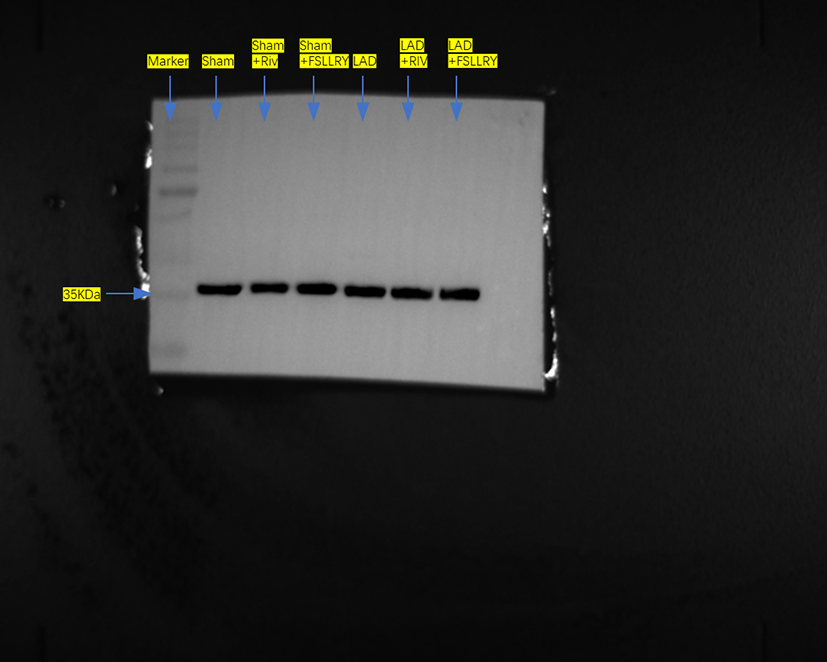

Supplement: Supplemental Information 2 [file peerj-11-16097-s002.zip › Raw data for western blots/raw data for Figure 3C/a┴-SMA/A+GAPDH.TIF]

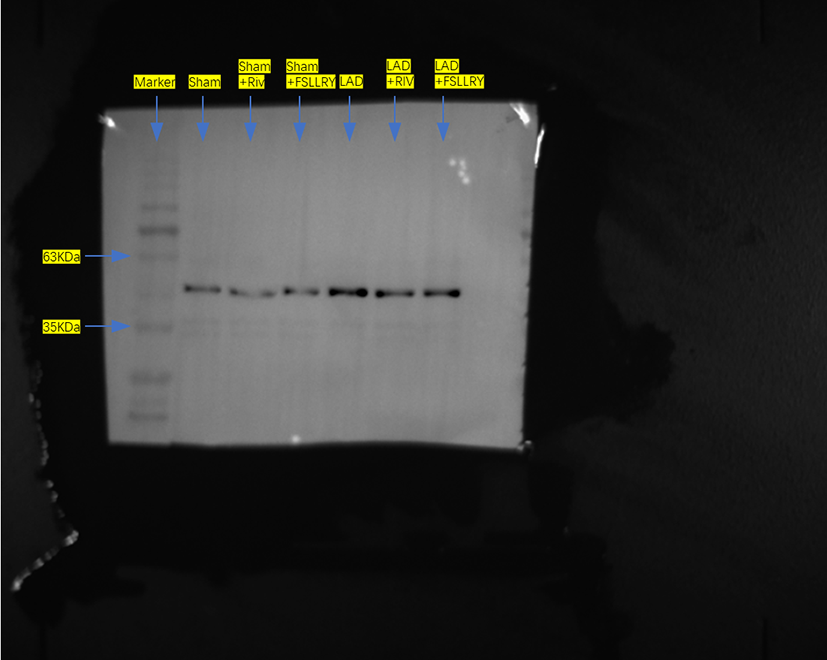

Supplement: Supplemental Information 2 [file peerj-11-16097-s002.zip › Raw data for western blots/raw data for Figure 3C/a┴-SMA/B.TIF]

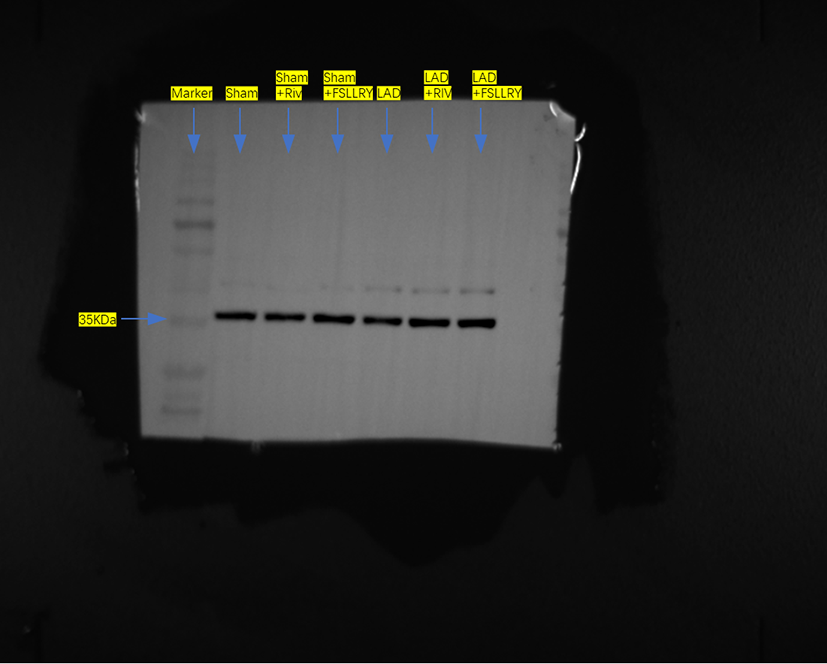

Supplement: Supplemental Information 2 [file peerj-11-16097-s002.zip › Raw data for western blots/raw data for Figure 3C/a┴-SMA/B+GAPDH.TIF]

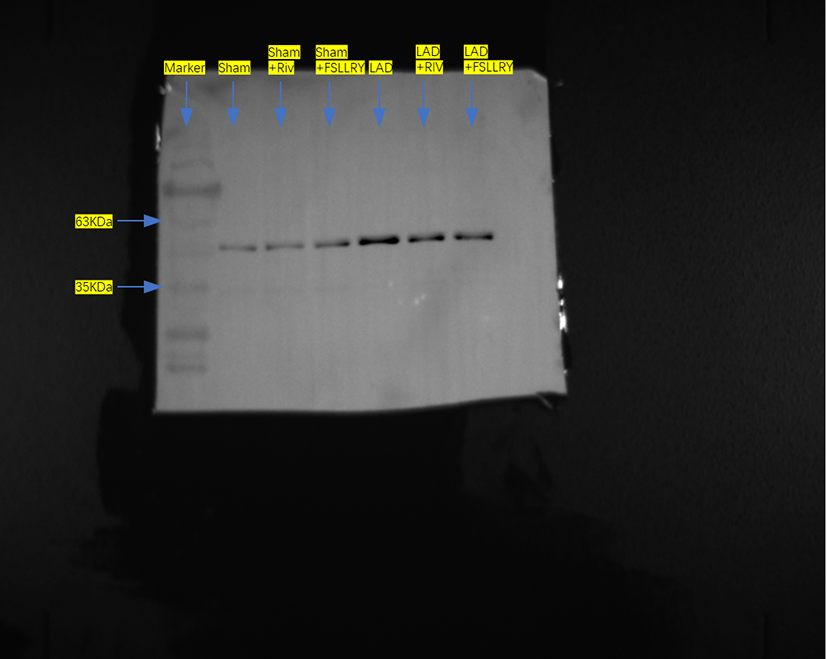

Supplement: Supplemental Information 2 [file peerj-11-16097-s002.zip › Raw data for western blots/raw data for Figure 3C/a┴-SMA/C.TIF]

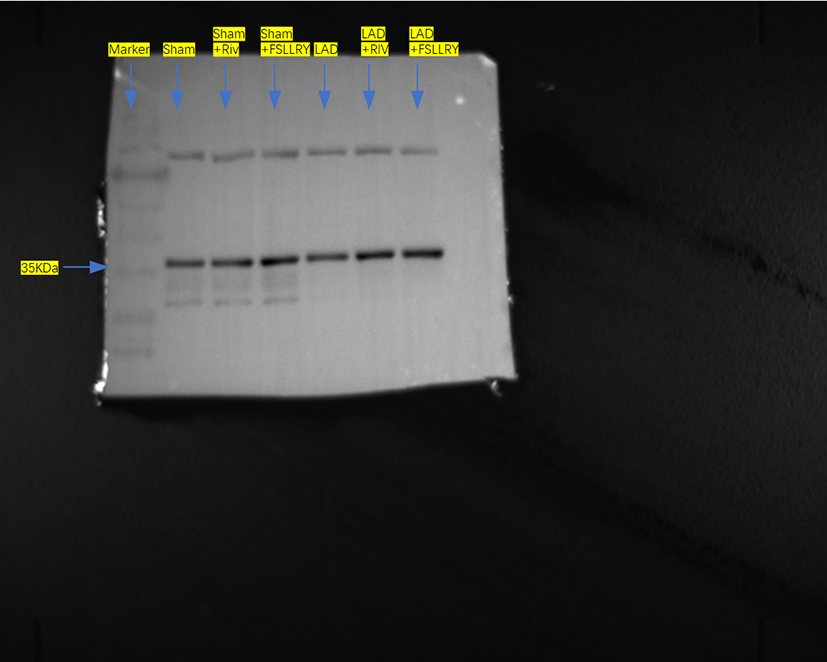

Supplement: Supplemental Information 2 [file peerj-11-16097-s002.zip › Raw data for western blots/raw data for Figure 3C/a┴-SMA/C+GAPDH.TIF]

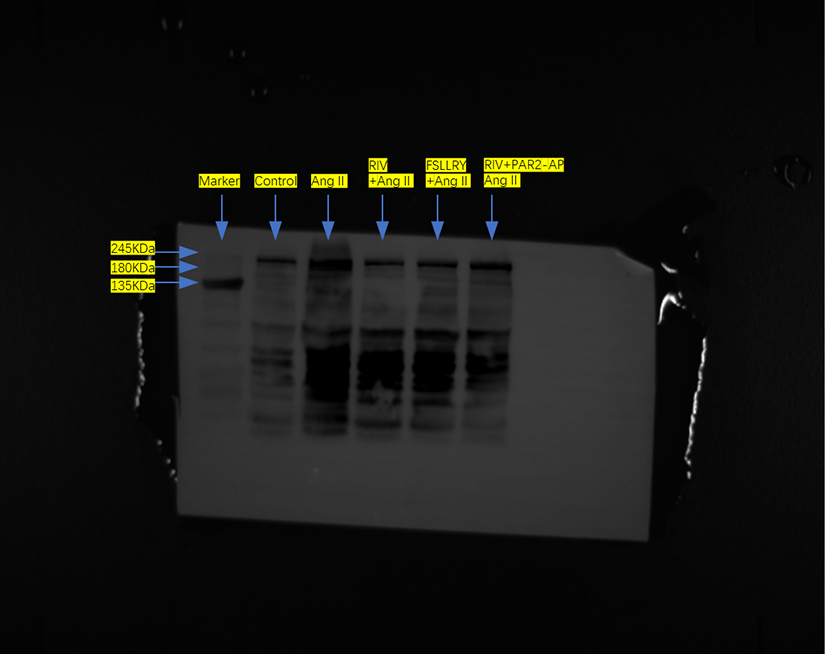

Supplement: Supplemental Information 2 [file peerj-11-16097-s002.zip › Raw data for western blots/raw data for Figure 6B/col1/A.TIF]

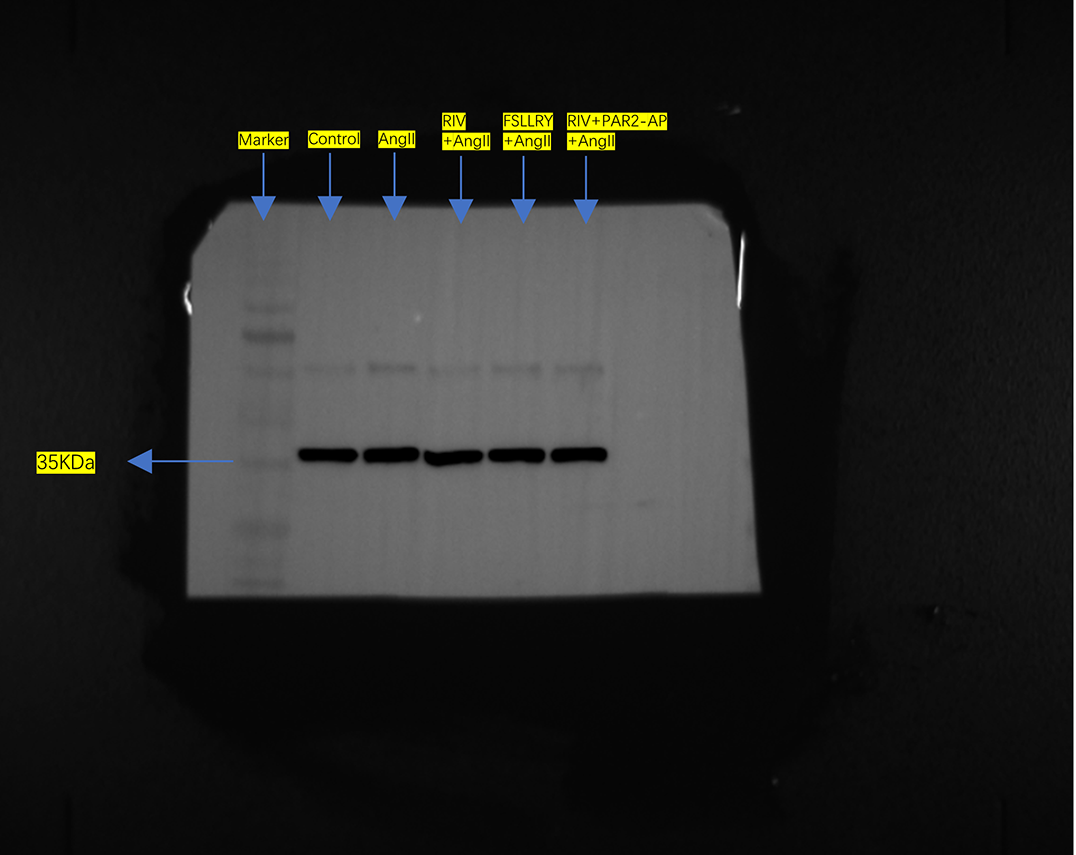

Supplement: Supplemental Information 2 [file peerj-11-16097-s002.zip › Raw data for western blots/raw data for Figure 6B/col1/A+GAPDH.TIF]

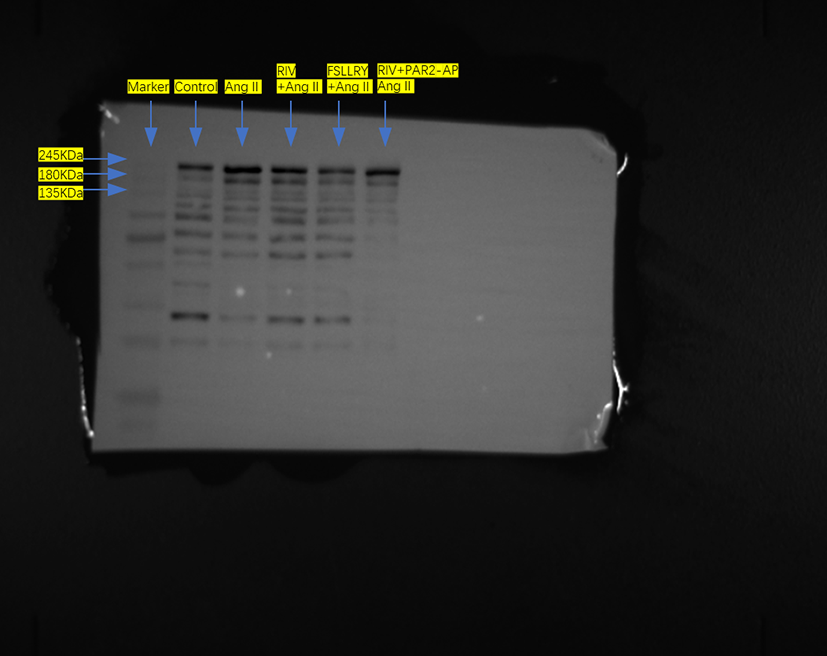

Supplement: Supplemental Information 2 [file peerj-11-16097-s002.zip › Raw data for western blots/raw data for Figure 6B/col1/B.TIF]

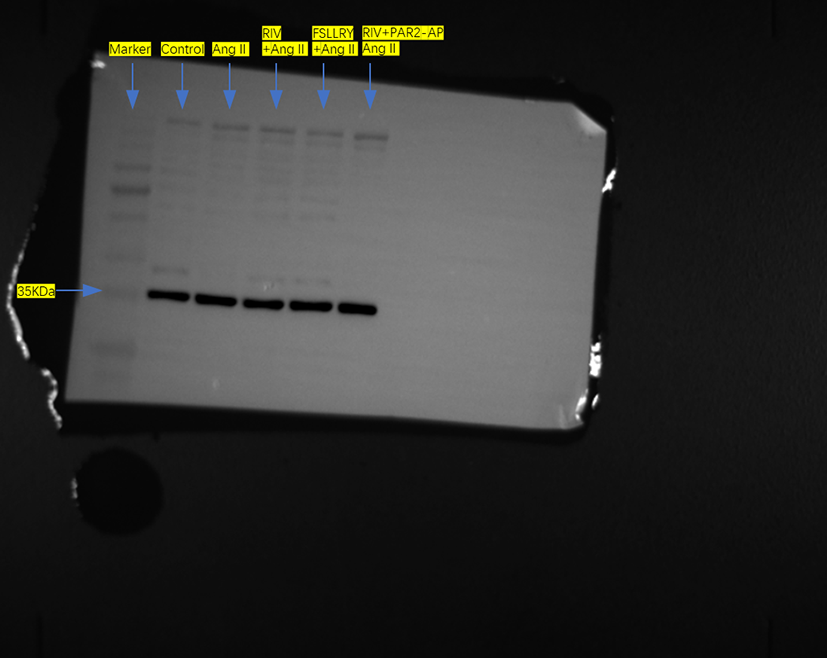

Supplement: Supplemental Information 2 [file peerj-11-16097-s002.zip › Raw data for western blots/raw data for Figure 6B/col1/B+GAPDH.TIF]

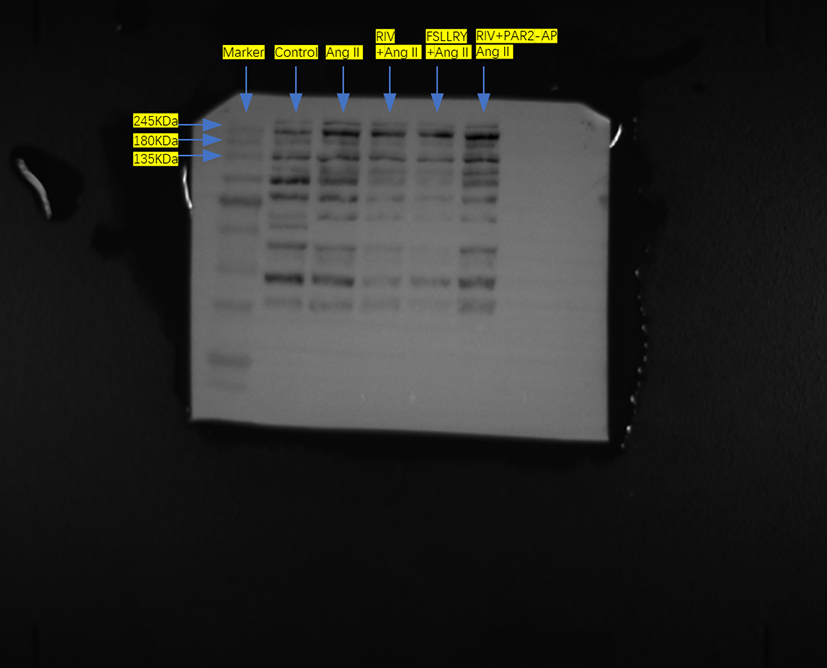

Supplement: Supplemental Information 2 [file peerj-11-16097-s002.zip › Raw data for western blots/raw data for Figure 6B/col1/C.TIF]

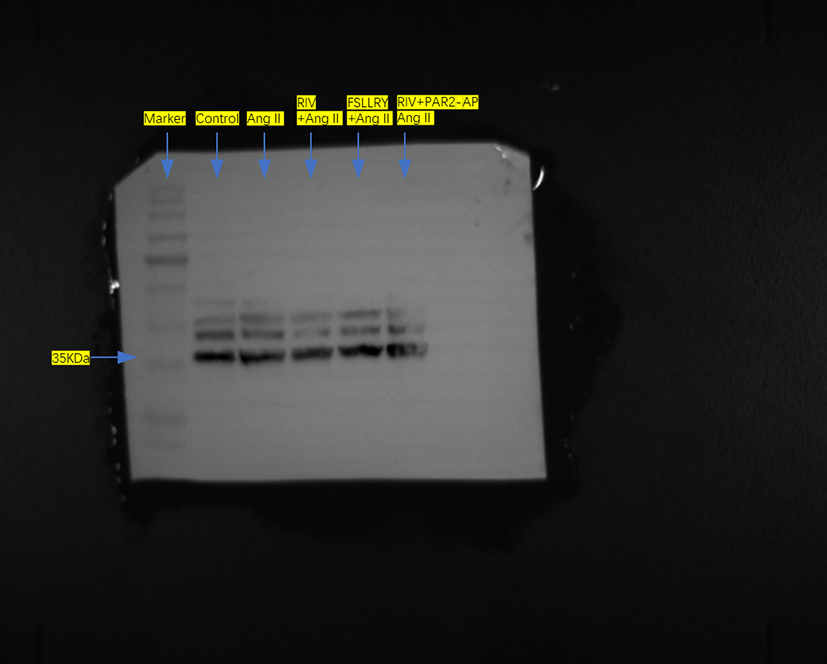

Supplement: Supplemental Information 2 [file peerj-11-16097-s002.zip › Raw data for western blots/raw data for Figure 6B/col1/C+GAPDH.TIF]

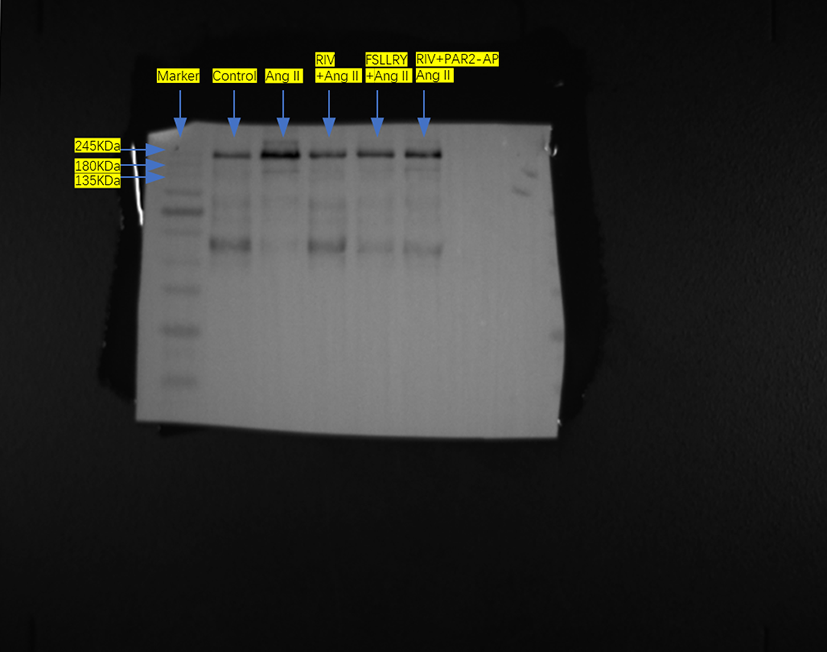

Supplement: Supplemental Information 2 [file peerj-11-16097-s002.zip › Raw data for western blots/raw data for Figure 6B/col3/A.TIF]

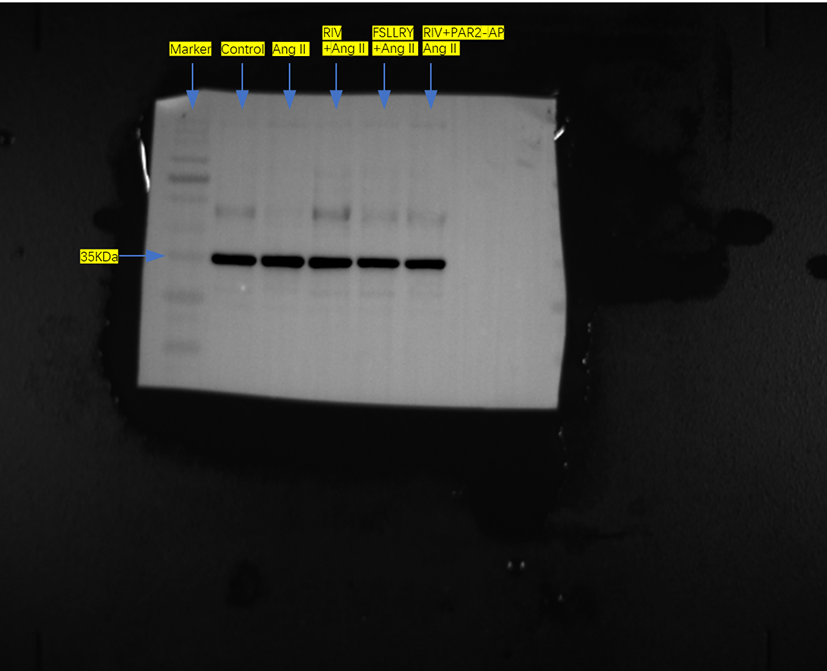

Supplement: Supplemental Information 2 [file peerj-11-16097-s002.zip › Raw data for western blots/raw data for Figure 6B/col3/A+GAPDH.TIF]

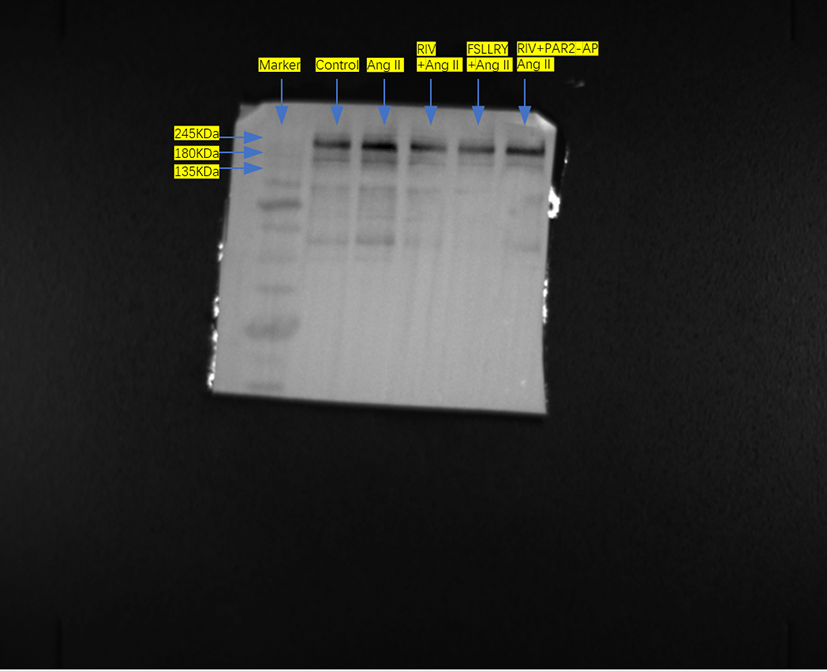

Supplement: Supplemental Information 2 [file peerj-11-16097-s002.zip › Raw data for western blots/raw data for Figure 6B/col3/B.TIF]

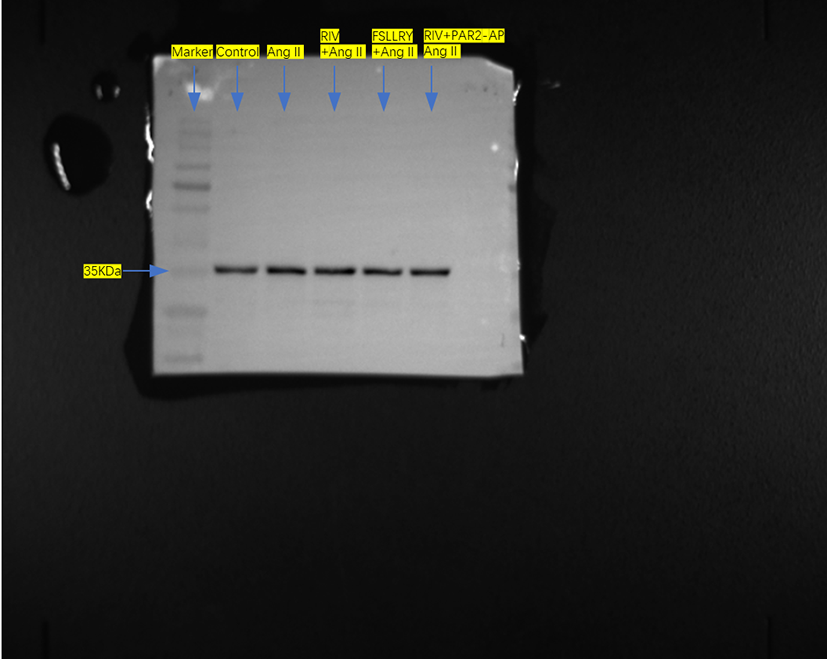

Supplement: Supplemental Information 2 [file peerj-11-16097-s002.zip › Raw data for western blots/raw data for Figure 6B/col3/B+GAPDH.TIF]

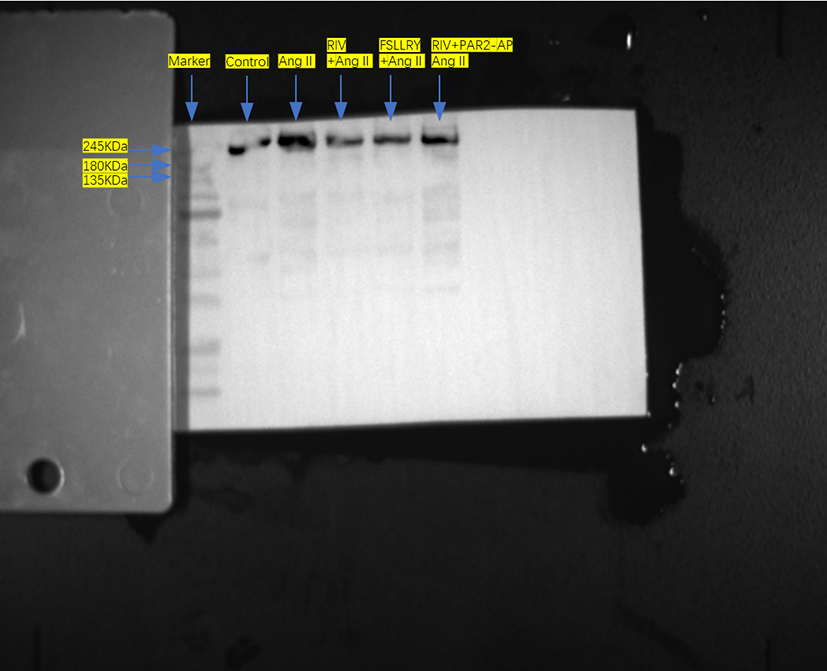

Supplement: Supplemental Information 2 [file peerj-11-16097-s002.zip › Raw data for western blots/raw data for Figure 6B/col3/C.TIF]

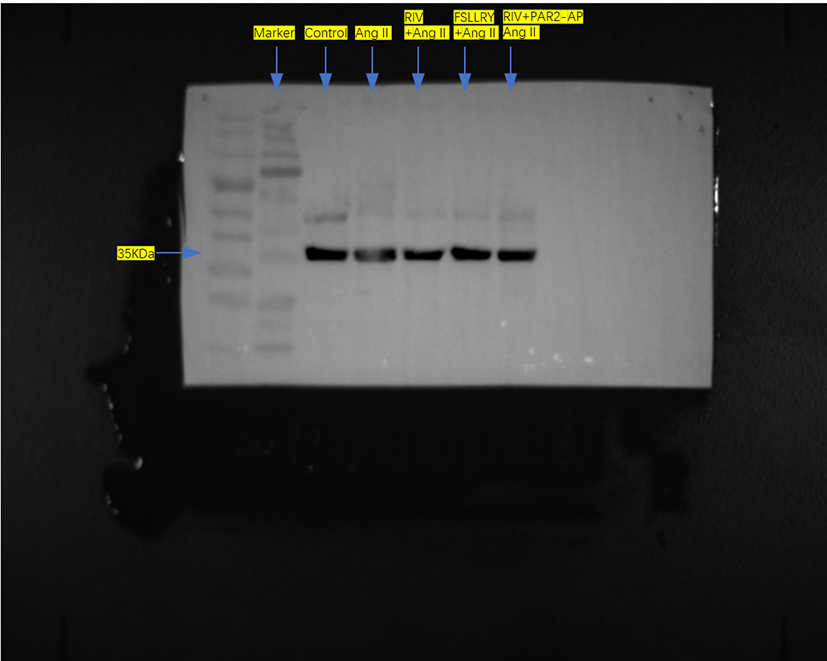

Supplement: Supplemental Information 2 [file peerj-11-16097-s002.zip › Raw data for western blots/raw data for Figure 6B/col3/C+GAPDH.TIF]

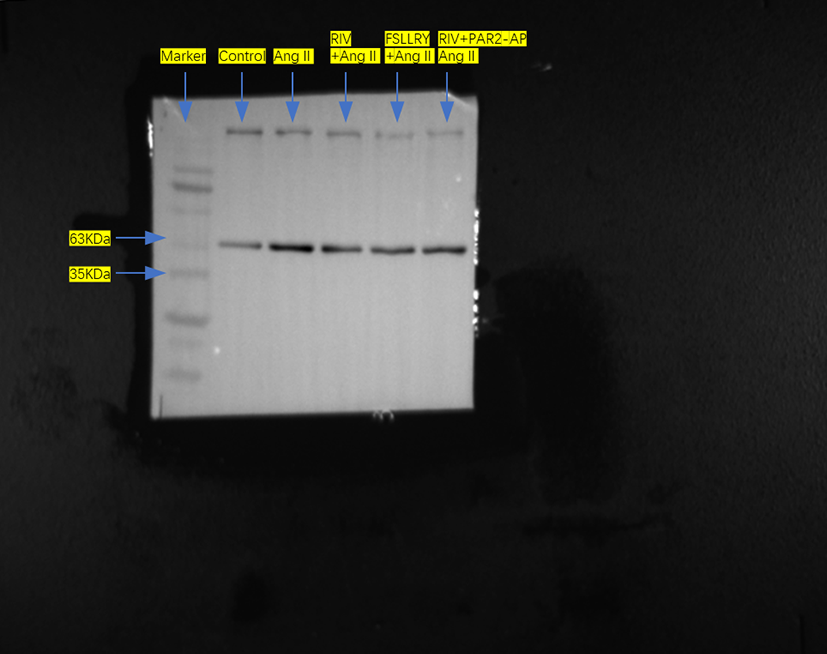

Supplement: Supplemental Information 2 [file peerj-11-16097-s002.zip › Raw data for western blots/raw data for Figure 6B/SMA/A.TIF]

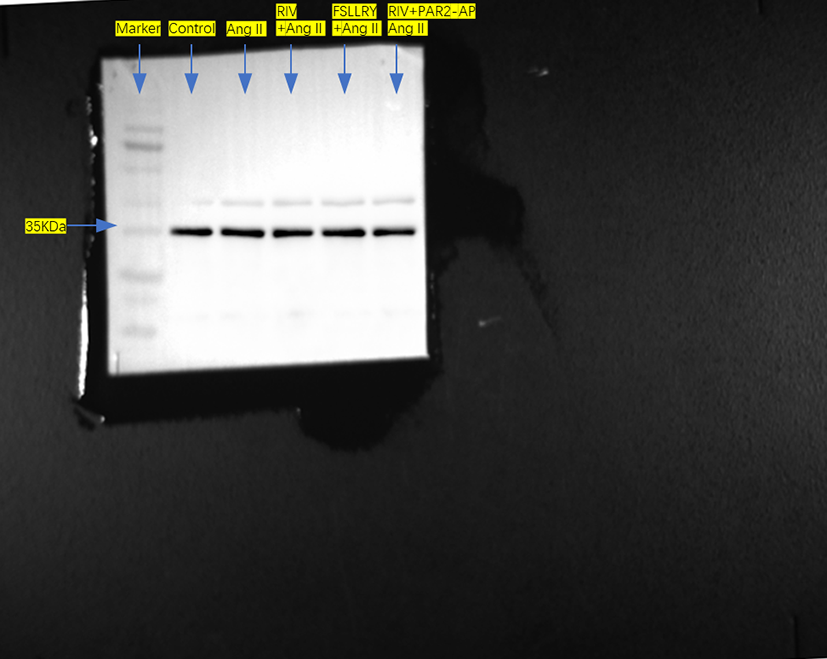

Supplement: Supplemental Information 2 [file peerj-11-16097-s002.zip › Raw data for western blots/raw data for Figure 6B/SMA/A+GAPDH.TIF]

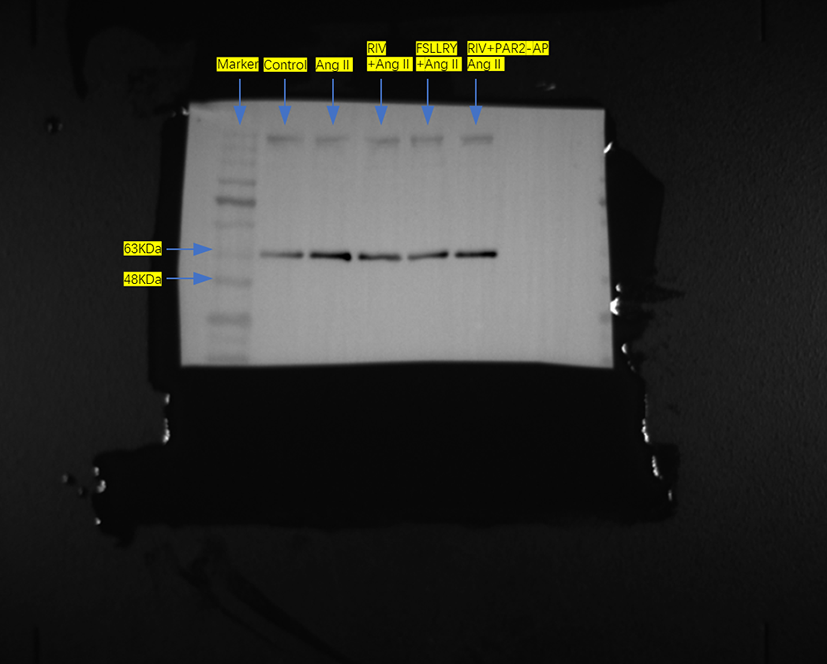

Supplement: Supplemental Information 2 [file peerj-11-16097-s002.zip › Raw data for western blots/raw data for Figure 6B/SMA/B.TIF]

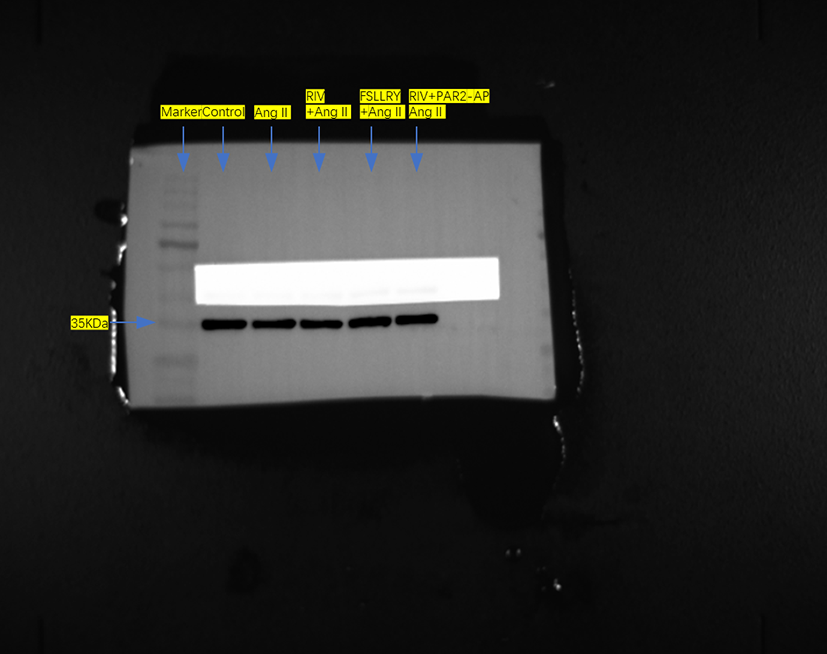

Supplement: Supplemental Information 2 [file peerj-11-16097-s002.zip › Raw data for western blots/raw data for Figure 6B/SMA/B+GAPDH.TIF]

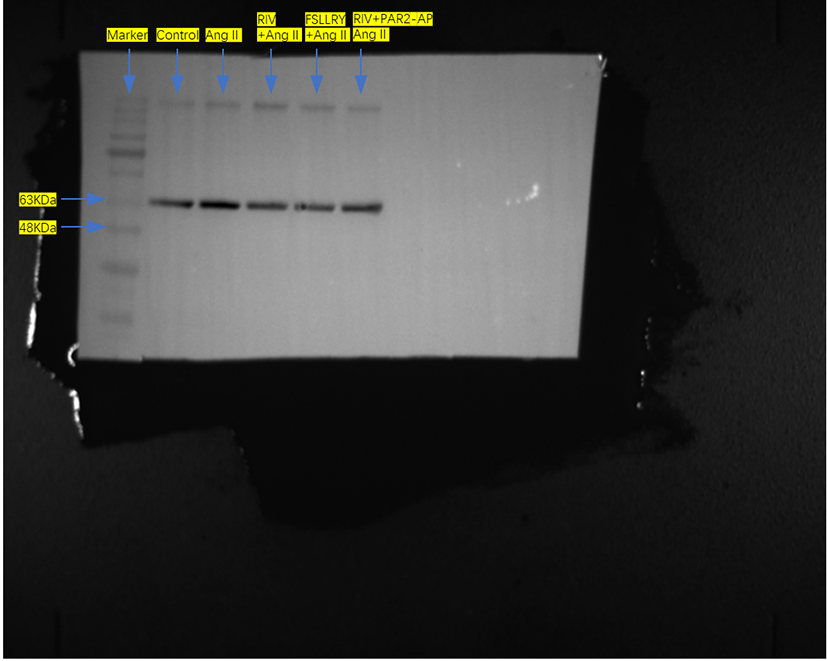

Supplement: Supplemental Information 2 [file peerj-11-16097-s002.zip › Raw data for western blots/raw data for Figure 6B/SMA/C.TIF]

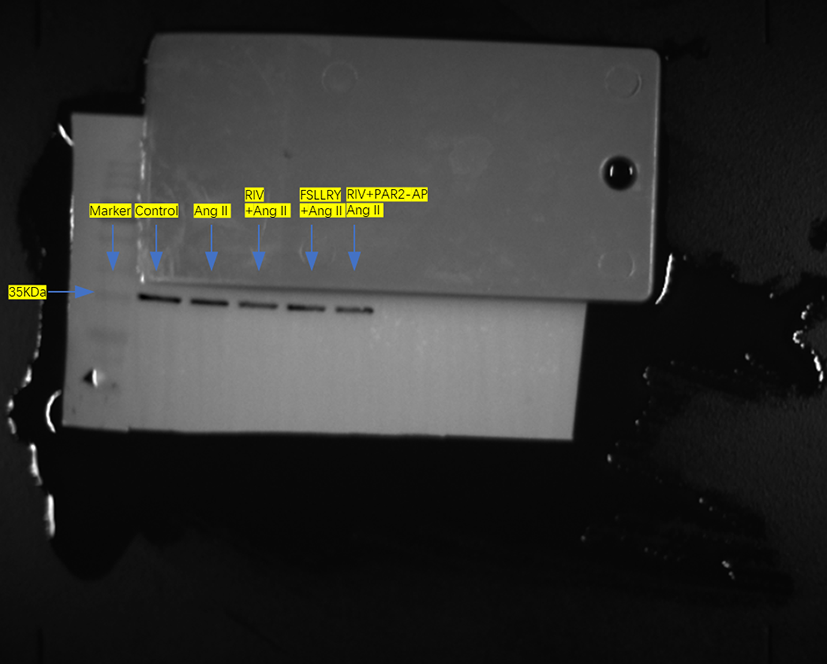

Supplement: Supplemental Information 2 [file peerj-11-16097-s002.zip › Raw data for western blots/raw data for Figure 6B/SMA/C+GAPDH.TIF]

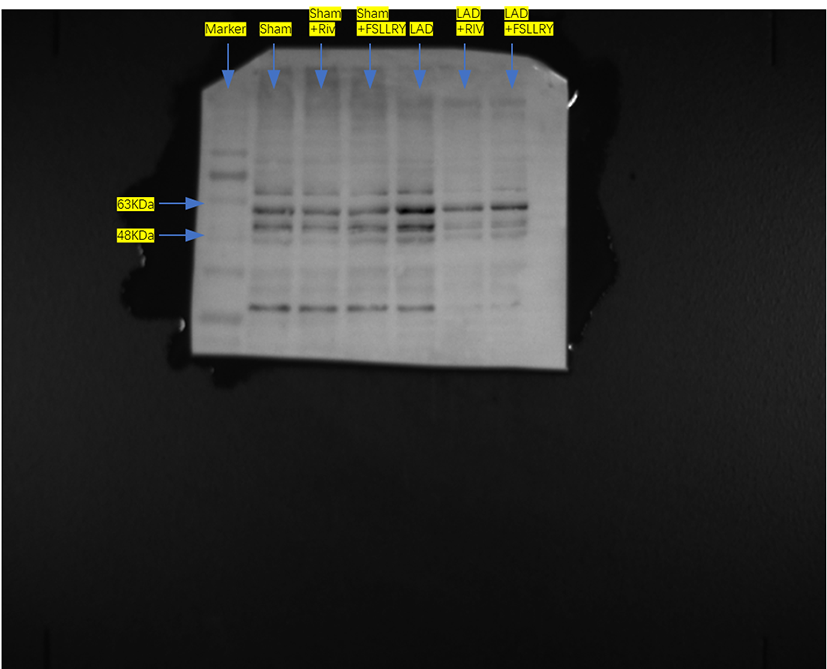

Supplement: Supplemental Information 2 [file peerj-11-16097-s002.zip › Raw data for western blots/raw data for Figure 7B/PAR2/A.TIF]

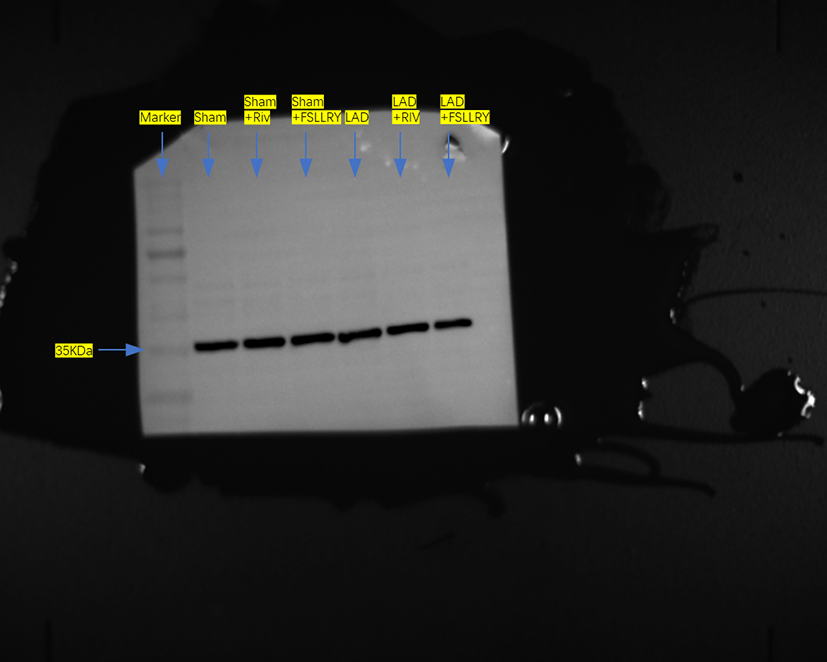

Supplement: Supplemental Information 2 [file peerj-11-16097-s002.zip › Raw data for western blots/raw data for Figure 7B/PAR2/A+GAPDH.TIF]

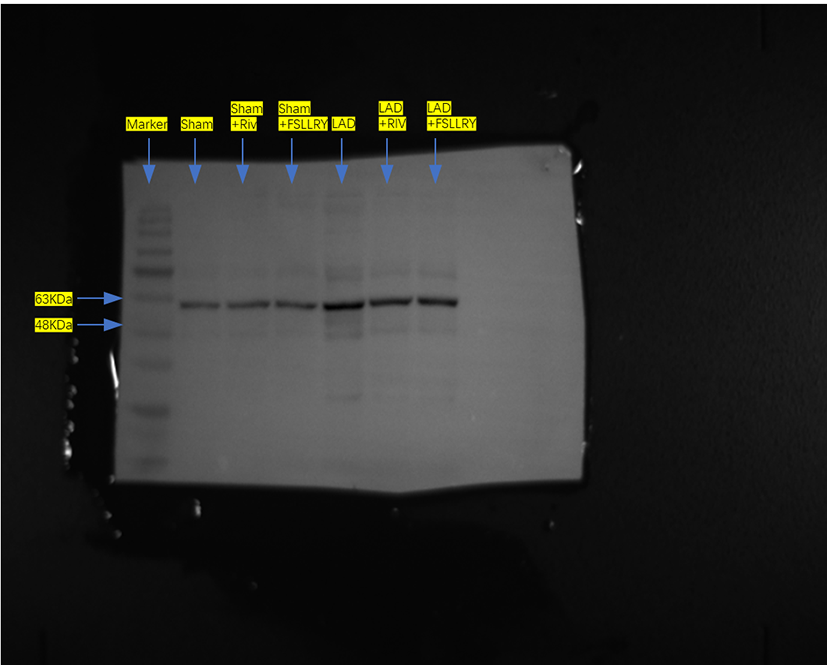

Supplement: Supplemental Information 2 [file peerj-11-16097-s002.zip › Raw data for western blots/raw data for Figure 7B/PAR2/B.TIF]

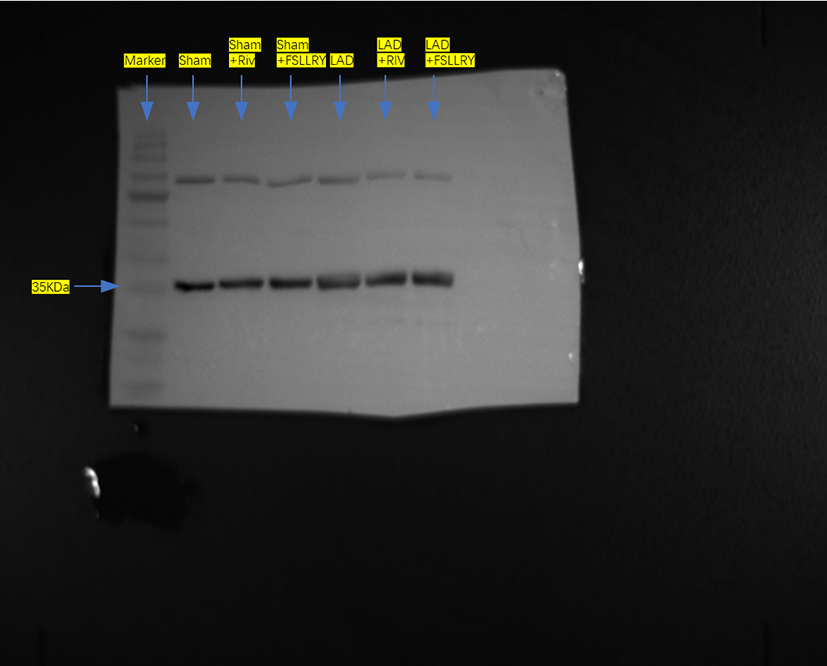

Supplement: Supplemental Information 2 [file peerj-11-16097-s002.zip › Raw data for western blots/raw data for Figure 7B/PAR2/B+GAPDH.TIF]

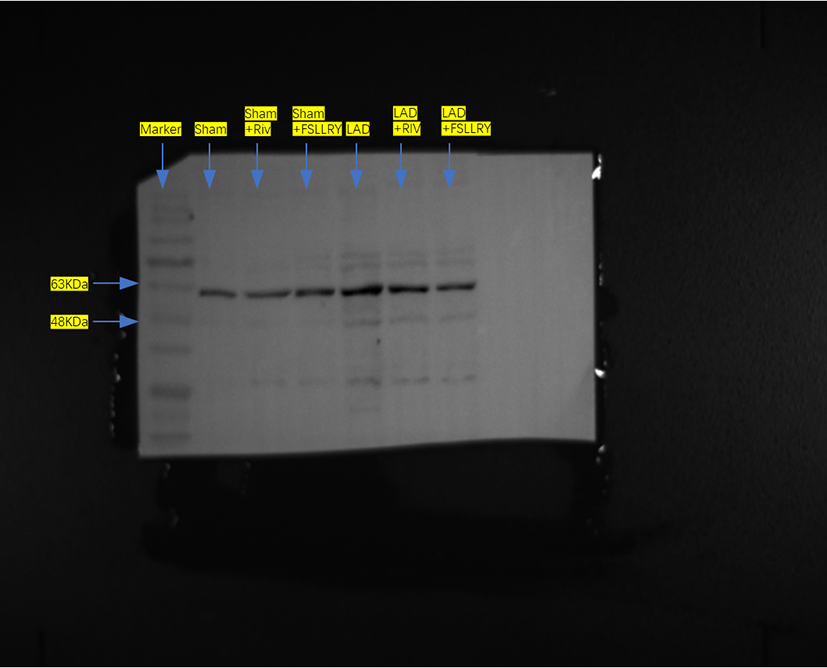

Supplement: Supplemental Information 2 [file peerj-11-16097-s002.zip › Raw data for western blots/raw data for Figure 7B/PAR2/C.TIF]

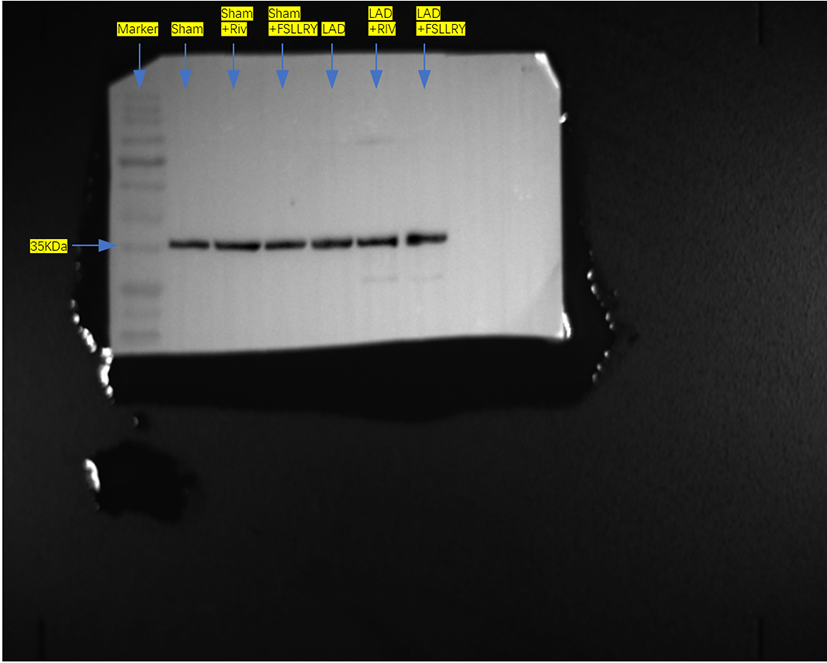

Supplement: Supplemental Information 2 [file peerj-11-16097-s002.zip › Raw data for western blots/raw data for Figure 7B/PAR2/C+GAPDH.TIF]

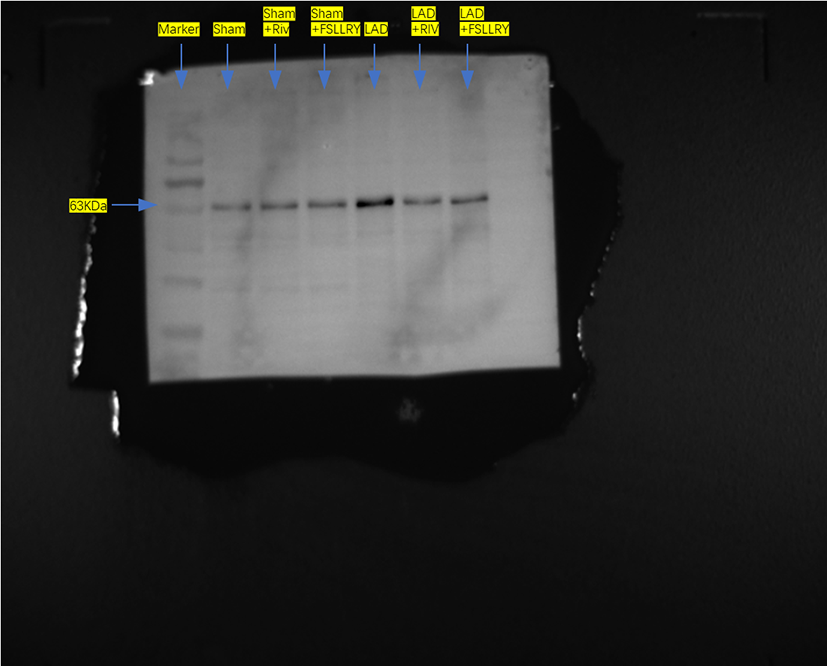

Supplement: Supplemental Information 2 [file peerj-11-16097-s002.zip › Raw data for western blots/raw data for Figure 7B/P-Smad2/A.TIF]

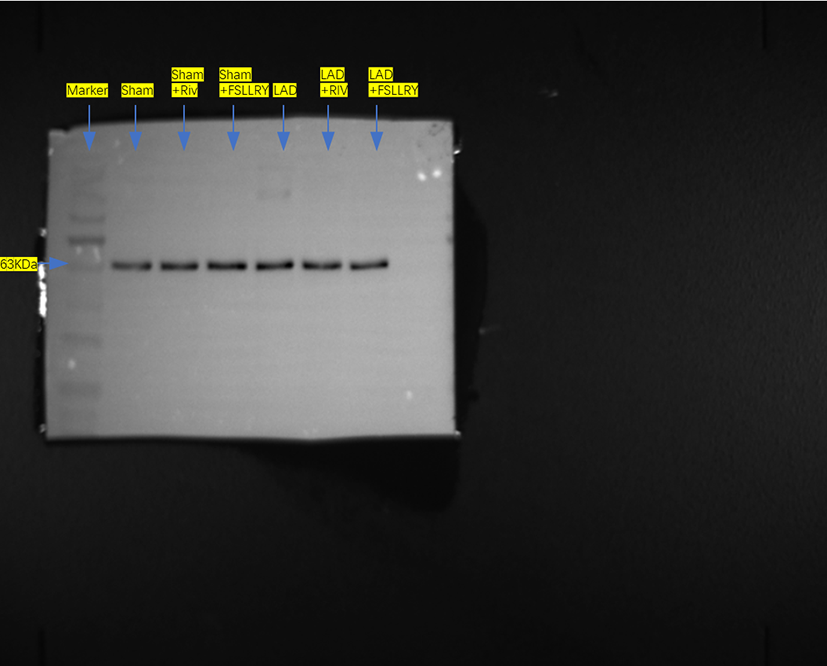

Supplement: Supplemental Information 2 [file peerj-11-16097-s002.zip › Raw data for western blots/raw data for Figure 7B/P-Smad2/A+GAPDH.TIF]

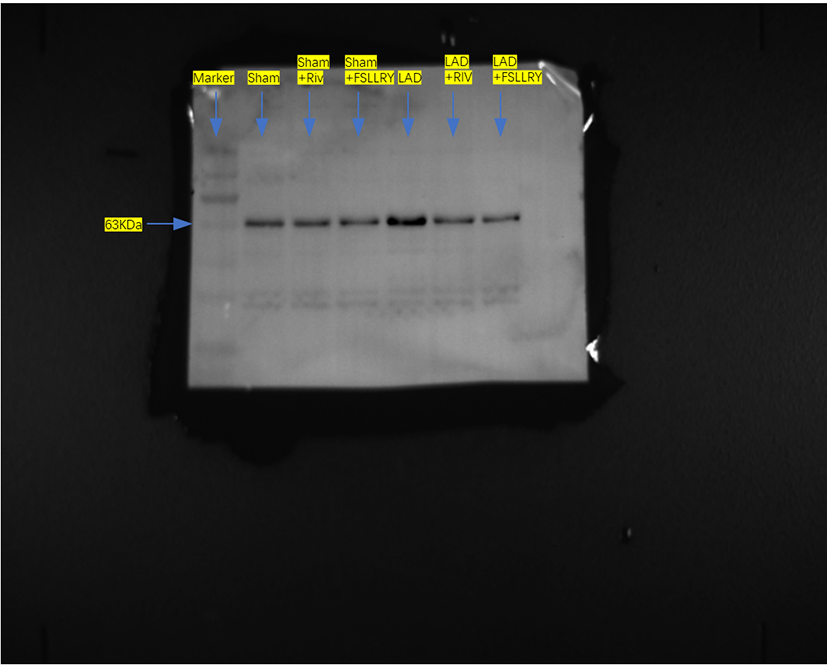

Supplement: Supplemental Information 2 [file peerj-11-16097-s002.zip › Raw data for western blots/raw data for Figure 7B/P-Smad2/B.TIF]

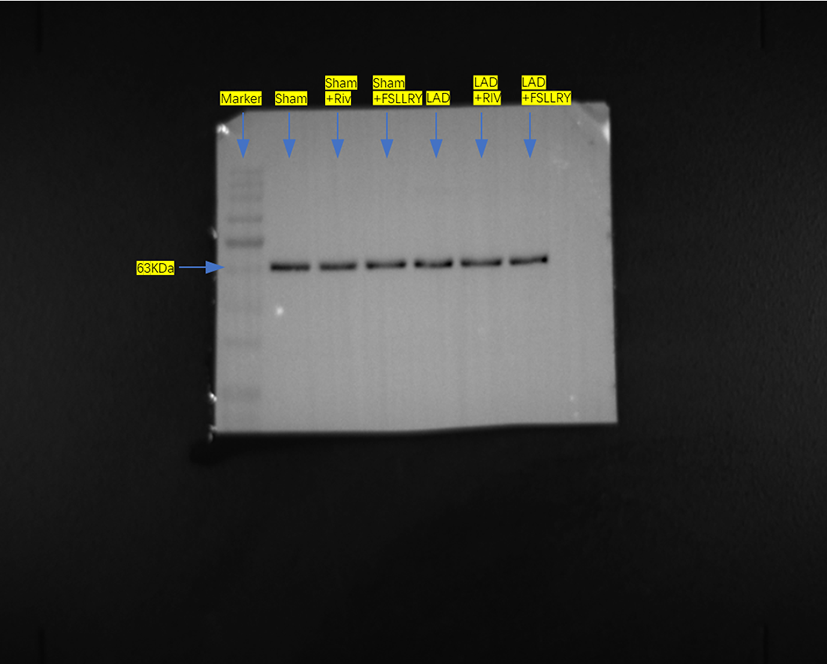

Supplement: Supplemental Information 2 [file peerj-11-16097-s002.zip › Raw data for western blots/raw data for Figure 7B/P-Smad2/B+GAPDH.TIF]

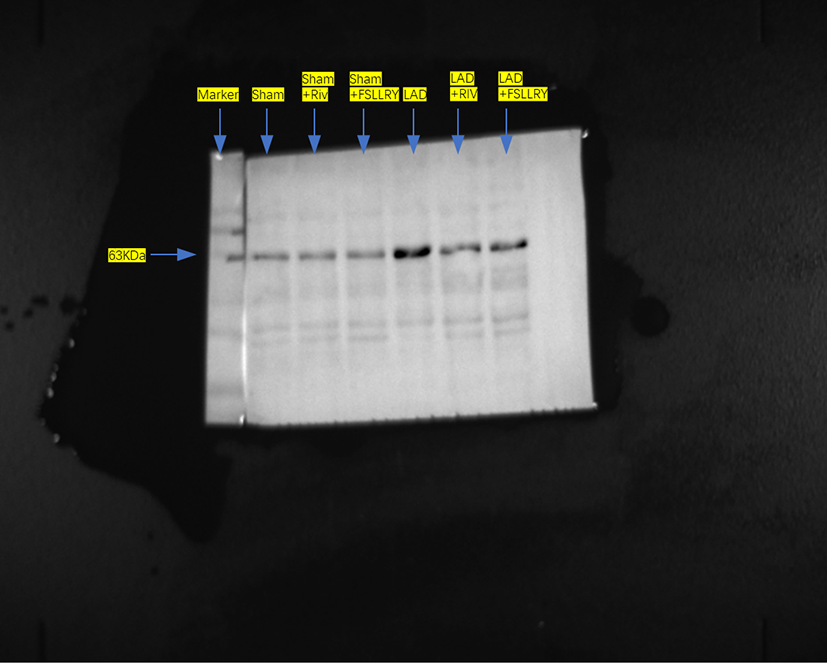

Supplement: Supplemental Information 2 [file peerj-11-16097-s002.zip › Raw data for western blots/raw data for Figure 7B/P-Smad2/C.TIF]

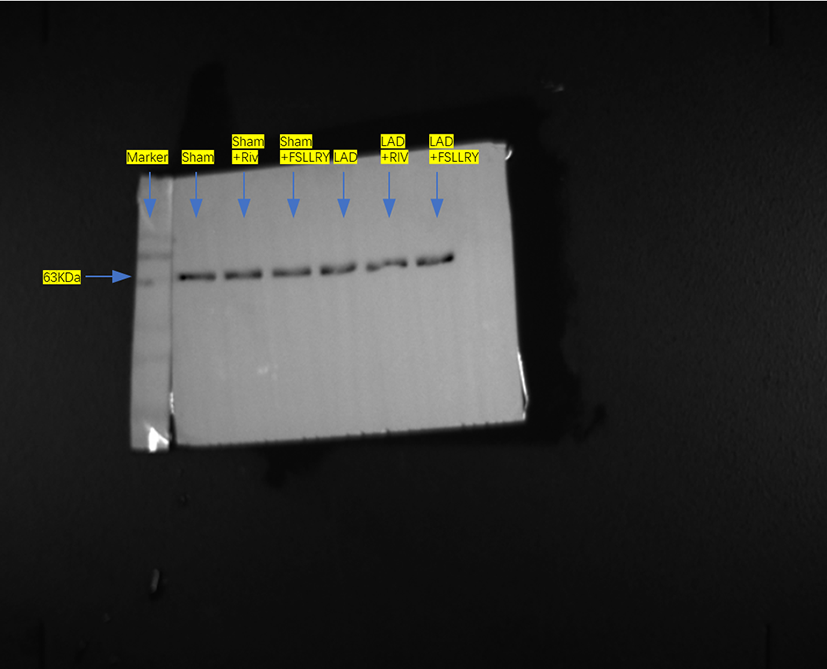

Supplement: Supplemental Information 2 [file peerj-11-16097-s002.zip › Raw data for western blots/raw data for Figure 7B/P-Smad2/C+GAPDH.TIF]

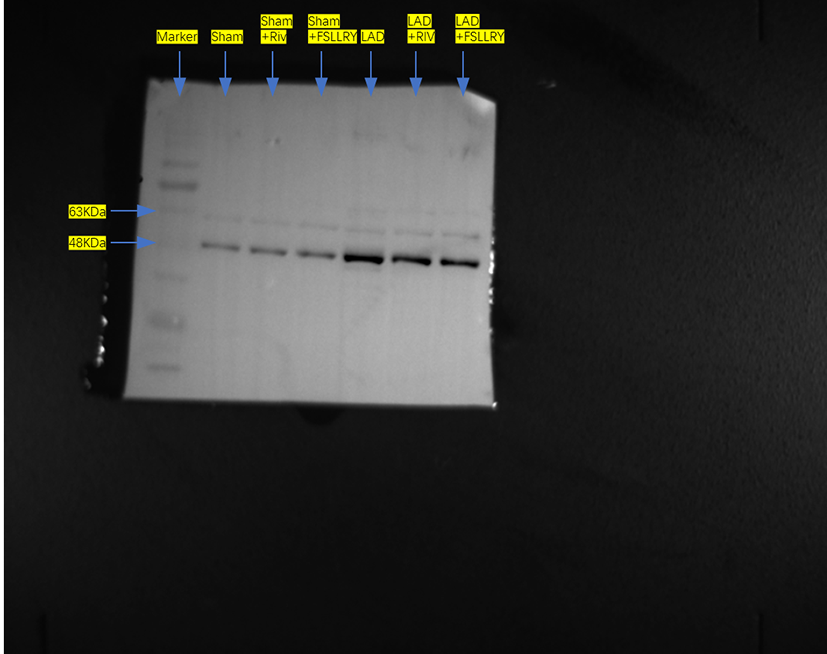

Supplement: Supplemental Information 2 [file peerj-11-16097-s002.zip › Raw data for western blots/raw data for Figure 7B/P-Smad3/A.TIF]

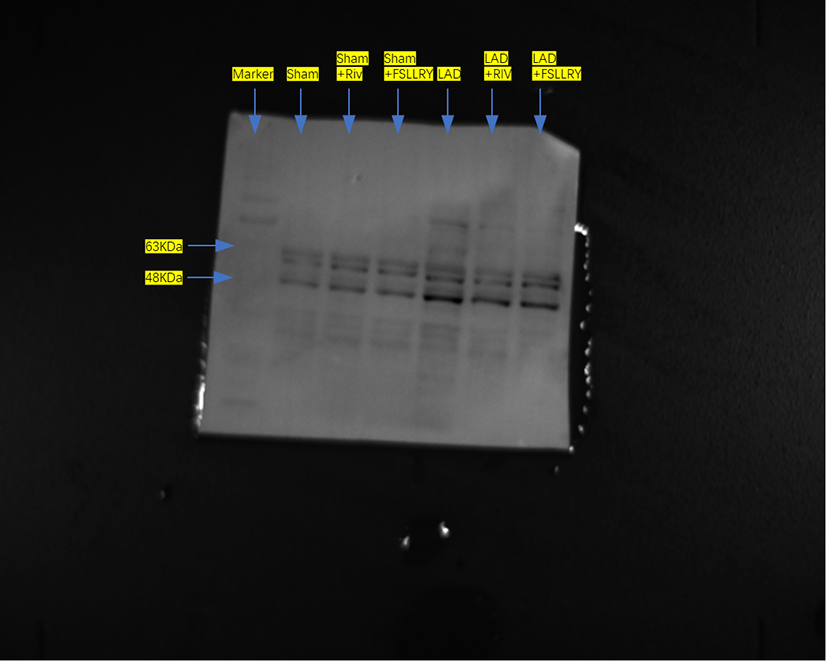

Supplement: Supplemental Information 2 [file peerj-11-16097-s002.zip › Raw data for western blots/raw data for Figure 7B/P-Smad3/A+GAPDH.TIF]

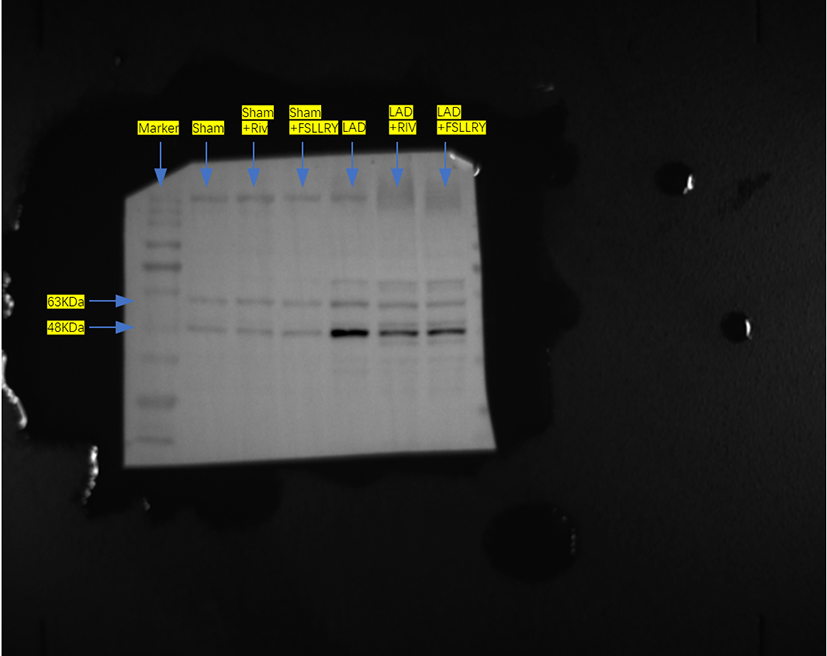

Supplement: Supplemental Information 2 [file peerj-11-16097-s002.zip › Raw data for western blots/raw data for Figure 7B/P-Smad3/B.TIF]

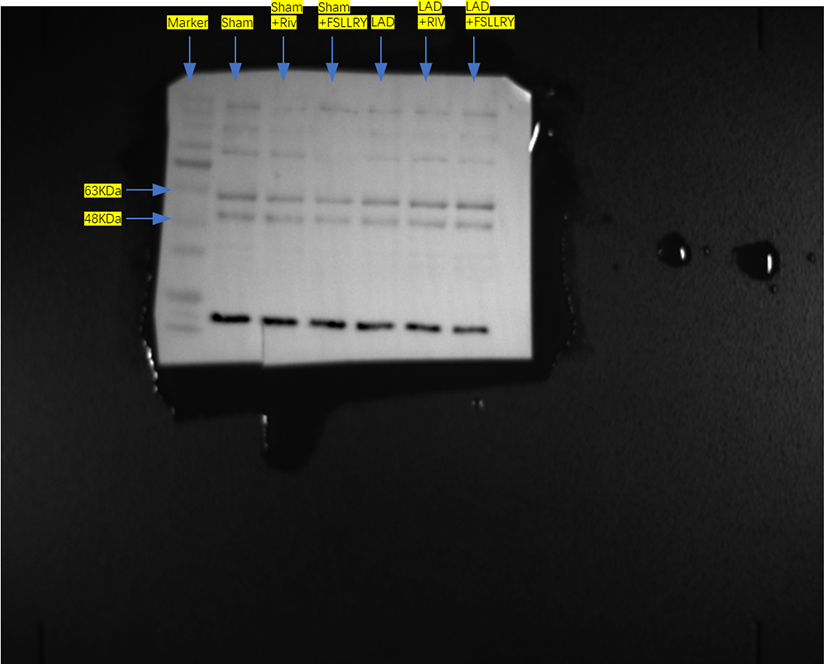

Supplement: Supplemental Information 2 [file peerj-11-16097-s002.zip › Raw data for western blots/raw data for Figure 7B/P-Smad3/B++GAPDH.TIF]

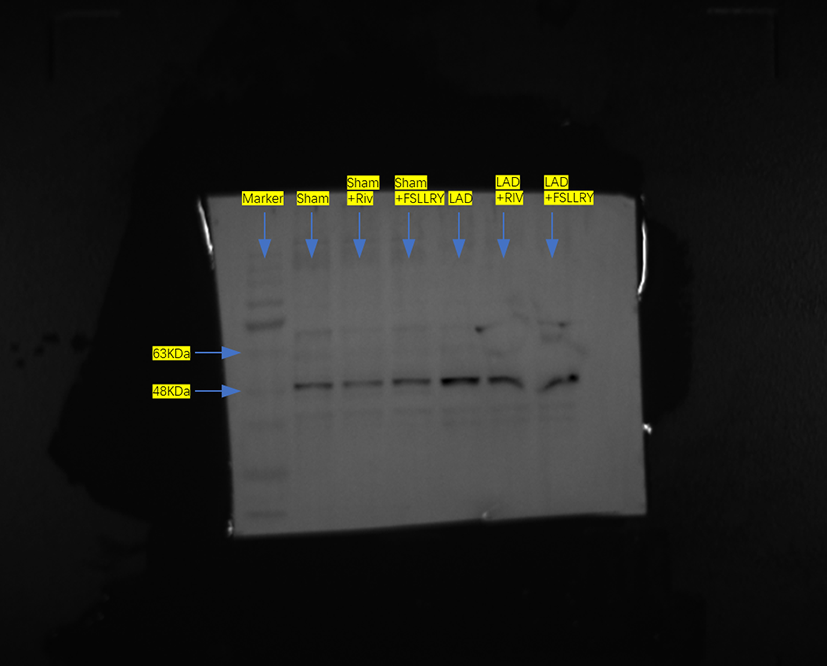

Supplement: Supplemental Information 2 [file peerj-11-16097-s002.zip › Raw data for western blots/raw data for Figure 7B/P-Smad3/C.TIF]

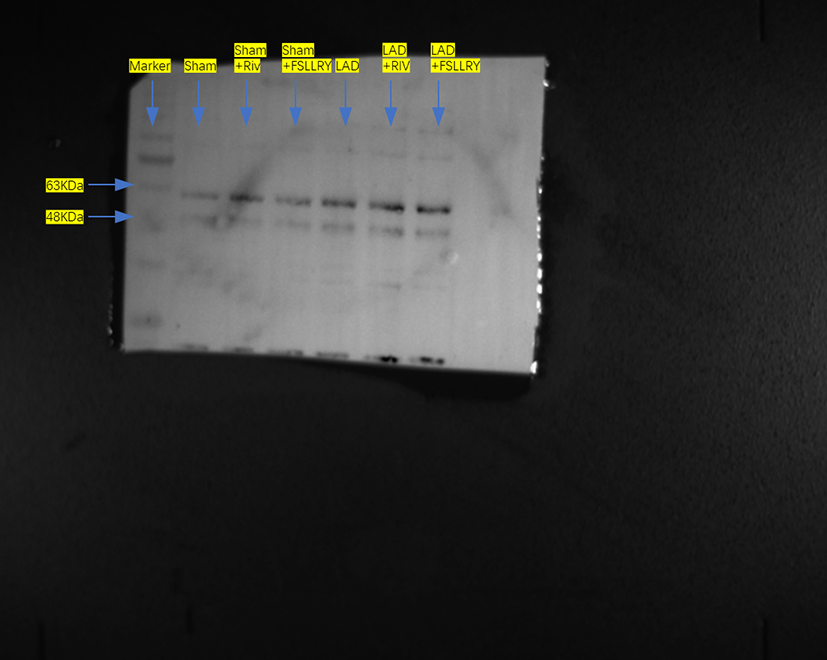

Supplement: Supplemental Information 2 [file peerj-11-16097-s002.zip › Raw data for western blots/raw data for Figure 7B/P-Smad3/C++GAPDH.TIF]

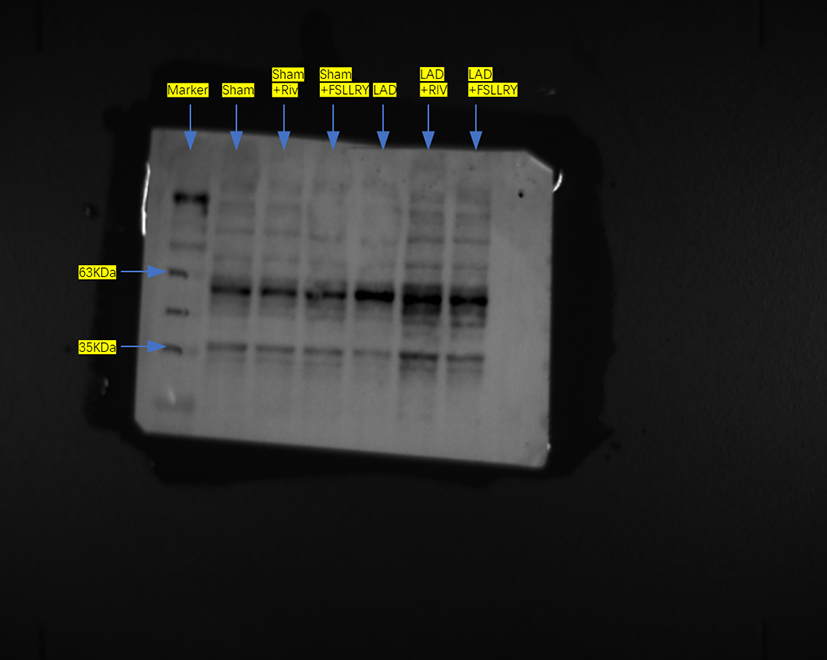

Supplement: Supplemental Information 2 [file peerj-11-16097-s002.zip › Raw data for western blots/raw data for Figure 7B/TGF-a┬/A.TIF]

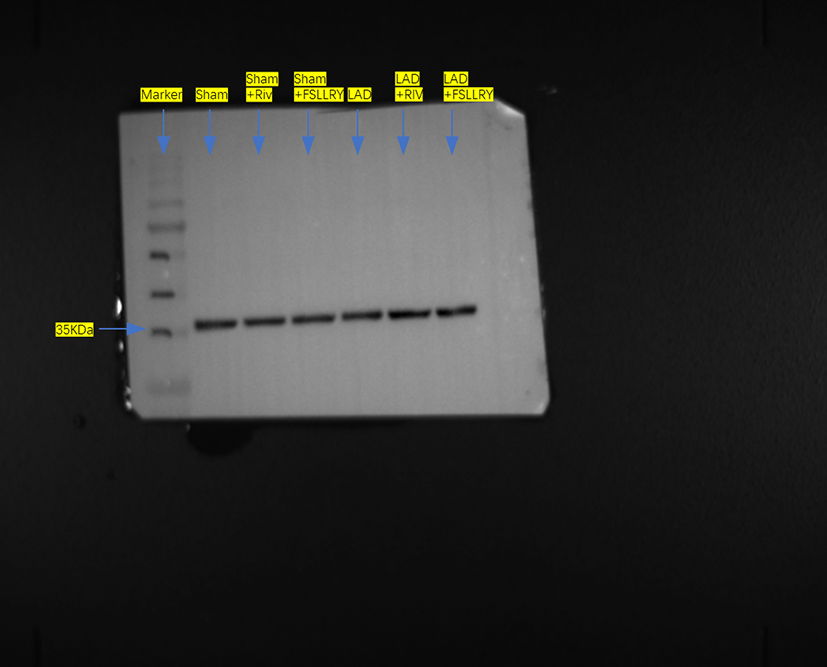

Supplement: Supplemental Information 2 [file peerj-11-16097-s002.zip › Raw data for western blots/raw data for Figure 7B/TGF-a┬/A+GAPDH.TIF]

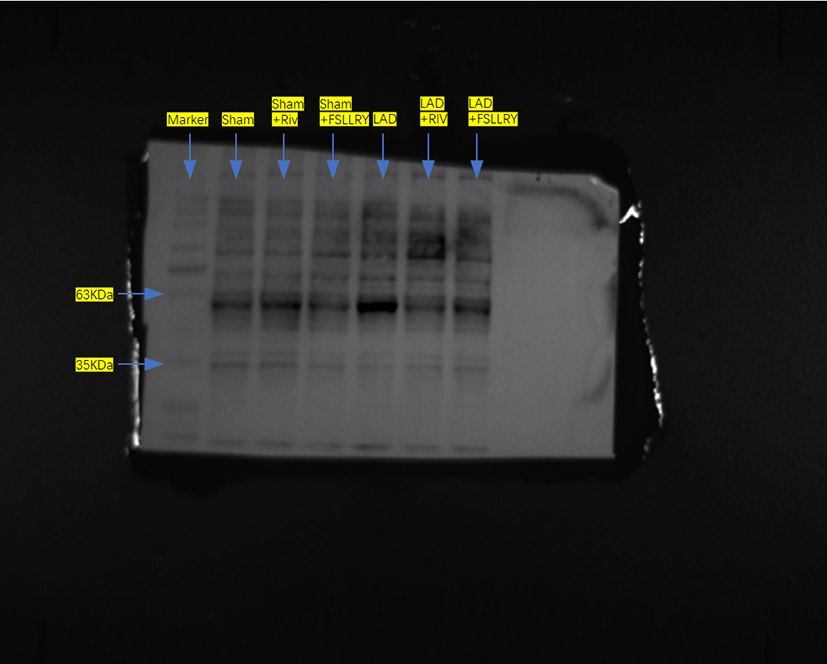

Supplement: Supplemental Information 2 [file peerj-11-16097-s002.zip › Raw data for western blots/raw data for Figure 7B/TGF-a┬/B.TIF]

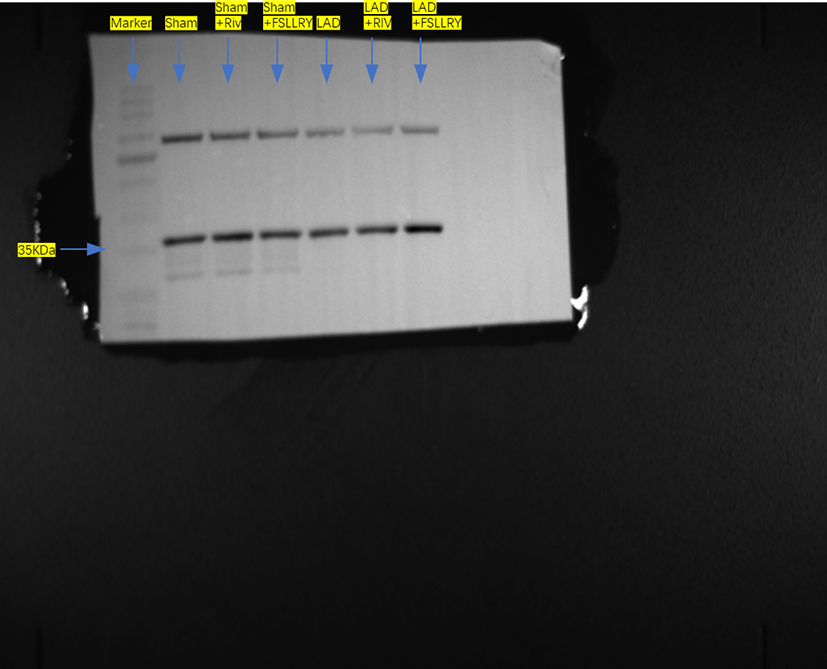

Supplement: Supplemental Information 2 [file peerj-11-16097-s002.zip › Raw data for western blots/raw data for Figure 7B/TGF-a┬/B+GAPDH.TIF]

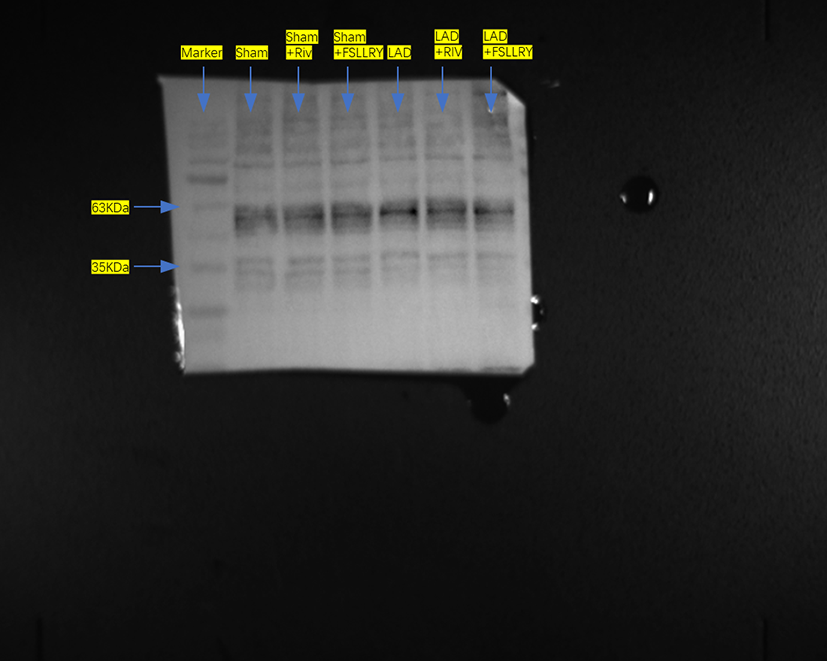

Supplement: Supplemental Information 2 [file peerj-11-16097-s002.zip › Raw data for western blots/raw data for Figure 7B/TGF-a┬/C.TIF]

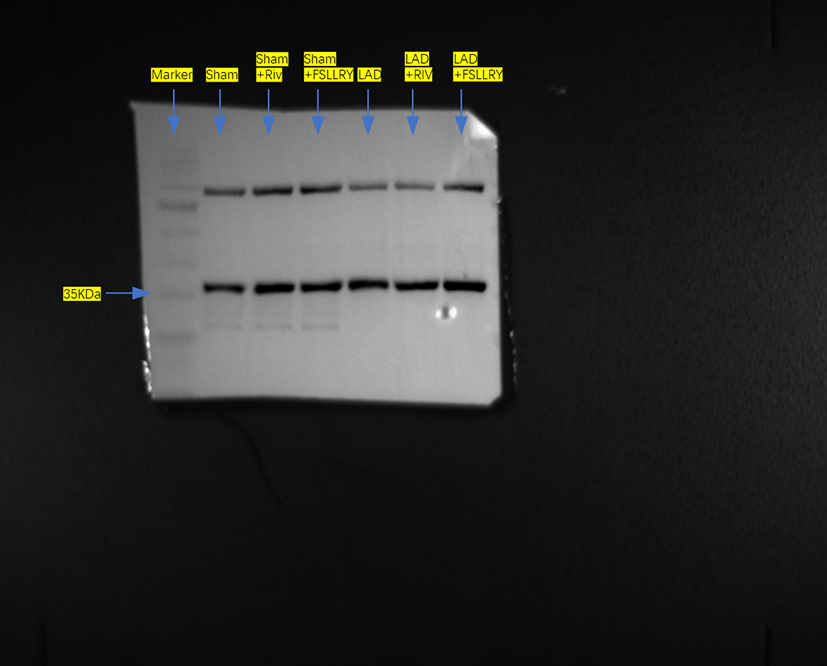

Supplement: Supplemental Information 2 [file peerj-11-16097-s002.zip › Raw data for western blots/raw data for Figure 7B/TGF-a┬/C+GAPDH.TIF]

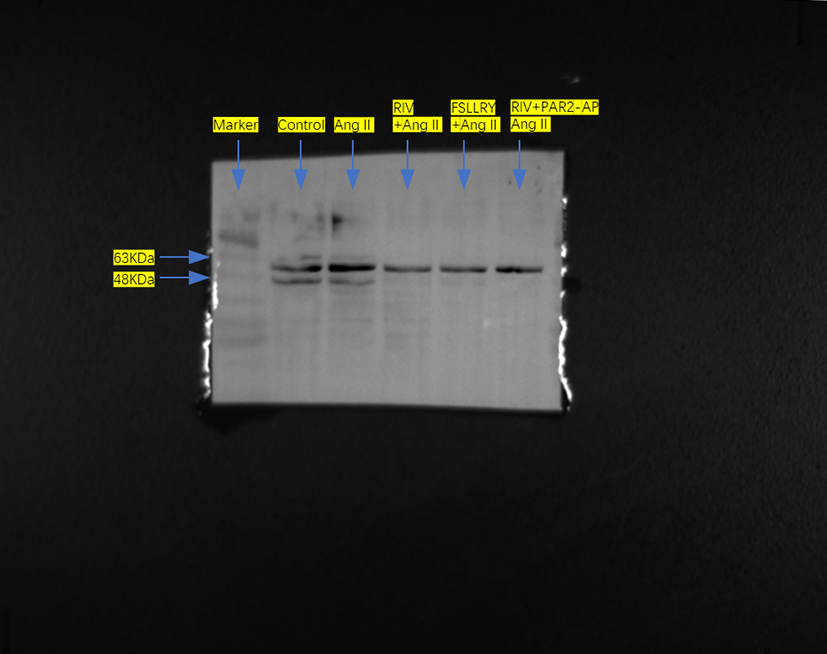

Supplement: Supplemental Information 2 [file peerj-11-16097-s002.zip › Raw data for western blots/raw data for Figure 8A/PAR2/A.TIF]

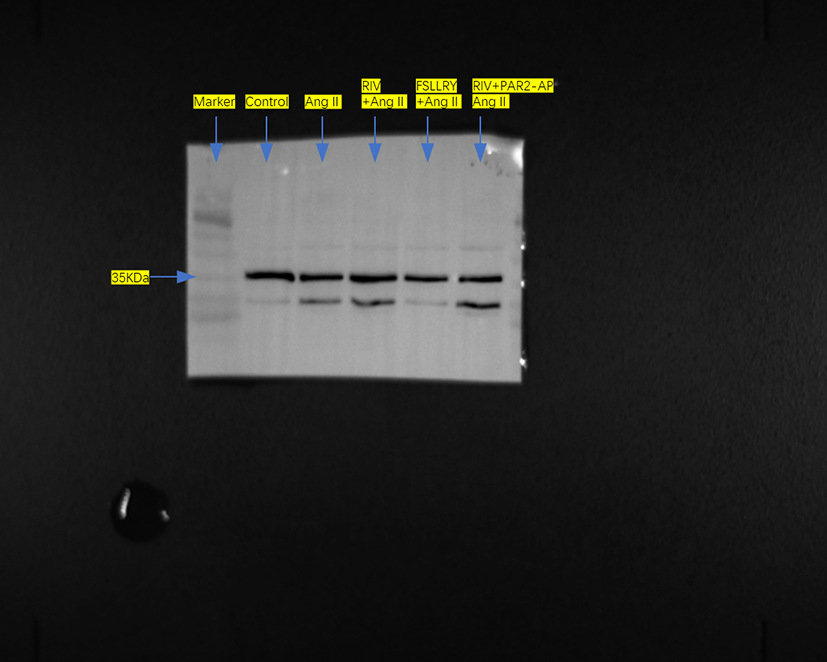

Supplement: Supplemental Information 2 [file peerj-11-16097-s002.zip › Raw data for western blots/raw data for Figure 8A/PAR2/A+GAPDH.TIF]

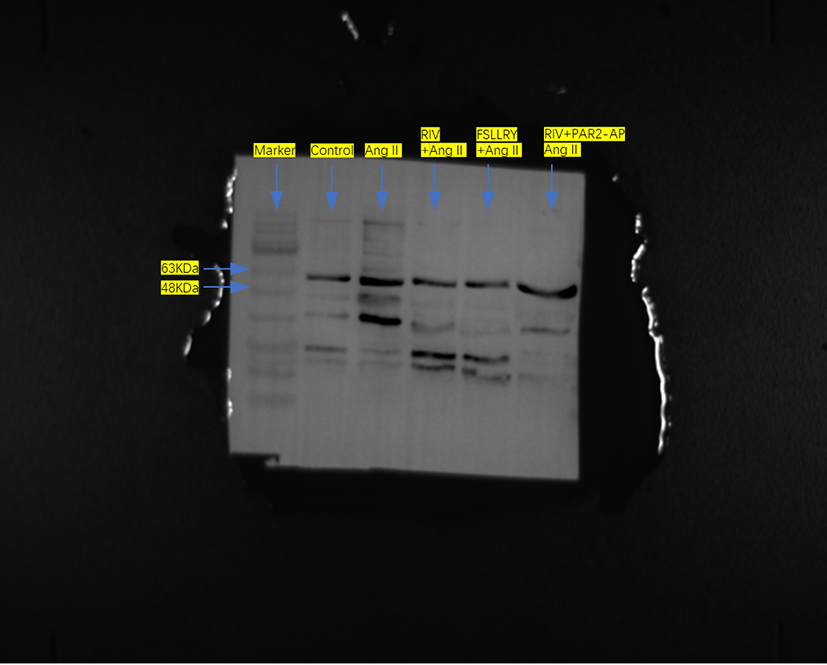

Supplement: Supplemental Information 2 [file peerj-11-16097-s002.zip › Raw data for western blots/raw data for Figure 8A/PAR2/B.TIF]

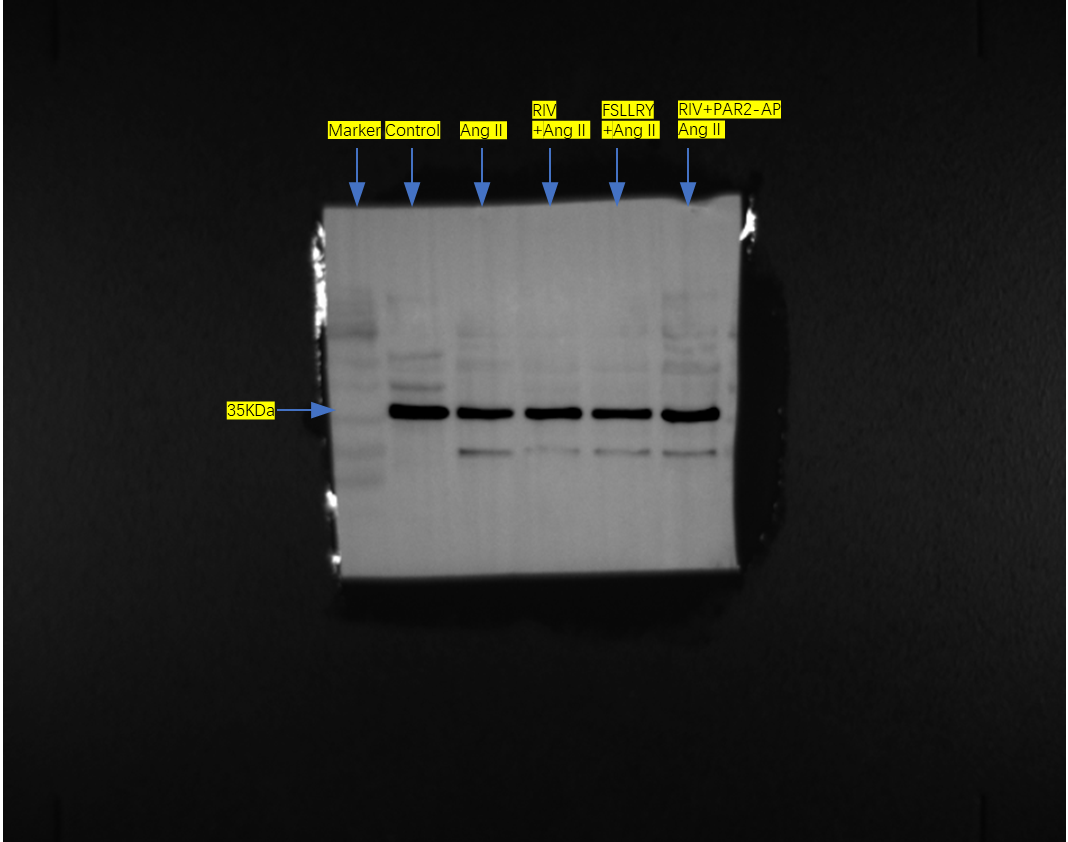

Supplement: Supplemental Information 2 [file peerj-11-16097-s002.zip › Raw data for western blots/raw data for Figure 8A/PAR2/B+GAPDH.TIF]

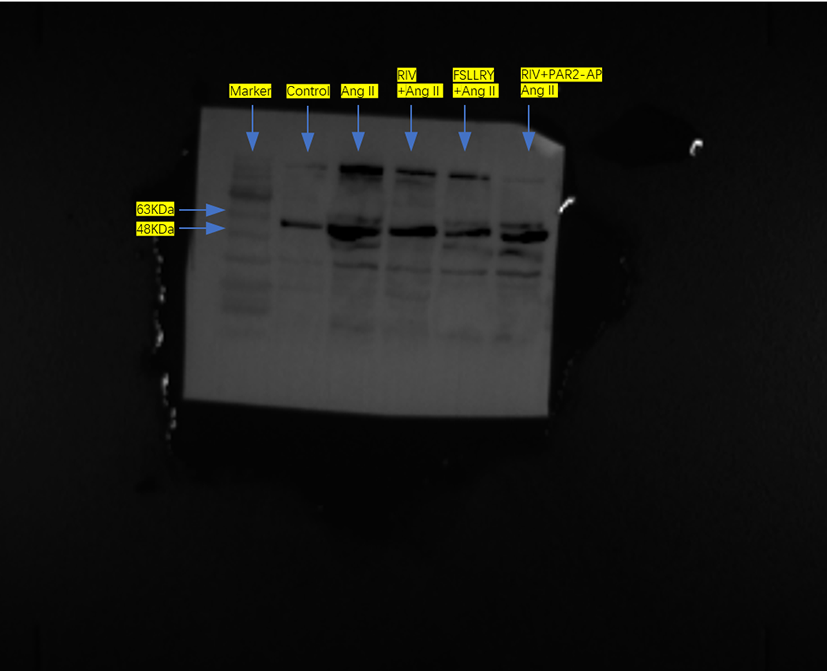

Supplement: Supplemental Information 2 [file peerj-11-16097-s002.zip › Raw data for western blots/raw data for Figure 8A/PAR2/C.TIF]

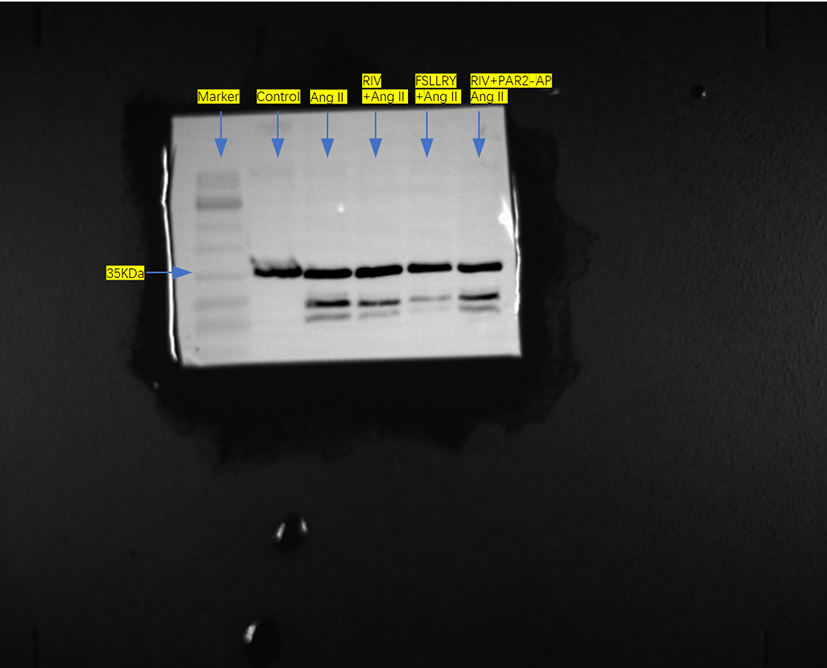

Supplement: Supplemental Information 2 [file peerj-11-16097-s002.zip › Raw data for western blots/raw data for Figure 8A/PAR2/C+GAPDH.TIF]

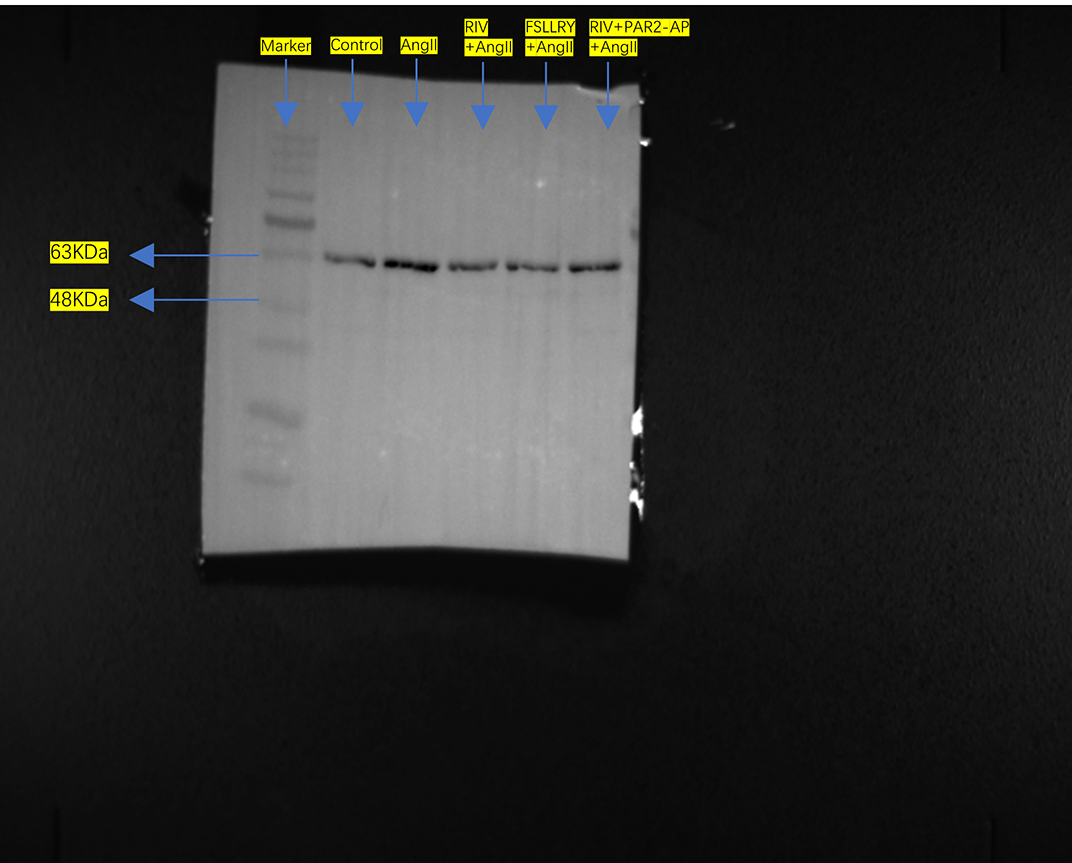

Supplement: Supplemental Information 2 [file peerj-11-16097-s002.zip › Raw data for western blots/raw data for Figure 8A/P-Smad2/A.TIF]

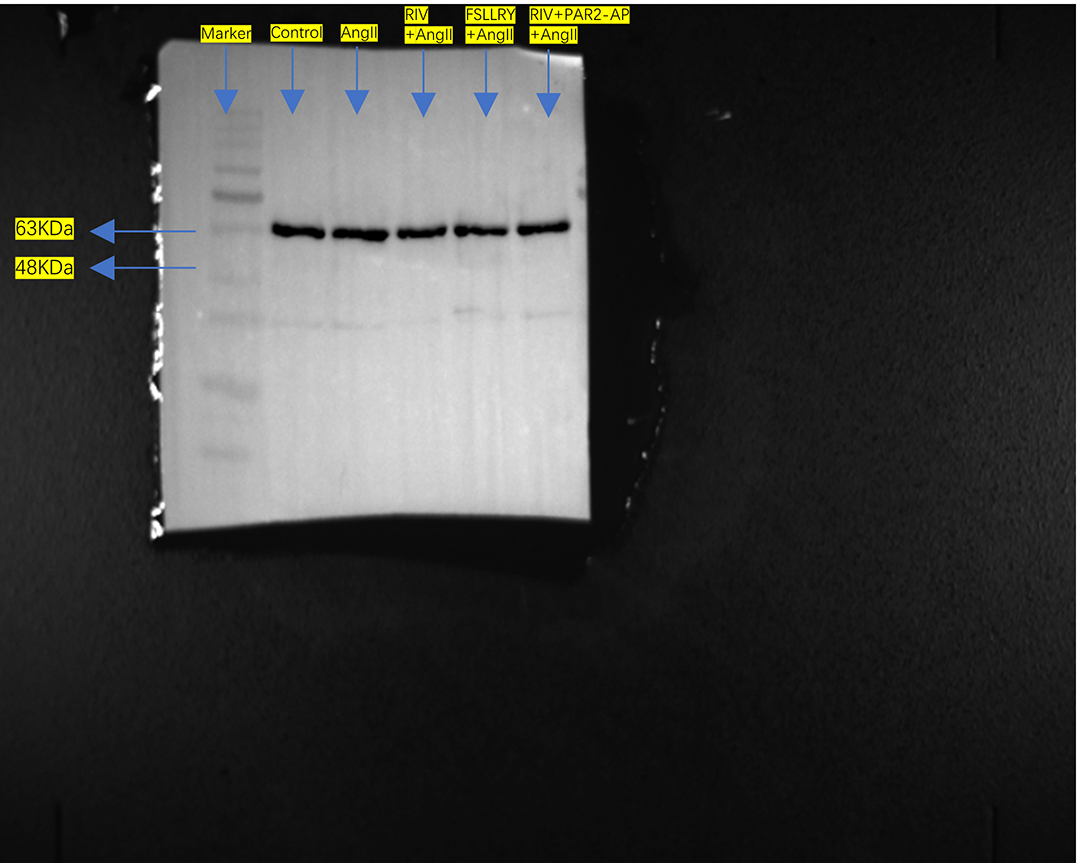

Supplement: Supplemental Information 2 [file peerj-11-16097-s002.zip › Raw data for western blots/raw data for Figure 8A/P-Smad2/A+Smad2.TIF]

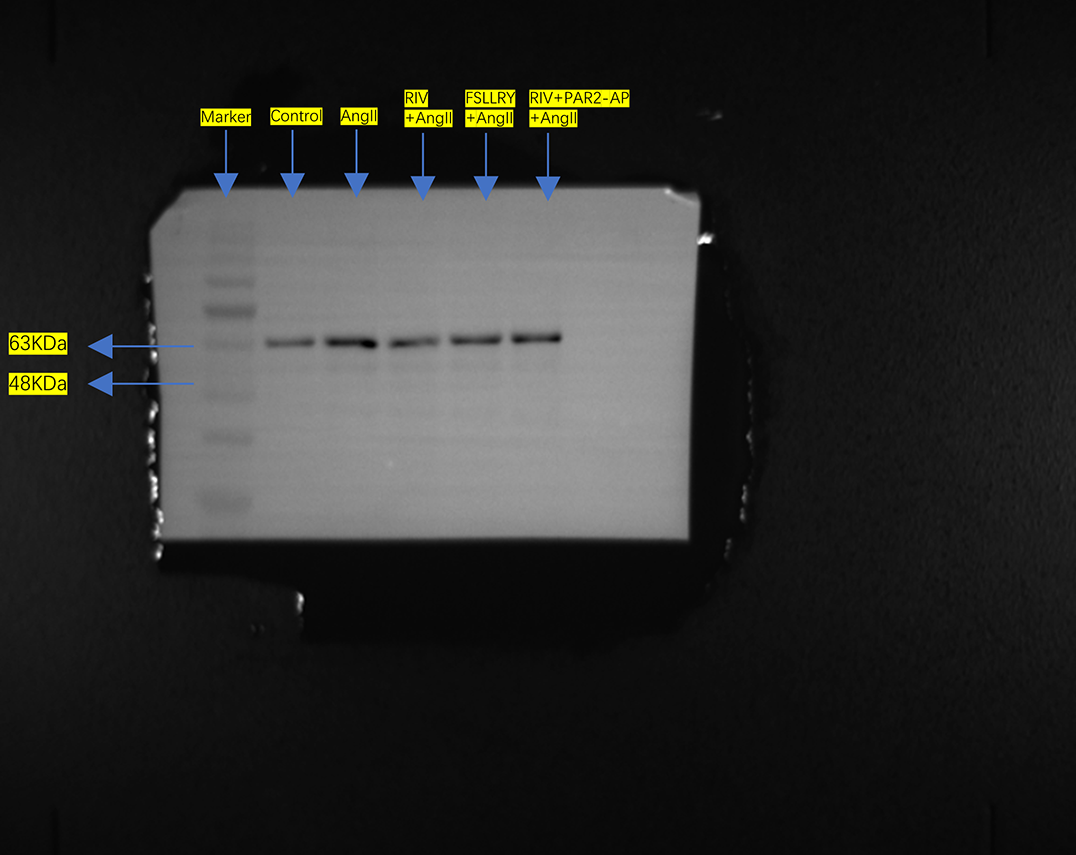

Supplement: Supplemental Information 2 [file peerj-11-16097-s002.zip › Raw data for western blots/raw data for Figure 8A/P-Smad2/B.TIF]

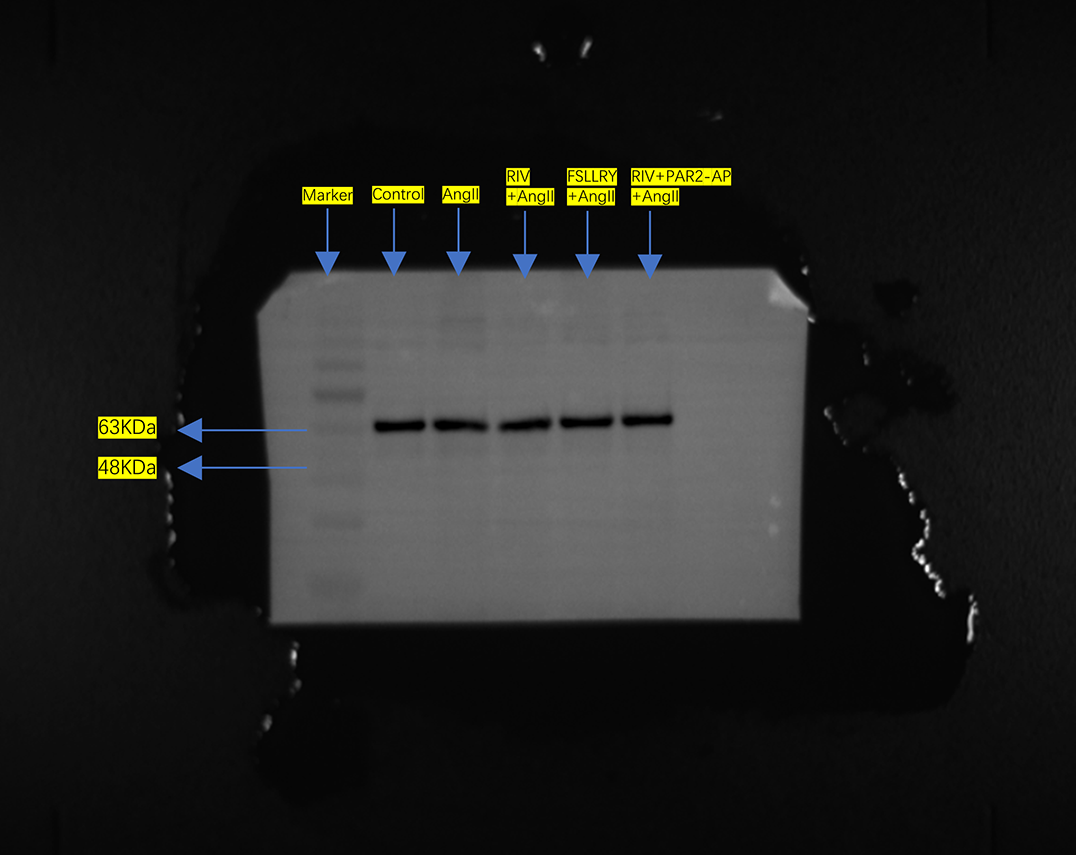

Supplement: Supplemental Information 2 [file peerj-11-16097-s002.zip › Raw data for western blots/raw data for Figure 8A/P-Smad2/B+Smad2.TIF]

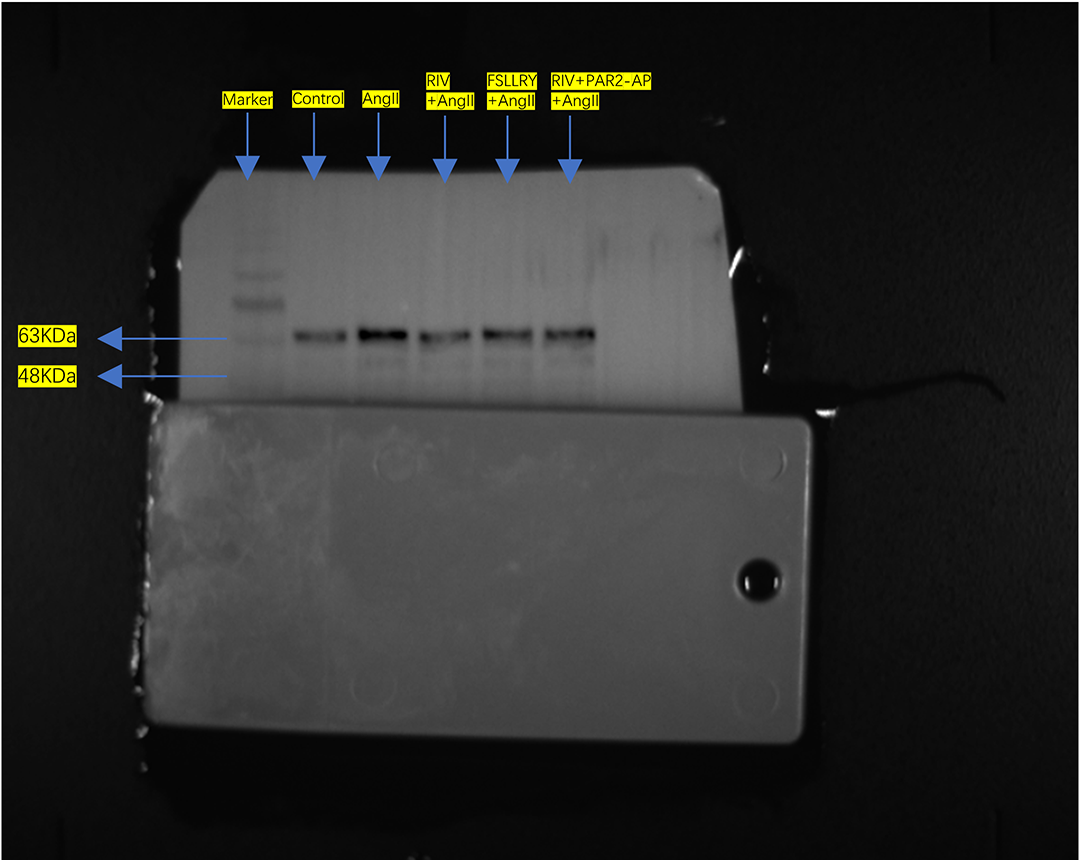

Supplement: Supplemental Information 2 [file peerj-11-16097-s002.zip › Raw data for western blots/raw data for Figure 8A/P-Smad2/C.TIF]

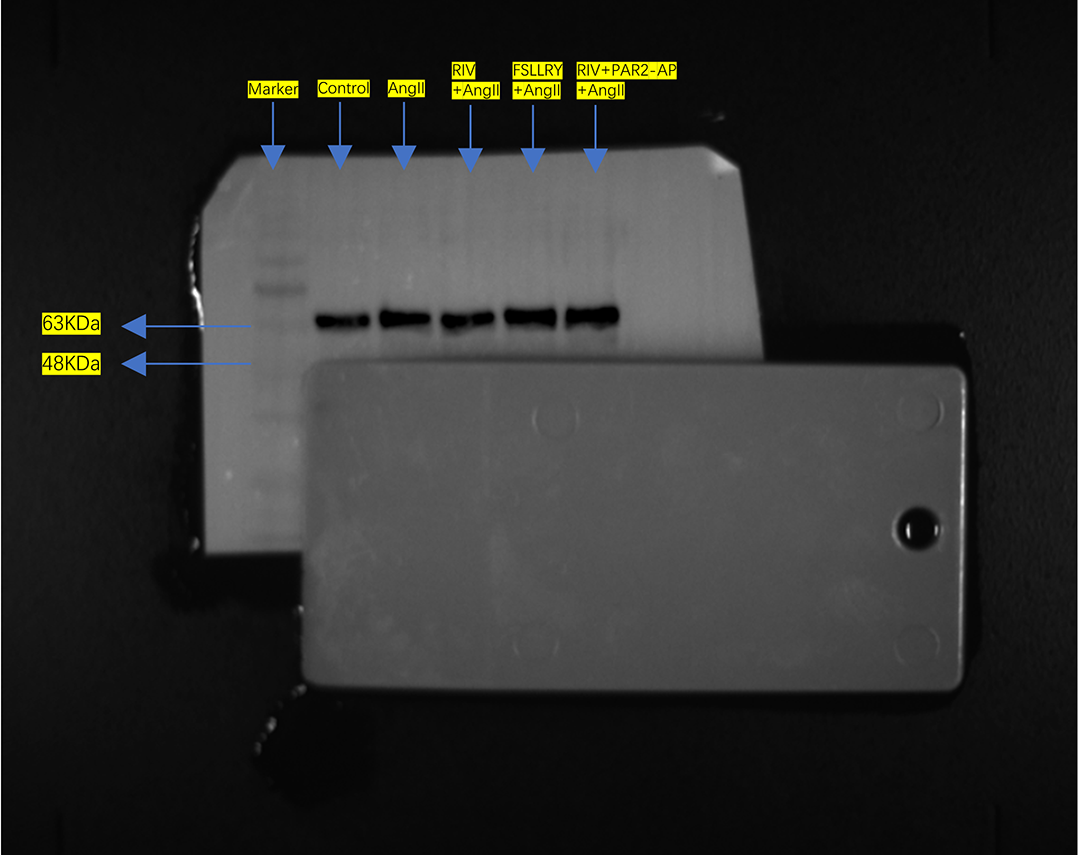

Supplement: Supplemental Information 2 [file peerj-11-16097-s002.zip › Raw data for western blots/raw data for Figure 8A/P-Smad2/C+Smad2.TIF]

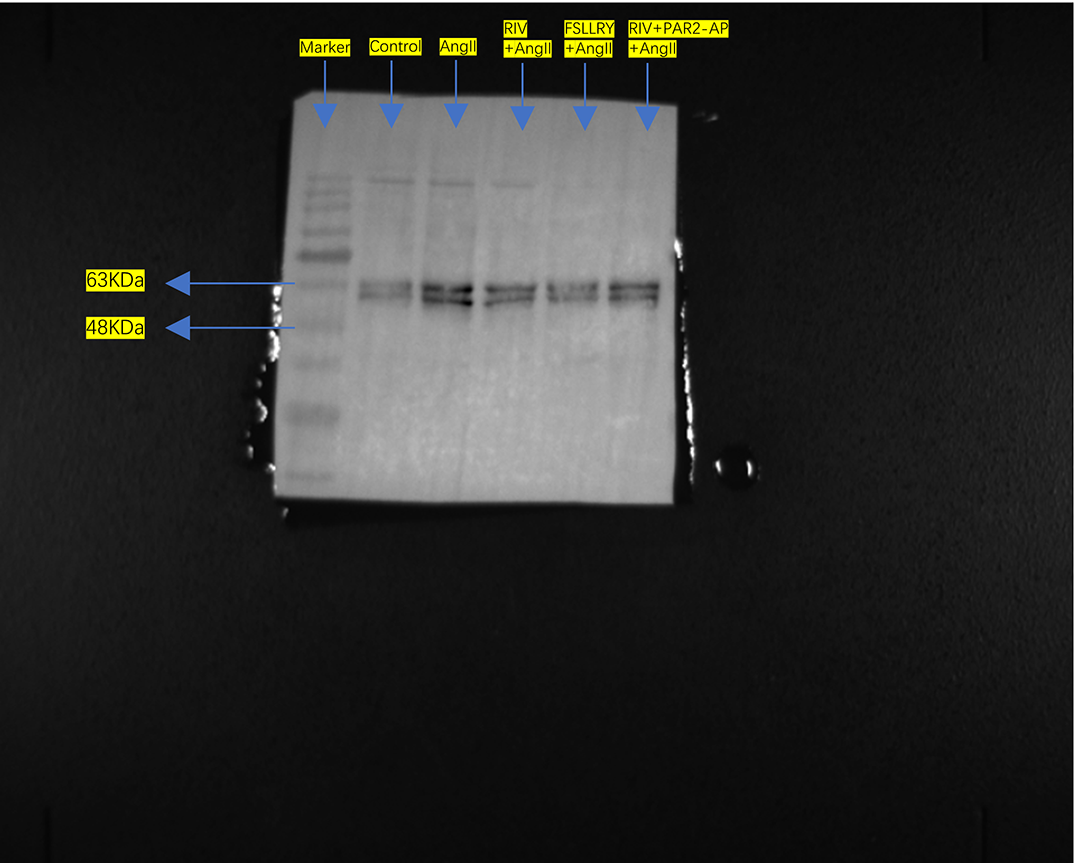

Supplement: Supplemental Information 2 [file peerj-11-16097-s002.zip › Raw data for western blots/raw data for Figure 8A/P-Smad3/A.TIF]

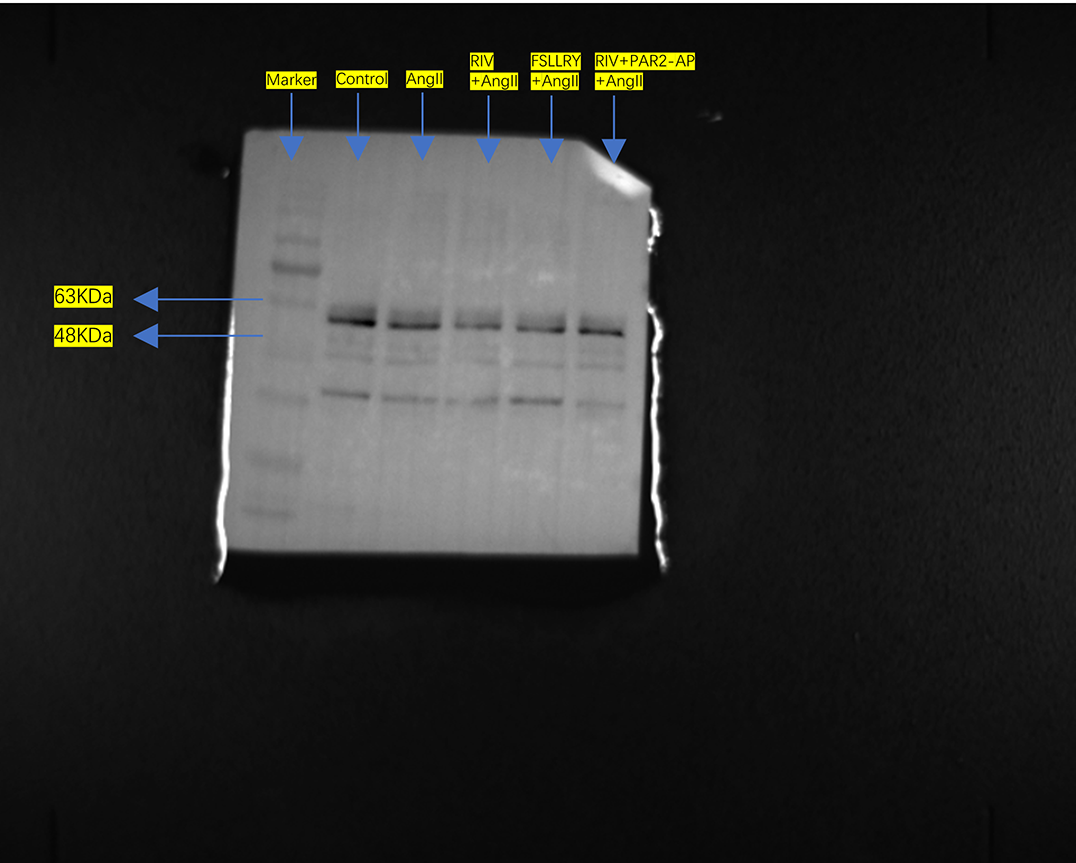

Supplement: Supplemental Information 2 [file peerj-11-16097-s002.zip › Raw data for western blots/raw data for Figure 8A/P-Smad3/A+Smad3.TIF]

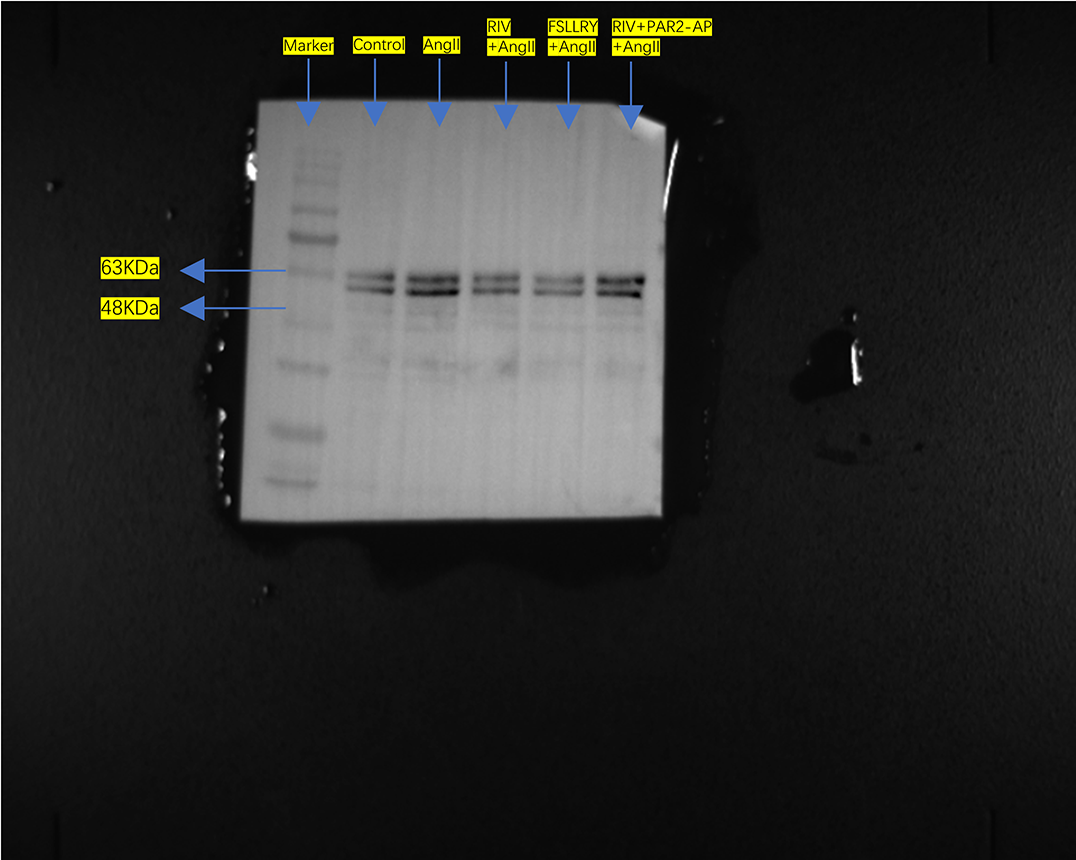

Supplement: Supplemental Information 2 [file peerj-11-16097-s002.zip › Raw data for western blots/raw data for Figure 8A/P-Smad3/B.TIF]

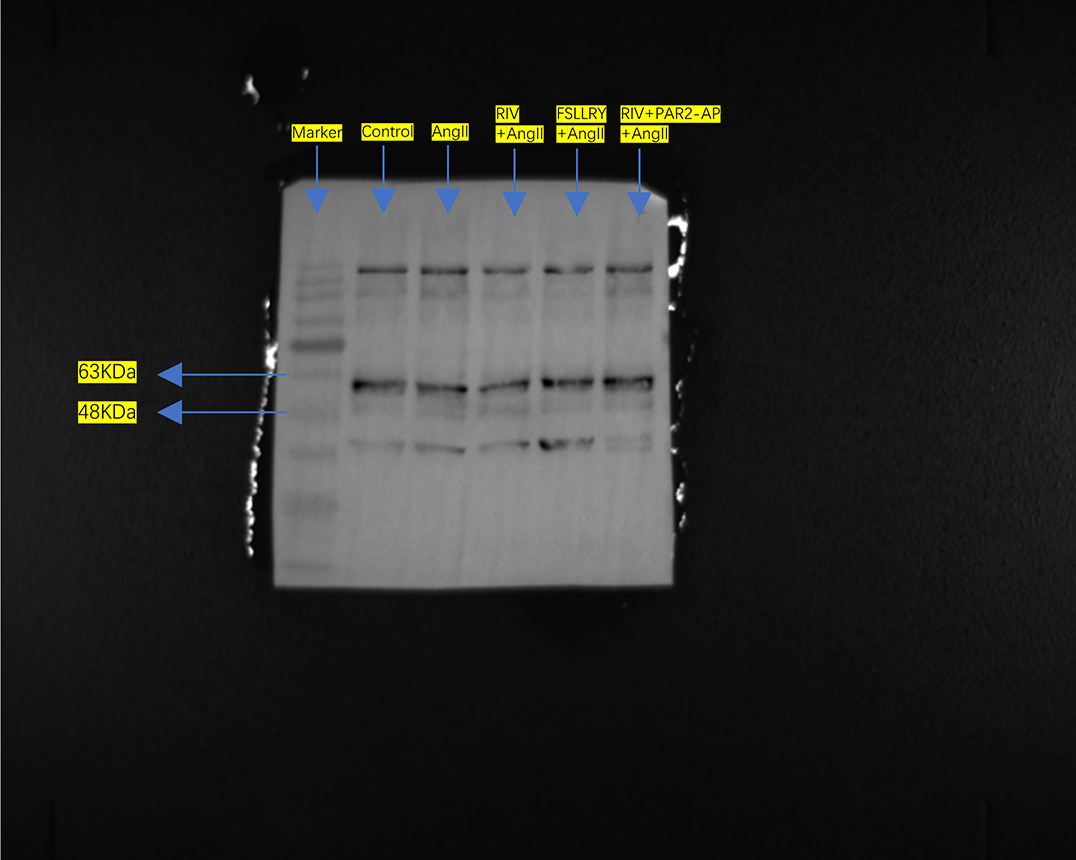

Supplement: Supplemental Information 2 [file peerj-11-16097-s002.zip › Raw data for western blots/raw data for Figure 8A/P-Smad3/B+Smad3.TIF]

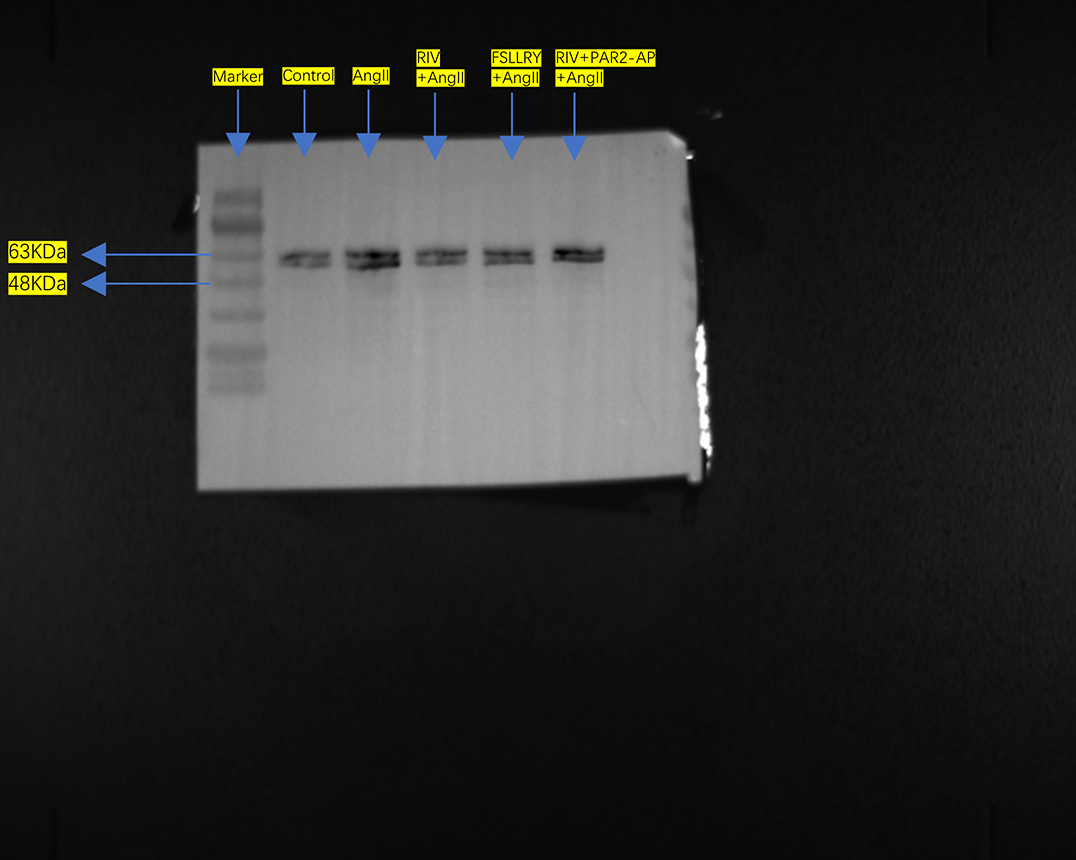

Supplement: Supplemental Information 2 [file peerj-11-16097-s002.zip › Raw data for western blots/raw data for Figure 8A/P-Smad3/C.TIF]

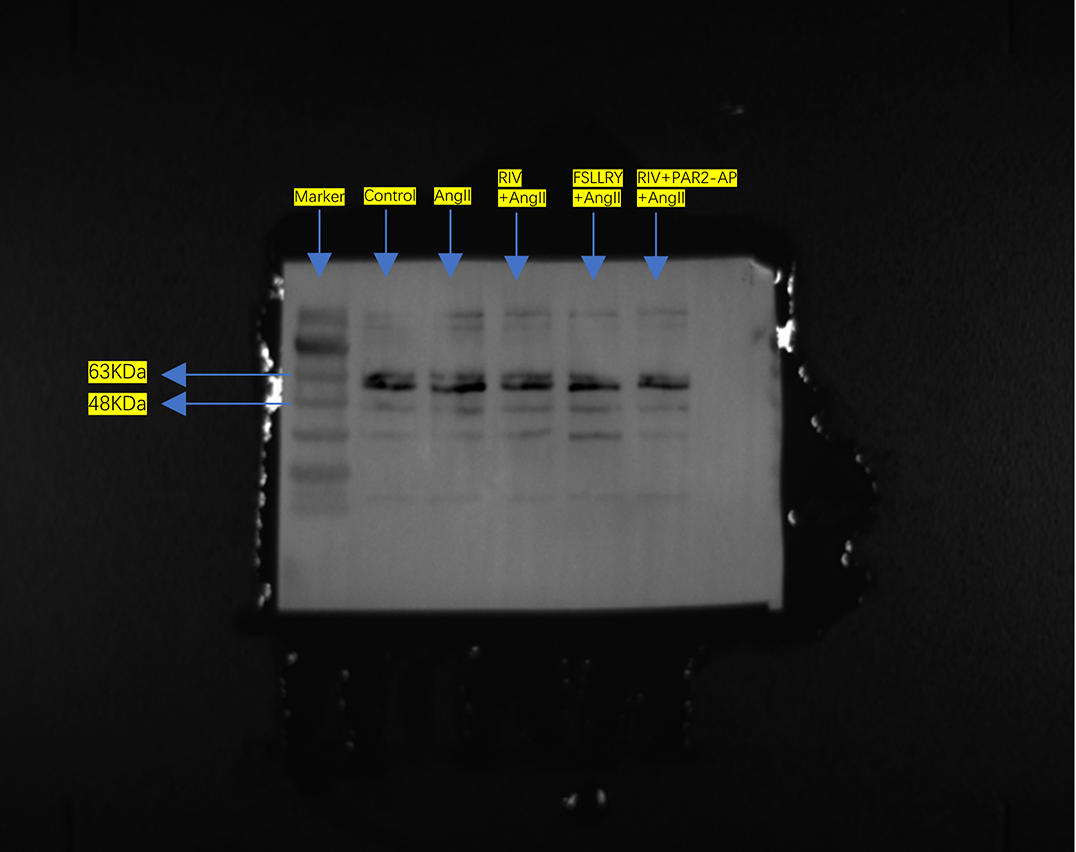

Supplement: Supplemental Information 2 [file peerj-11-16097-s002.zip › Raw data for western blots/raw data for Figure 8A/P-Smad3/C+Smad3.TIF]

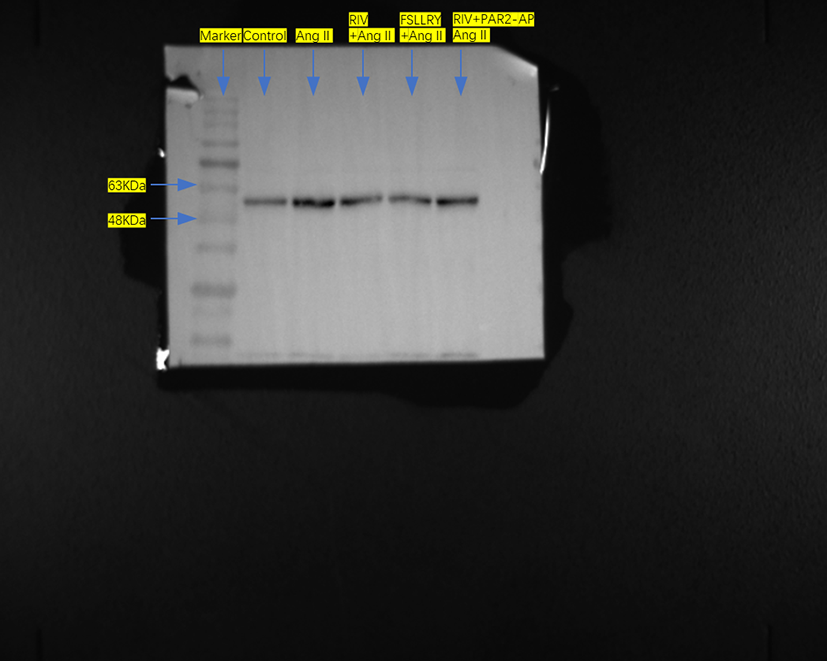

Supplement: Supplemental Information 2 [file peerj-11-16097-s002.zip › Raw data for western blots/raw data for Figure 8A/TGF/A.TIF]

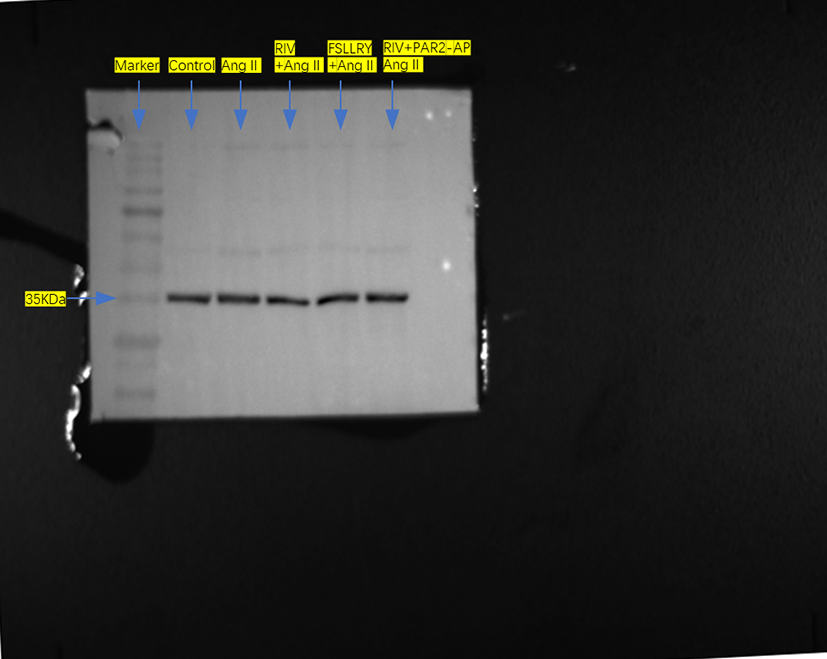

Supplement: Supplemental Information 2 [file peerj-11-16097-s002.zip › Raw data for western blots/raw data for Figure 8A/TGF/A+GAPDH.TIF]

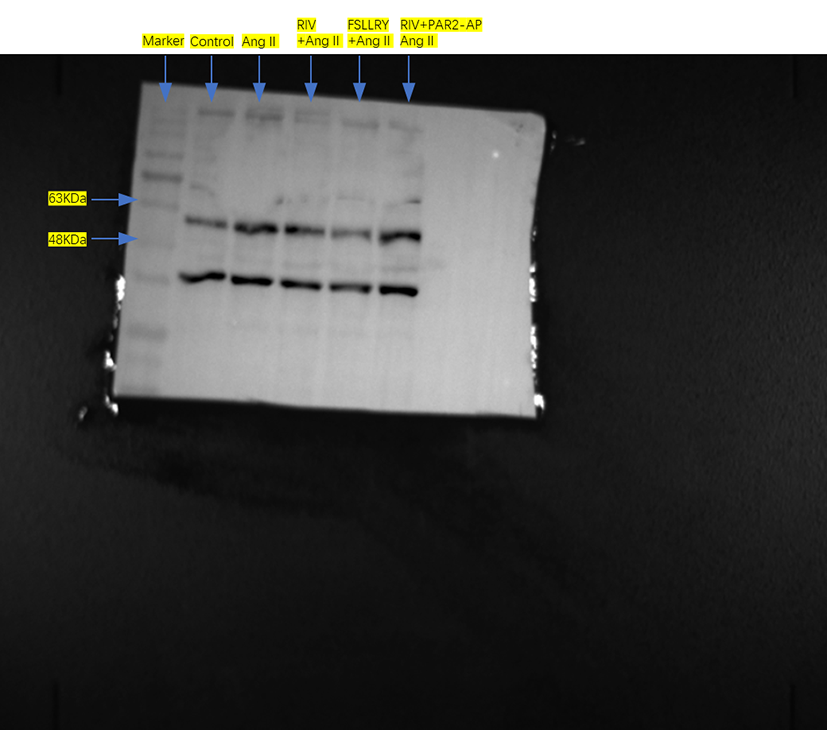

Supplement: Supplemental Information 2 [file peerj-11-16097-s002.zip › Raw data for western blots/raw data for Figure 8A/TGF/B.TIF]

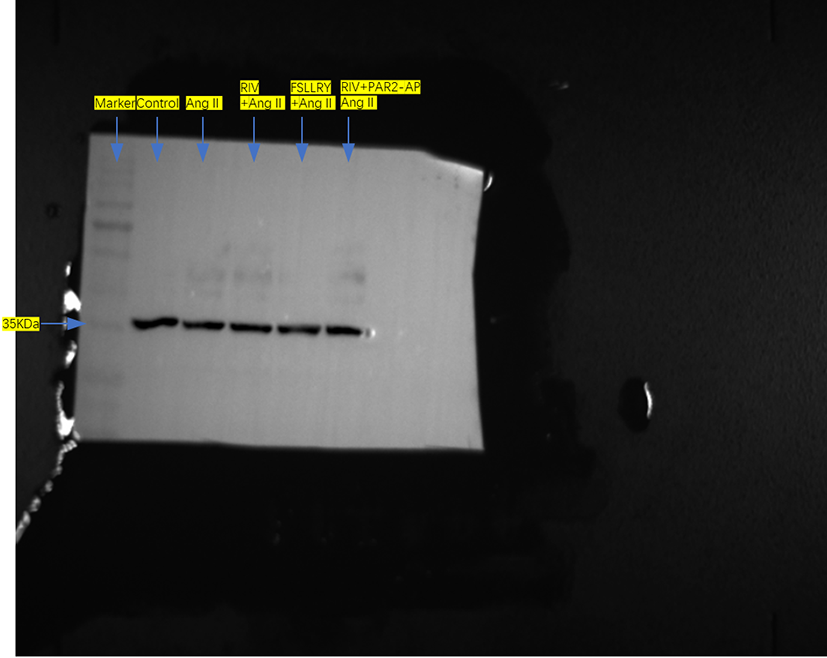

Supplement: Supplemental Information 2 [file peerj-11-16097-s002.zip › Raw data for western blots/raw data for Figure 8A/TGF/B+GAPDH.TIF]

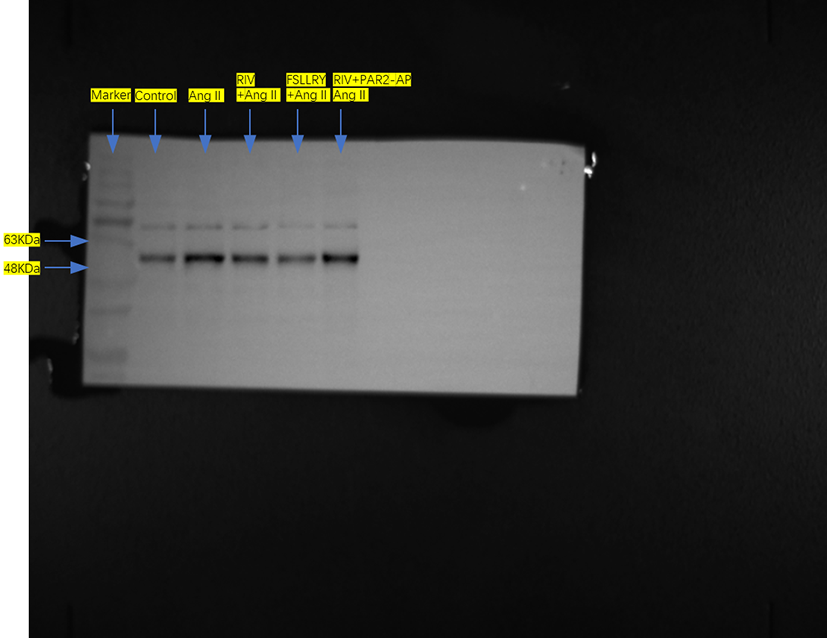

Supplement: Supplemental Information 2 [file peerj-11-16097-s002.zip › Raw data for western blots/raw data for Figure 8A/TGF/C.TIF]

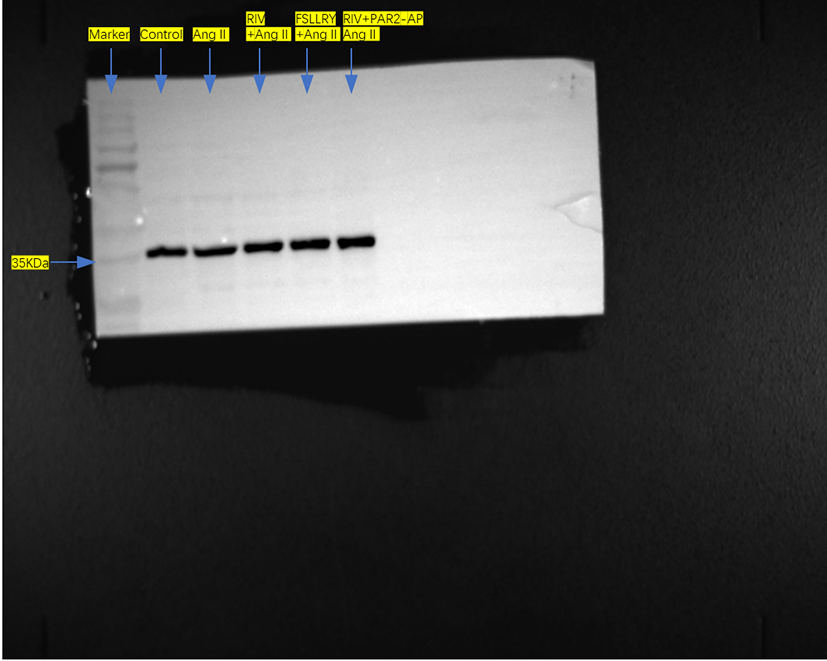

Supplement: Supplemental Information 2 [file peerj-11-16097-s002.zip › Raw data for western blots/raw data for Figure 8A/TGF/C+GAPDH.TIF]

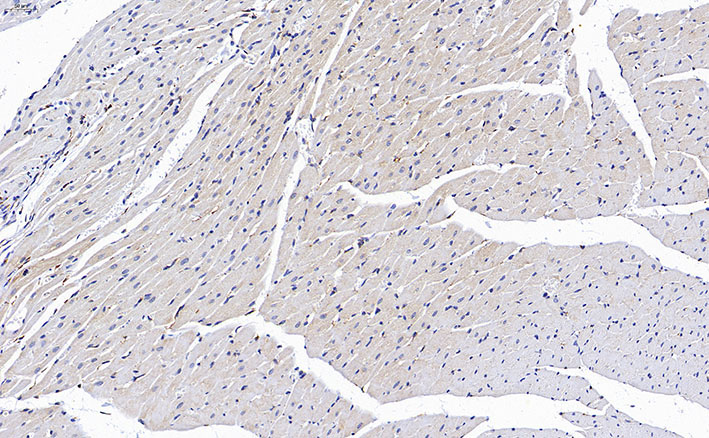

Supplement: Supplemental Information 3 [file peerj-11-16097-s003.zip › Raw data for IHC images/PAR2/LAD/A.jpg]

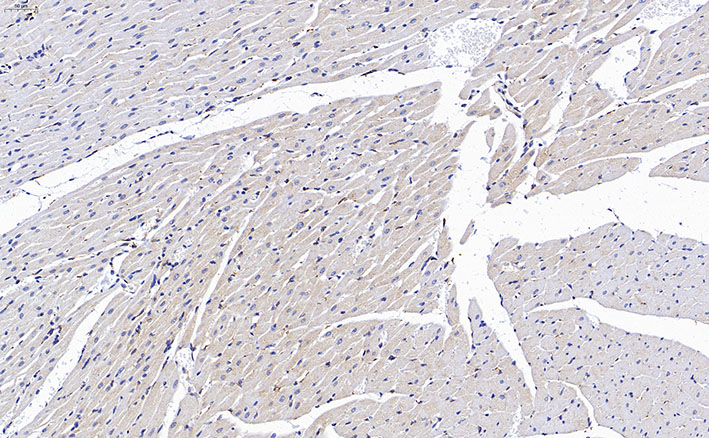

Supplement: Supplemental Information 3 [file peerj-11-16097-s003.zip › Raw data for IHC images/PAR2/LAD/B.jpg]

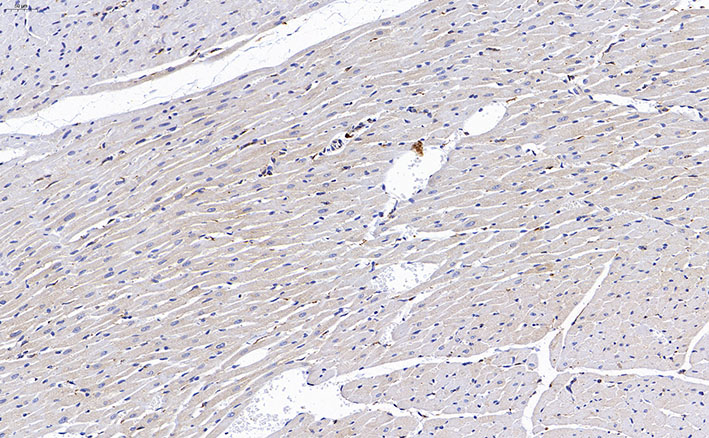

Supplement: Supplemental Information 3 [file peerj-11-16097-s003.zip › Raw data for IHC images/PAR2/LAD/C.jpg]

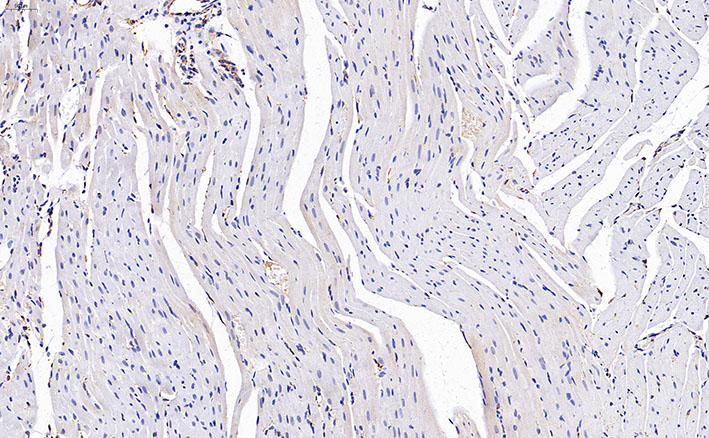

Supplement: Supplemental Information 3 [file peerj-11-16097-s003.zip › Raw data for IHC images/PAR2/LAD+FSLLRY/A.jpg]

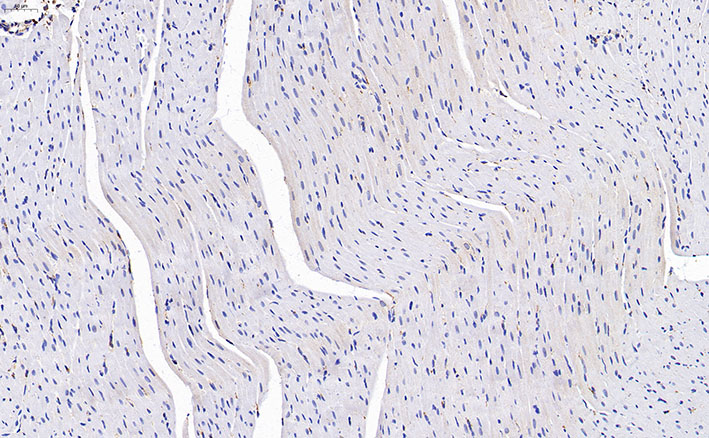

Supplement: Supplemental Information 3 [file peerj-11-16097-s003.zip › Raw data for IHC images/PAR2/LAD+FSLLRY/B.jpg]

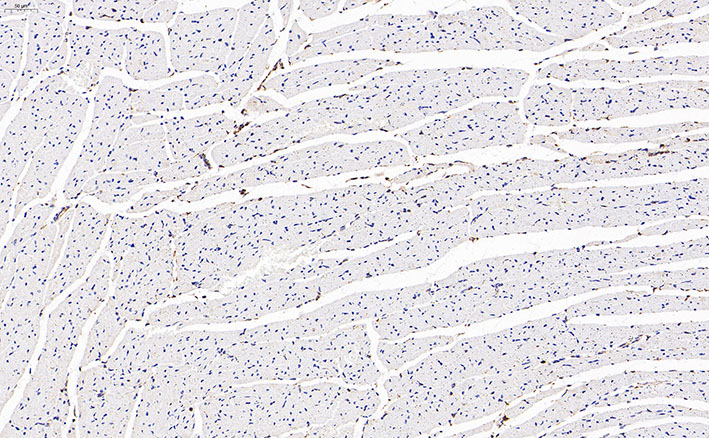

Supplement: Supplemental Information 3 [file peerj-11-16097-s003.zip › Raw data for IHC images/PAR2/LAD+FSLLRY/C.jpg]

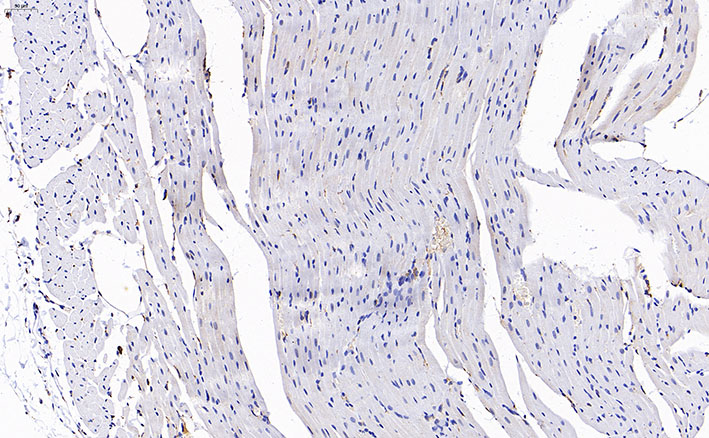

Supplement: Supplemental Information 3 [file peerj-11-16097-s003.zip › Raw data for IHC images/PAR2/LAD+RIV/A.jpg]

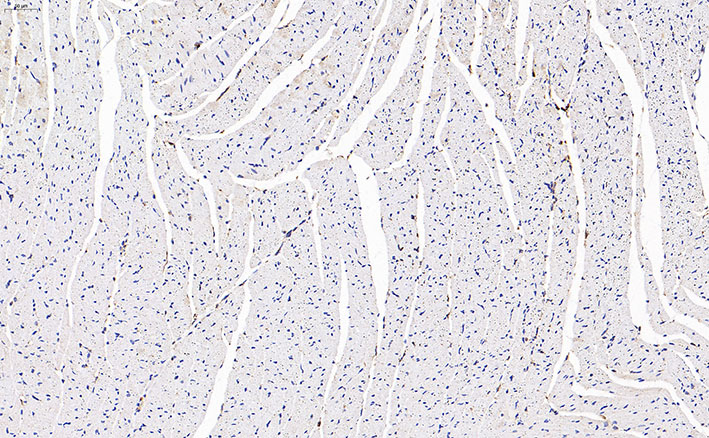

Supplement: Supplemental Information 3 [file peerj-11-16097-s003.zip › Raw data for IHC images/PAR2/LAD+RIV/B.jpg]

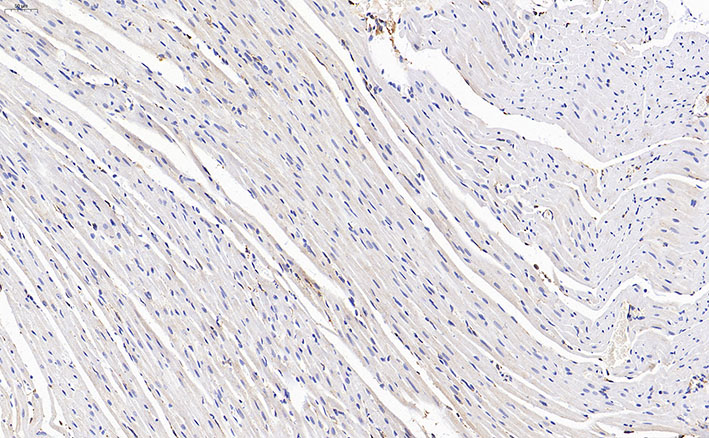

Supplement: Supplemental Information 3 [file peerj-11-16097-s003.zip › Raw data for IHC images/PAR2/LAD+RIV/C.jpg]

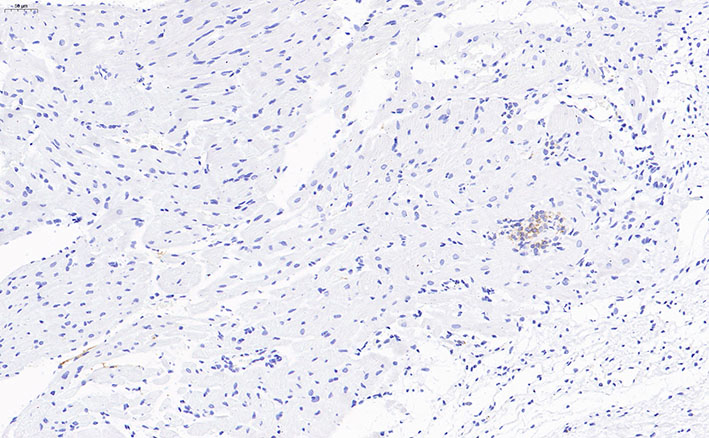

Supplement: Supplemental Information 3 [file peerj-11-16097-s003.zip › Raw data for IHC images/PAR2/Negative control/LAD/A.jpg]

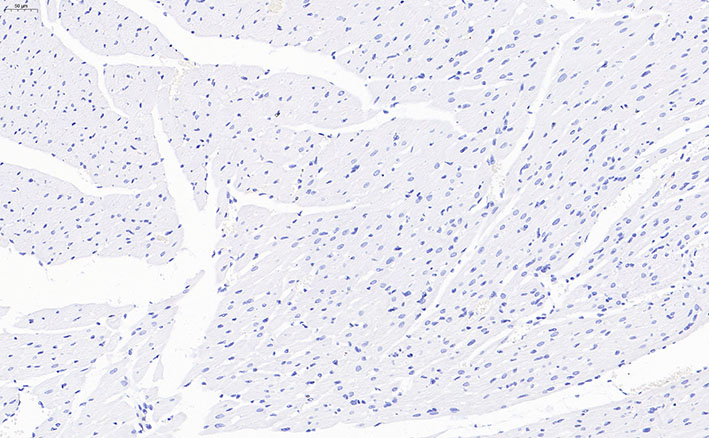

Supplement: Supplemental Information 3 [file peerj-11-16097-s003.zip › Raw data for IHC images/PAR2/Negative control/LAD/B.jpg]

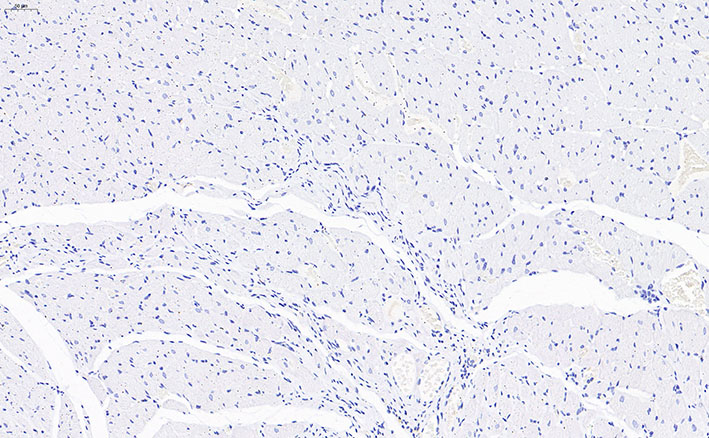

Supplement: Supplemental Information 3 [file peerj-11-16097-s003.zip › Raw data for IHC images/PAR2/Negative control/LAD/C.jpg]

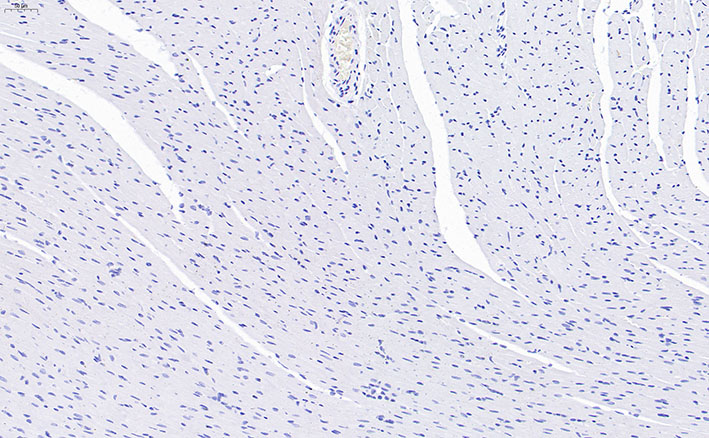

Supplement: Supplemental Information 3 [file peerj-11-16097-s003.zip › Raw data for IHC images/PAR2/Negative control/LAD+FSLLRY/A.jpg]

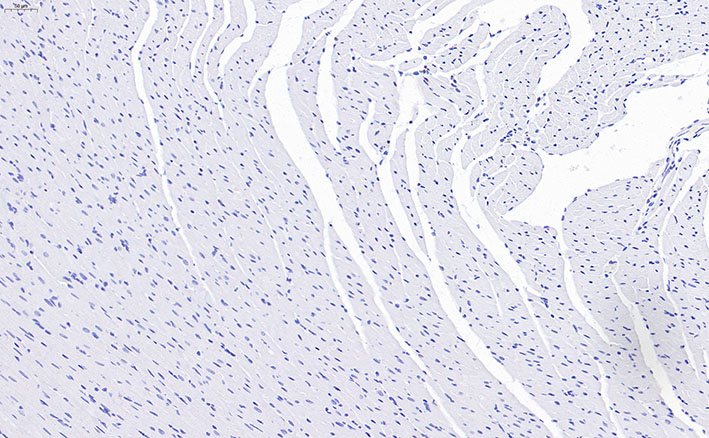

Supplement: Supplemental Information 3 [file peerj-11-16097-s003.zip › Raw data for IHC images/PAR2/Negative control/LAD+FSLLRY/B.jpg]

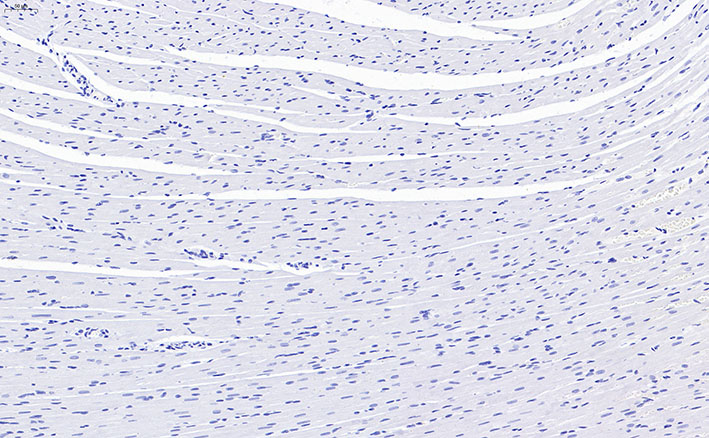

Supplement: Supplemental Information 3 [file peerj-11-16097-s003.zip › Raw data for IHC images/PAR2/Negative control/LAD+FSLLRY/C.jpg]

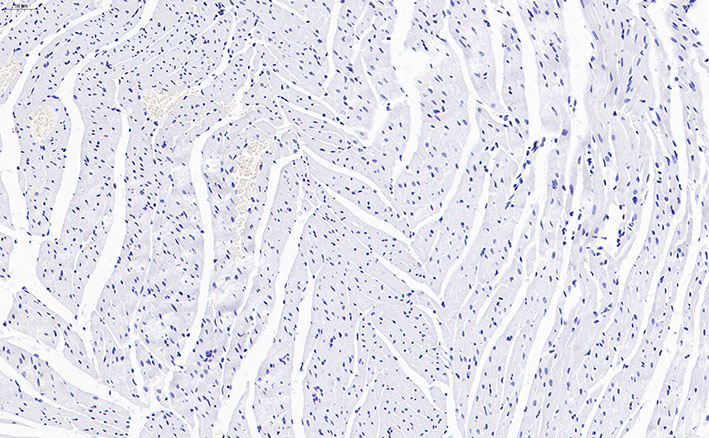

Supplement: Supplemental Information 3 [file peerj-11-16097-s003.zip › Raw data for IHC images/PAR2/Negative control/LAD+RIV/A.jpg]
