# Supplementary figures and images for: Rivaroxaban, a direct inhibitor of coagulation factor Xa, attenuates adverse cardiac remodeling in rats by regulating the PAR-2 and TGF-β1 signaling pathways (part 2 of 2)
Source: PeerJ. 2023 Sep 27;11:e16097. doi: 10.7717/peerj.16097 (PMC10541813; doi:10.7717/peerj.16097)

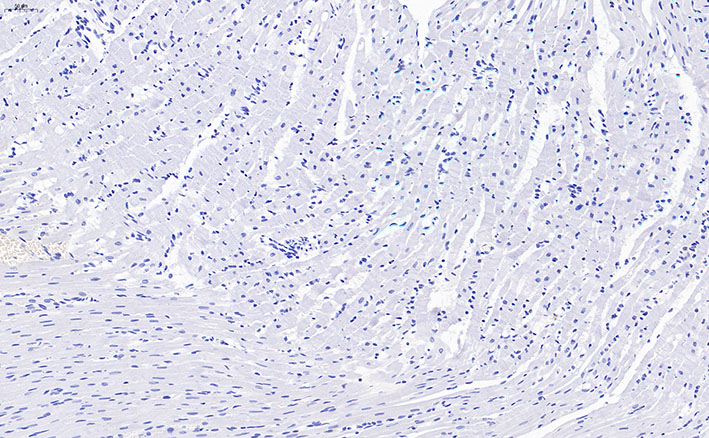

Supplement: Supplemental Information 3 [file peerj-11-16097-s003.zip › Raw data for IHC images/PAR2/Negative control/LAD+RIV/B.jpg]

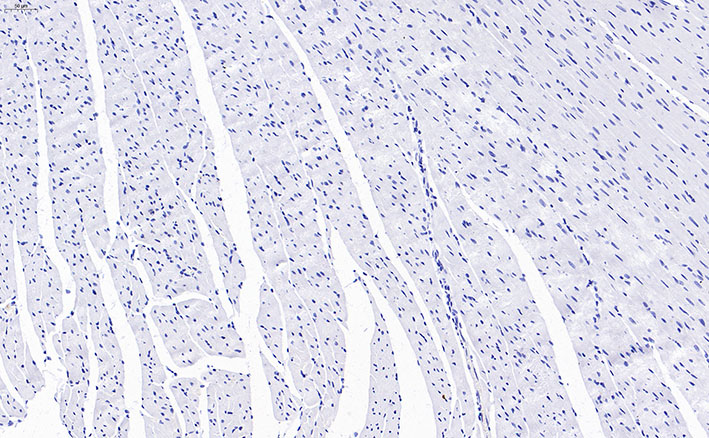

Supplement: Supplemental Information 3 [file peerj-11-16097-s003.zip › Raw data for IHC images/PAR2/Negative control/LAD+RIV/C.jpg]

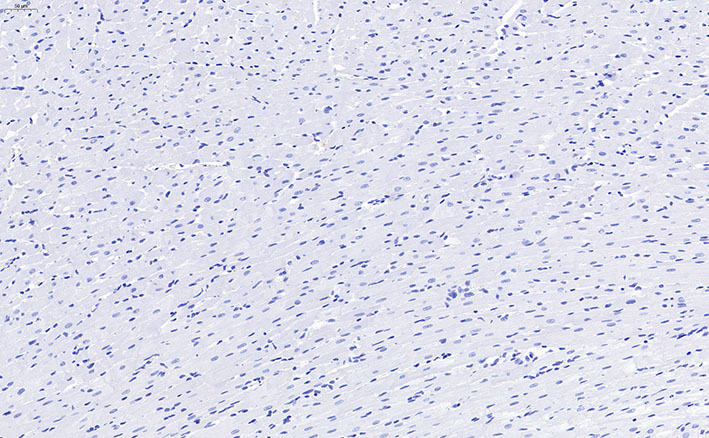

Supplement: Supplemental Information 3 [file peerj-11-16097-s003.zip › Raw data for IHC images/PAR2/Negative control/Sham/A.jpg]

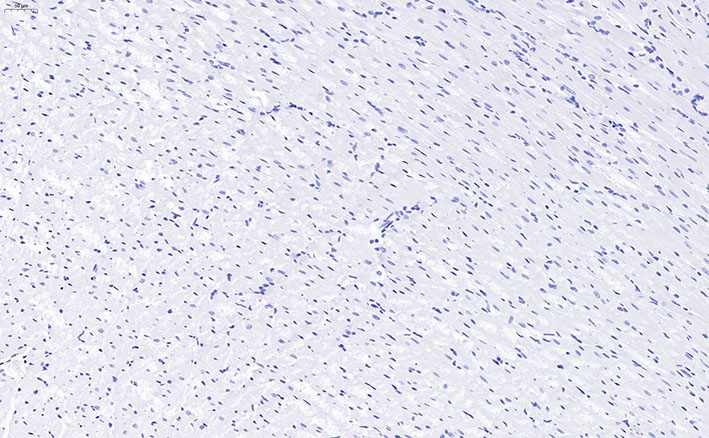

Supplement: Supplemental Information 3 [file peerj-11-16097-s003.zip › Raw data for IHC images/PAR2/Negative control/Sham/B.jpg]

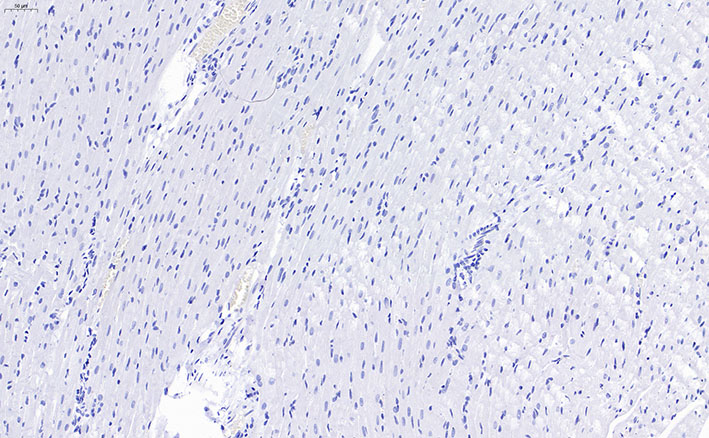

Supplement: Supplemental Information 3 [file peerj-11-16097-s003.zip › Raw data for IHC images/PAR2/Negative control/Sham/C.jpg]

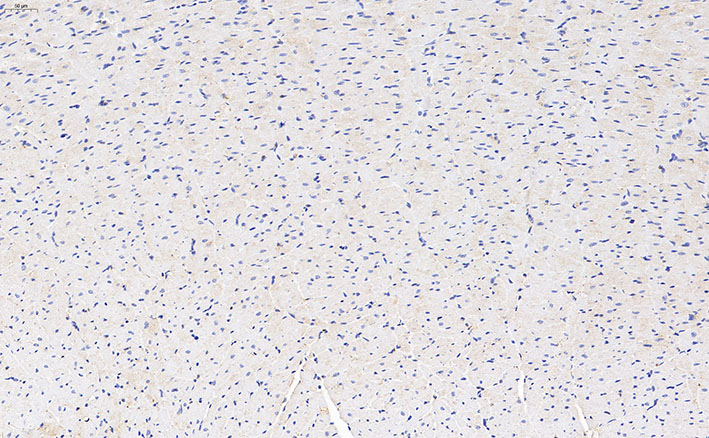

Supplement: Supplemental Information 3 [file peerj-11-16097-s003.zip › Raw data for IHC images/PAR2/Sham/A.jpg]

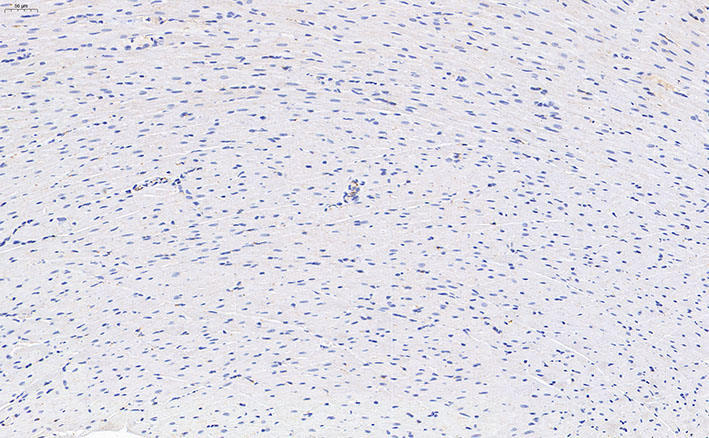

Supplement: Supplemental Information 3 [file peerj-11-16097-s003.zip › Raw data for IHC images/PAR2/Sham/B.jpg]

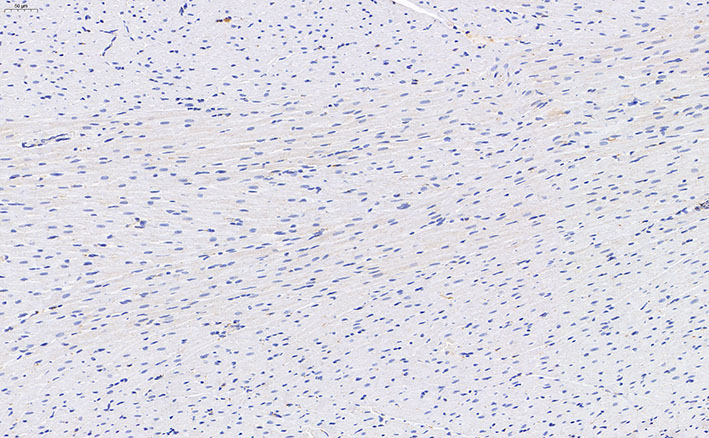

Supplement: Supplemental Information 3 [file peerj-11-16097-s003.zip › Raw data for IHC images/PAR2/Sham/C.jpg]

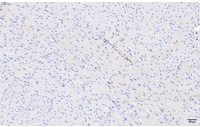

Supplement: Supplemental Information 3 [file peerj-11-16097-s003.zip › Raw data for IHC images/PAR2/Sham+FSLLRY/A.png]

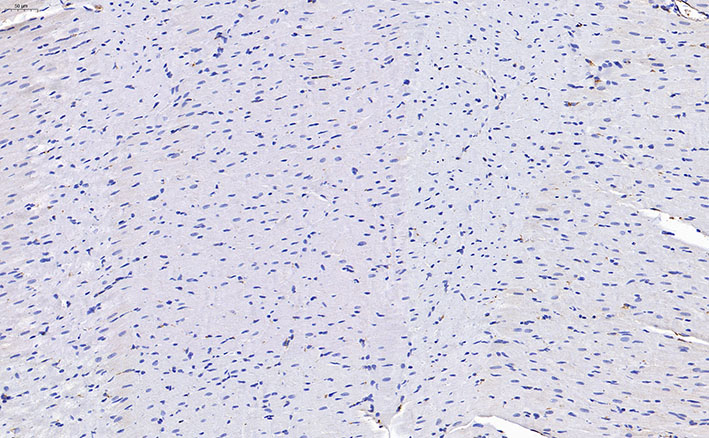

Supplement: Supplemental Information 3 [file peerj-11-16097-s003.zip › Raw data for IHC images/PAR2/Sham+FSLLRY/B.jpg]

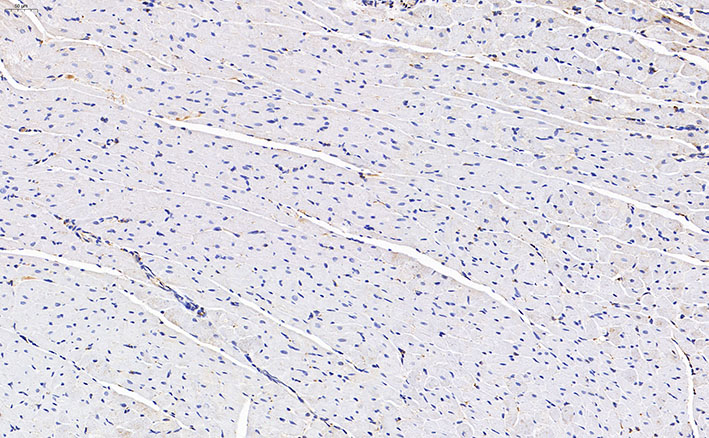

Supplement: Supplemental Information 3 [file peerj-11-16097-s003.zip › Raw data for IHC images/PAR2/Sham+FSLLRY/C.jpg]

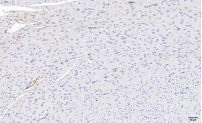

Supplement: Supplemental Information 3 [file peerj-11-16097-s003.zip › Raw data for IHC images/PAR2/Sham+RIV/A.png]

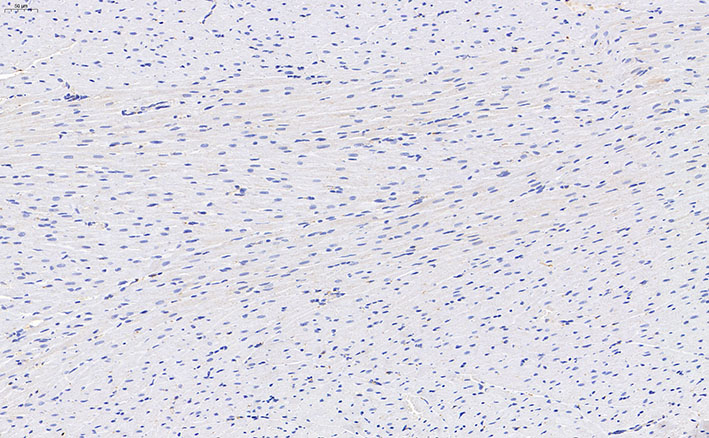

Supplement: Supplemental Information 3 [file peerj-11-16097-s003.zip › Raw data for IHC images/PAR2/Sham+RIV/B.jpg]

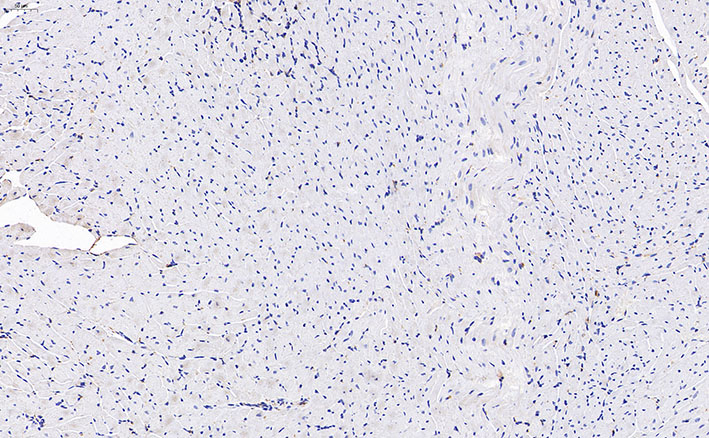

Supplement: Supplemental Information 3 [file peerj-11-16097-s003.zip › Raw data for IHC images/PAR2/Sham+RIV/C.jpg]

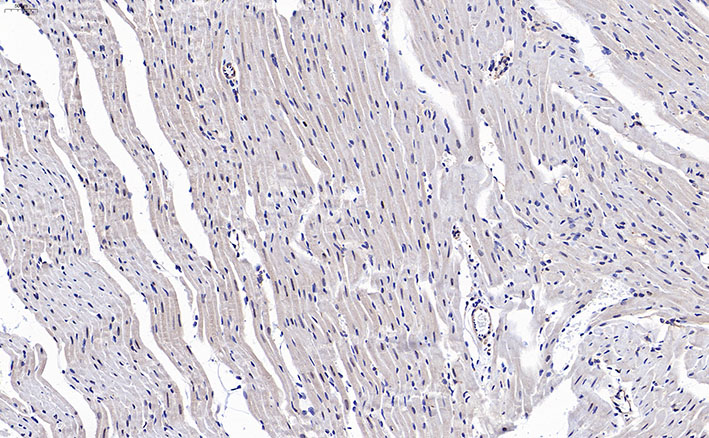

Supplement: Supplemental Information 3 [file peerj-11-16097-s003.zip › Raw data for IHC images/TGF-a┬/LAD/A.jpg]

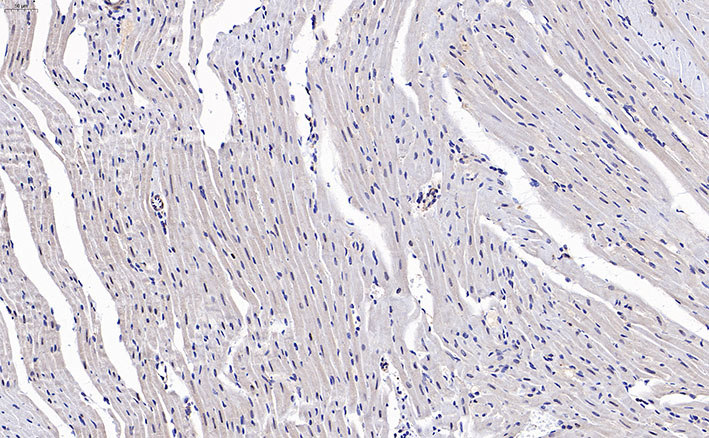

Supplement: Supplemental Information 3 [file peerj-11-16097-s003.zip › Raw data for IHC images/TGF-a┬/LAD/B.jpg]

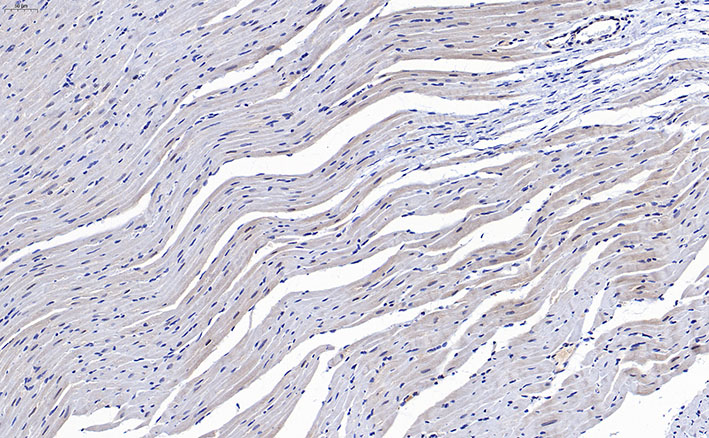

Supplement: Supplemental Information 3 [file peerj-11-16097-s003.zip › Raw data for IHC images/TGF-a┬/LAD/C.jpg]

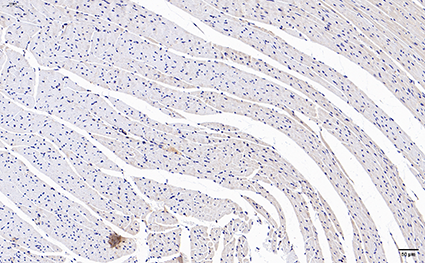

Supplement: Supplemental Information 3 [file peerj-11-16097-s003.zip › Raw data for IHC images/TGF-a┬/LAD+FSLLRY/A.tif]

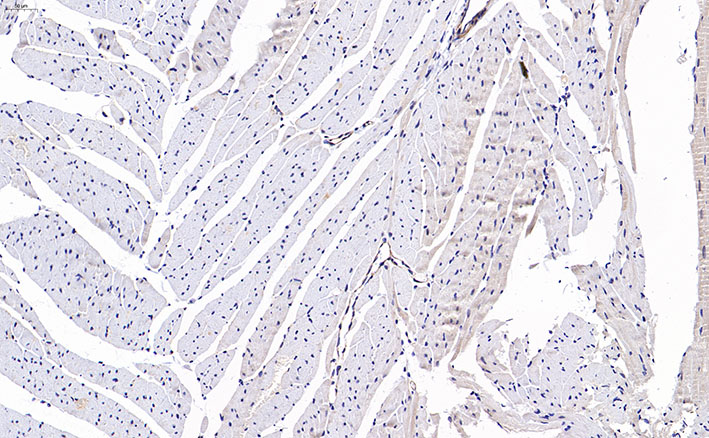

Supplement: Supplemental Information 3 [file peerj-11-16097-s003.zip › Raw data for IHC images/TGF-a┬/LAD+FSLLRY/B.jpg]

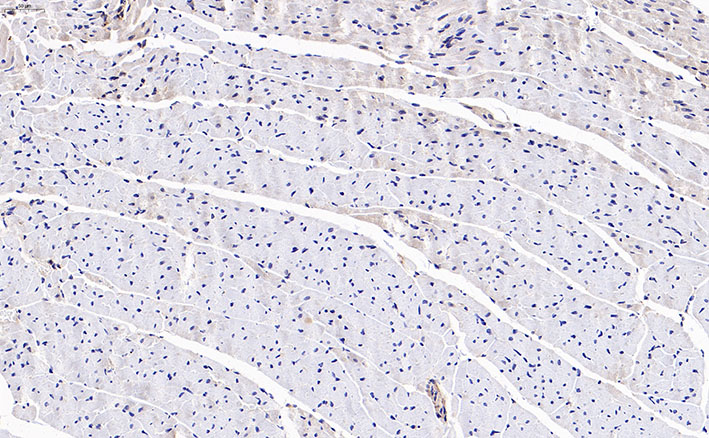

Supplement: Supplemental Information 3 [file peerj-11-16097-s003.zip › Raw data for IHC images/TGF-a┬/LAD+FSLLRY/C.jpg]

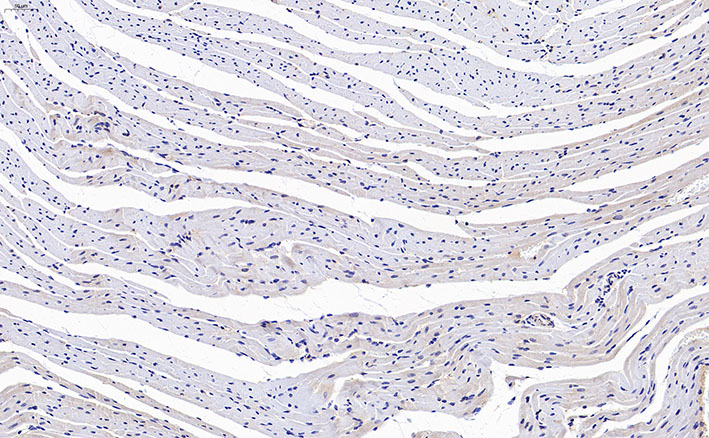

Supplement: Supplemental Information 3 [file peerj-11-16097-s003.zip › Raw data for IHC images/TGF-a┬/LAD+RIV/A.jpg]

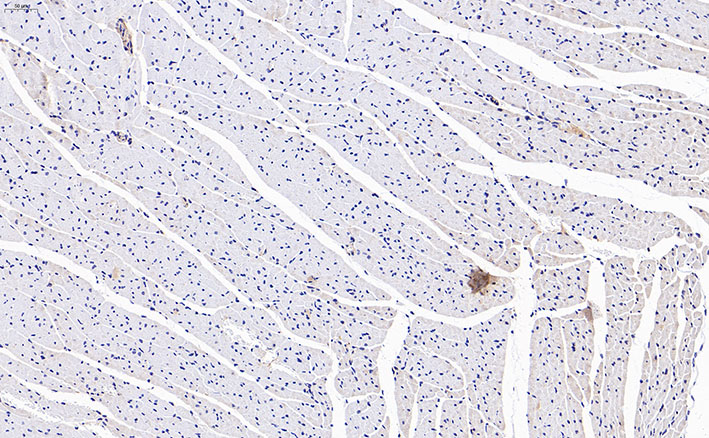

Supplement: Supplemental Information 3 [file peerj-11-16097-s003.zip › Raw data for IHC images/TGF-a┬/LAD+RIV/B.jpg]

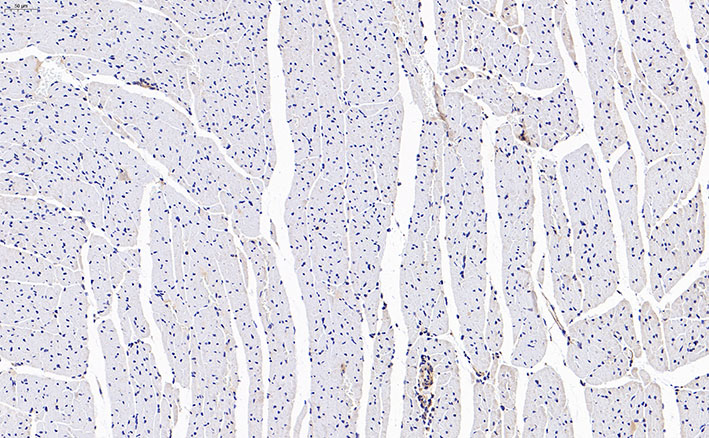

Supplement: Supplemental Information 3 [file peerj-11-16097-s003.zip › Raw data for IHC images/TGF-a┬/LAD+RIV/C.jpg]

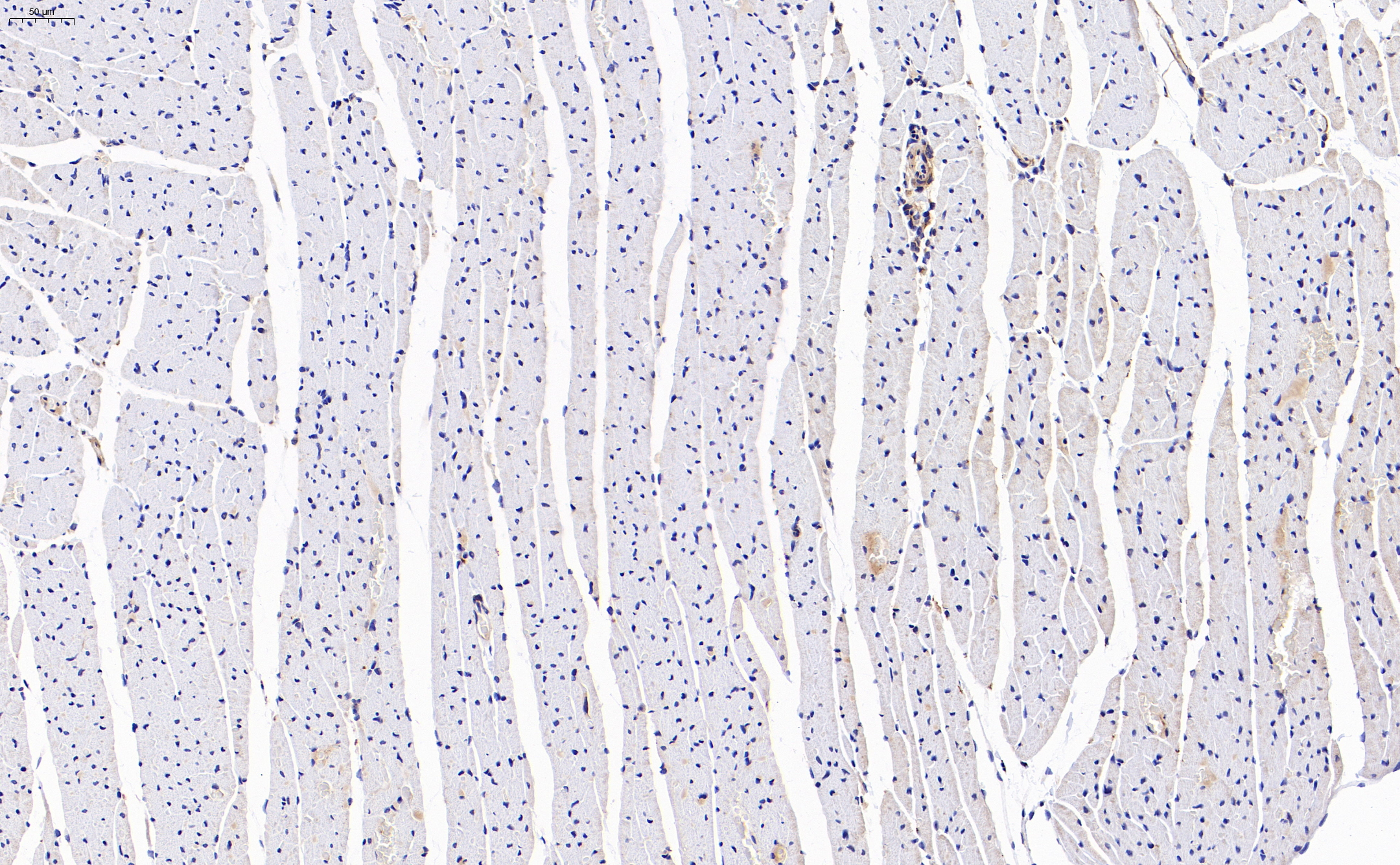

Supplement: Supplemental Information 3 [file peerj-11-16097-s003.zip › Raw data for IHC images/TGF-a┬/LAD+RIV/Riv A48ú¿╨─ú⌐ TGF-B_20.0x.jpg]

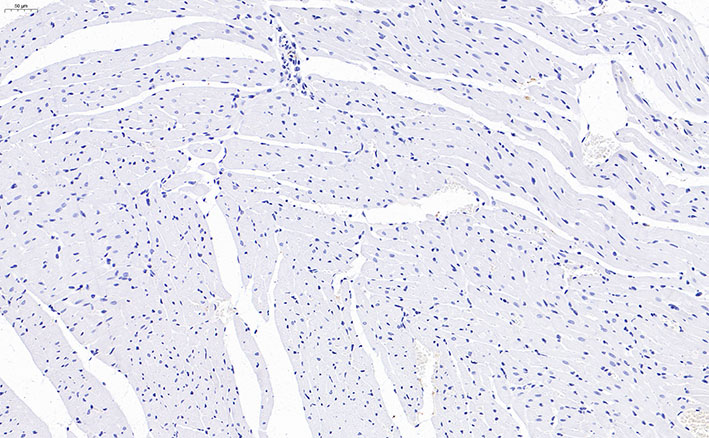

Supplement: Supplemental Information 3 [file peerj-11-16097-s003.zip › Raw data for IHC images/TGF-a┬/Negative control/LAD/A.jpg]

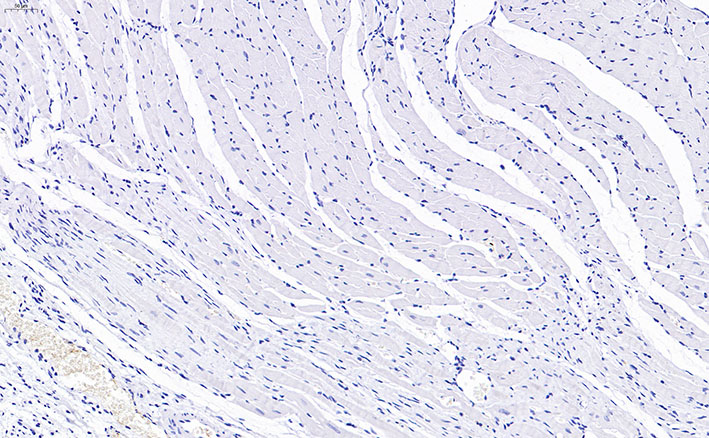

Supplement: Supplemental Information 3 [file peerj-11-16097-s003.zip › Raw data for IHC images/TGF-a┬/Negative control/LAD/B.jpg]

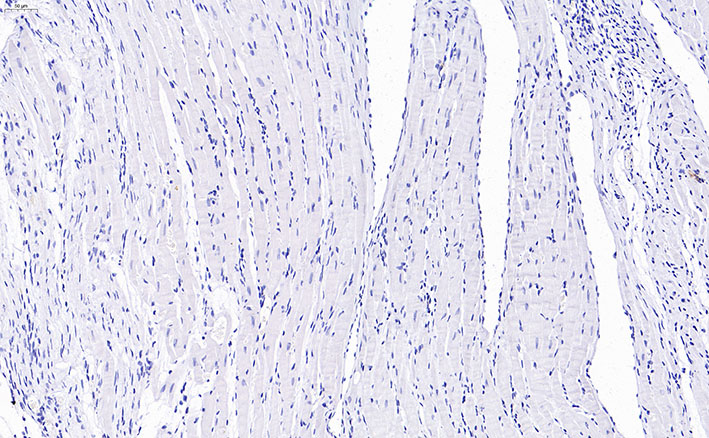

Supplement: Supplemental Information 3 [file peerj-11-16097-s003.zip › Raw data for IHC images/TGF-a┬/Negative control/LAD/C.jpg]

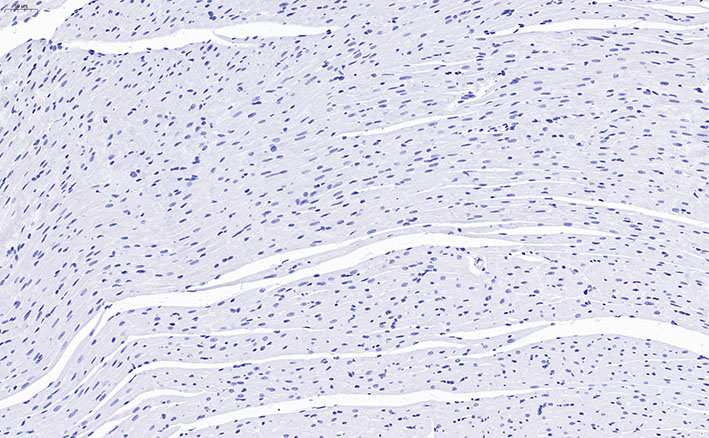

Supplement: Supplemental Information 3 [file peerj-11-16097-s003.zip › Raw data for IHC images/TGF-a┬/Negative control/LAD+FSLLRY/A.jpg]

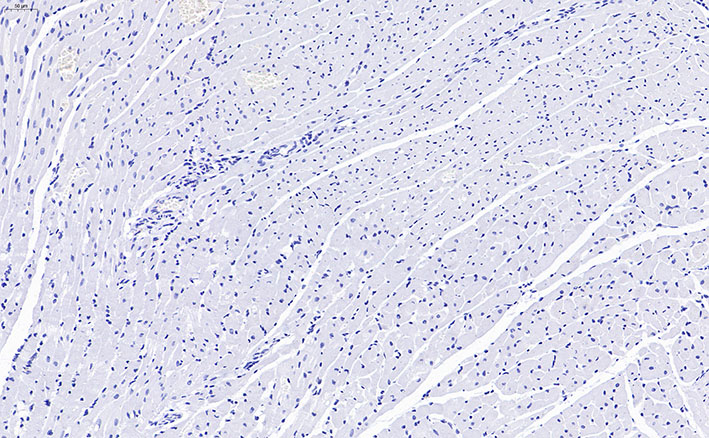

Supplement: Supplemental Information 3 [file peerj-11-16097-s003.zip › Raw data for IHC images/TGF-a┬/Negative control/LAD+FSLLRY/B.jpg]

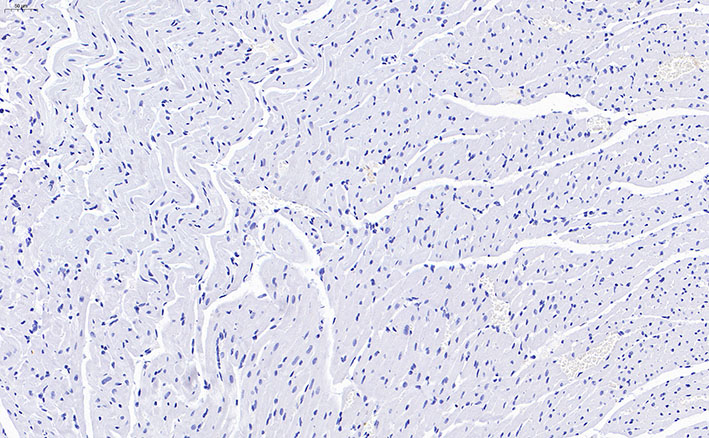

Supplement: Supplemental Information 3 [file peerj-11-16097-s003.zip › Raw data for IHC images/TGF-a┬/Negative control/LAD+FSLLRY/C.jpg]

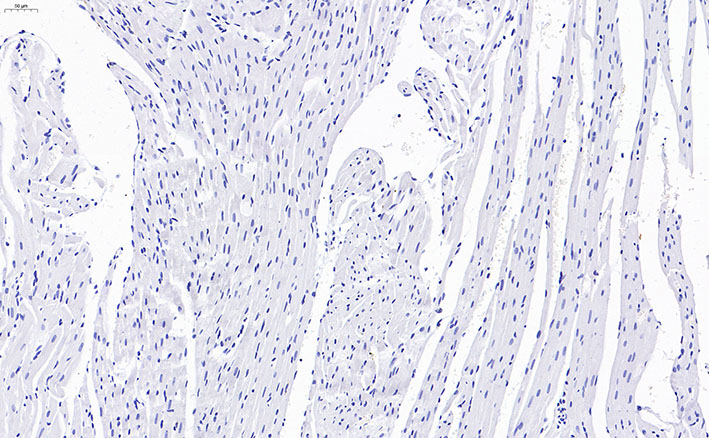

Supplement: Supplemental Information 3 [file peerj-11-16097-s003.zip › Raw data for IHC images/TGF-a┬/Negative control/LAD+RIV/A.jpg]

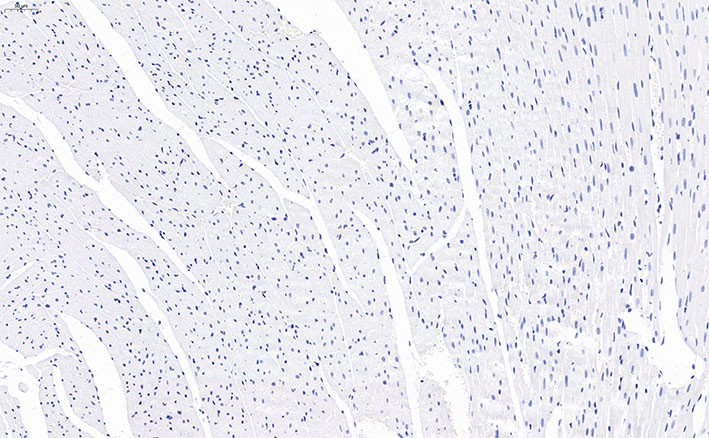

Supplement: Supplemental Information 3 [file peerj-11-16097-s003.zip › Raw data for IHC images/TGF-a┬/Negative control/LAD+RIV/B.jpg]

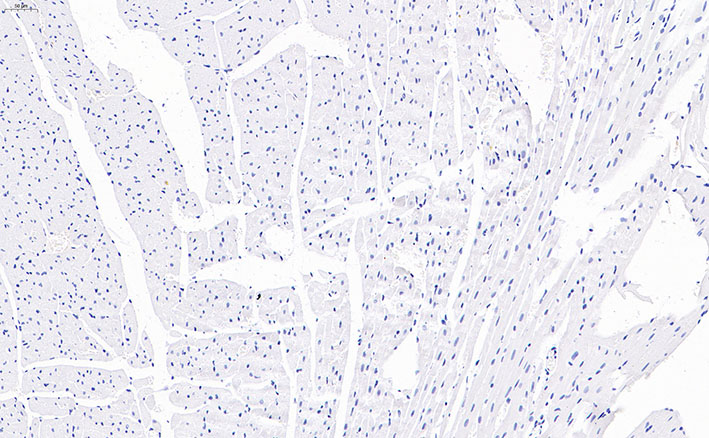

Supplement: Supplemental Information 3 [file peerj-11-16097-s003.zip › Raw data for IHC images/TGF-a┬/Negative control/LAD+RIV/C.jpg]

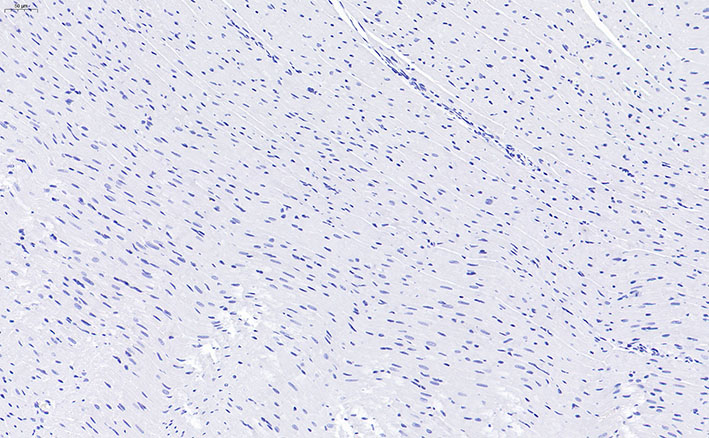

Supplement: Supplemental Information 3 [file peerj-11-16097-s003.zip › Raw data for IHC images/TGF-a┬/Negative control/Sham/A.jpg]

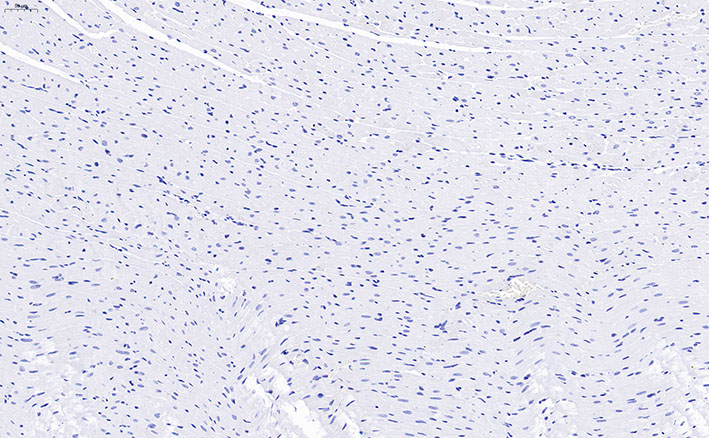

Supplement: Supplemental Information 3 [file peerj-11-16097-s003.zip › Raw data for IHC images/TGF-a┬/Negative control/Sham/B.jpg]

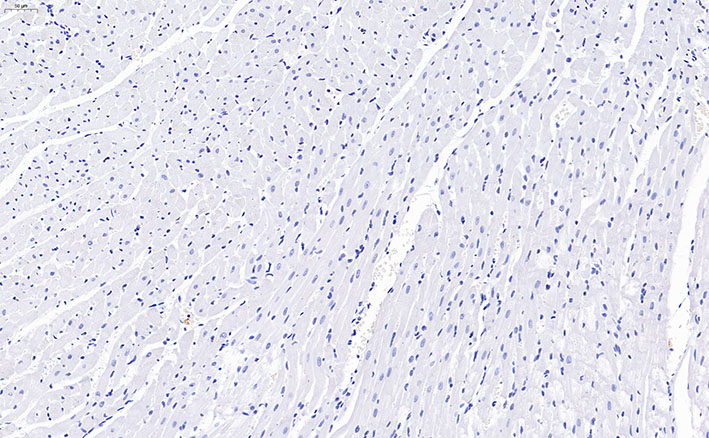

Supplement: Supplemental Information 3 [file peerj-11-16097-s003.zip › Raw data for IHC images/TGF-a┬/Negative control/Sham/C.jpg]

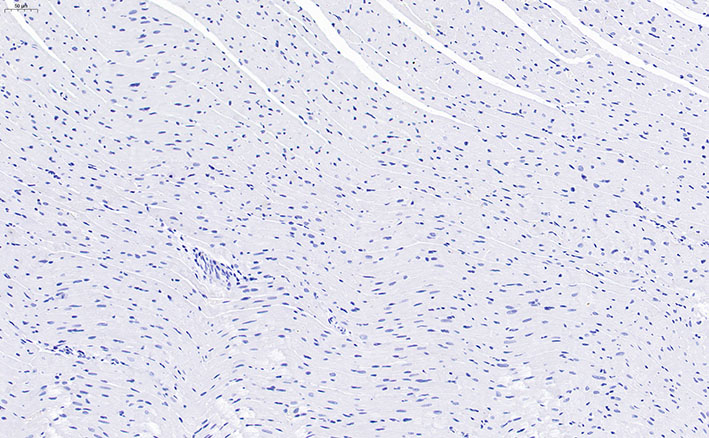

Supplement: Supplemental Information 3 [file peerj-11-16097-s003.zip › Raw data for IHC images/TGF-a┬/Sham/A.jpg]

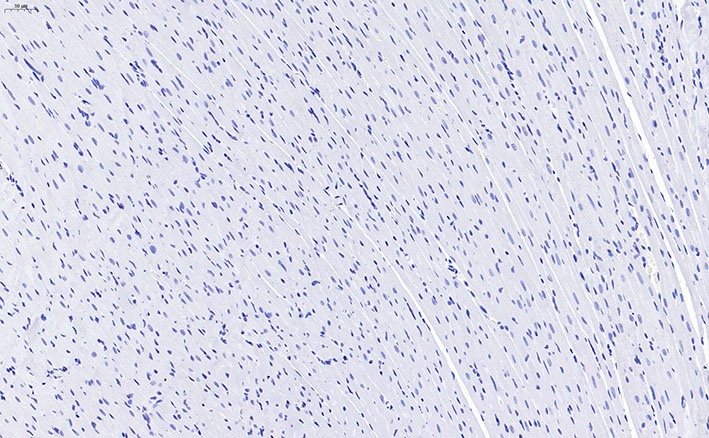

Supplement: Supplemental Information 3 [file peerj-11-16097-s003.zip › Raw data for IHC images/TGF-a┬/Sham/B.jpg]

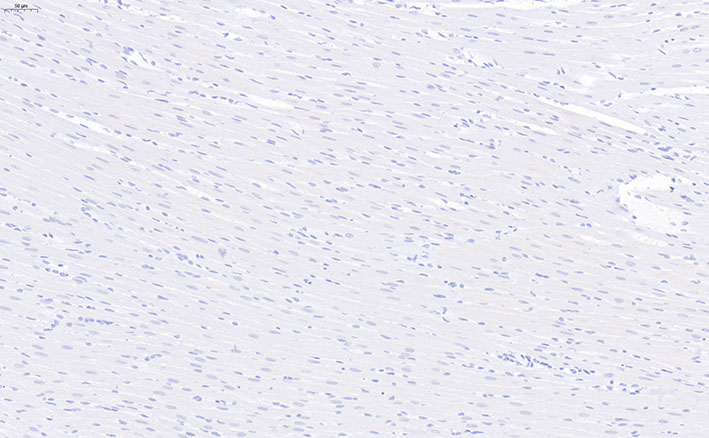

Supplement: Supplemental Information 3 [file peerj-11-16097-s003.zip › Raw data for IHC images/TGF-a┬/Sham/C.jpg]

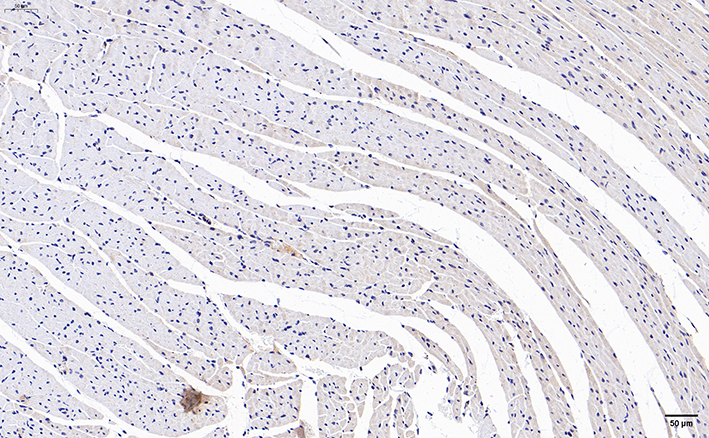

Supplement: Supplemental Information 3 [file peerj-11-16097-s003.zip › Raw data for IHC images/TGF-a┬/Sham+FSLLRY/A.tif]

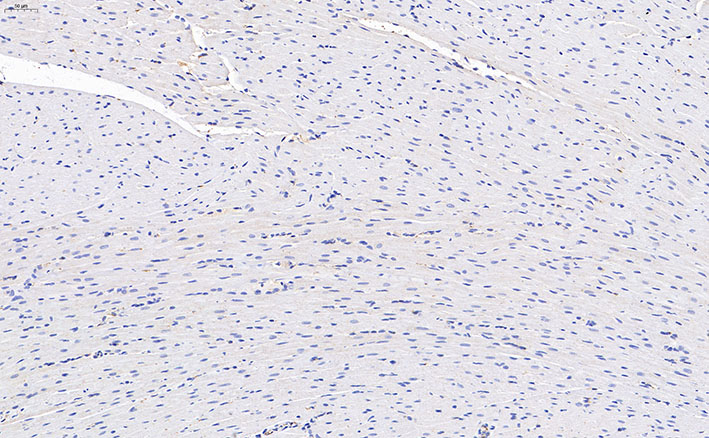

Supplement: Supplemental Information 3 [file peerj-11-16097-s003.zip › Raw data for IHC images/TGF-a┬/Sham+FSLLRY/B.jpg]

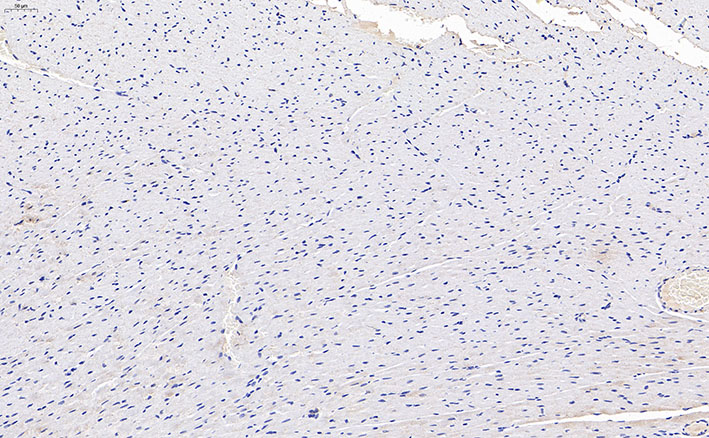

Supplement: Supplemental Information 3 [file peerj-11-16097-s003.zip › Raw data for IHC images/TGF-a┬/Sham+FSLLRY/C.jpg]

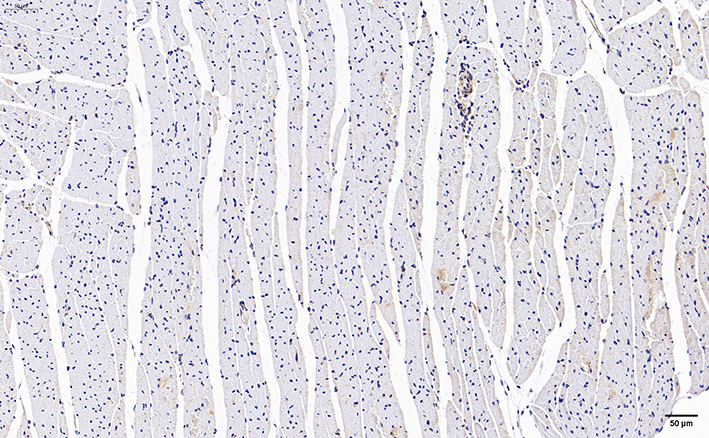

Supplement: Supplemental Information 3 [file peerj-11-16097-s003.zip › Raw data for IHC images/TGF-a┬/Sham+RIV/A.tif]

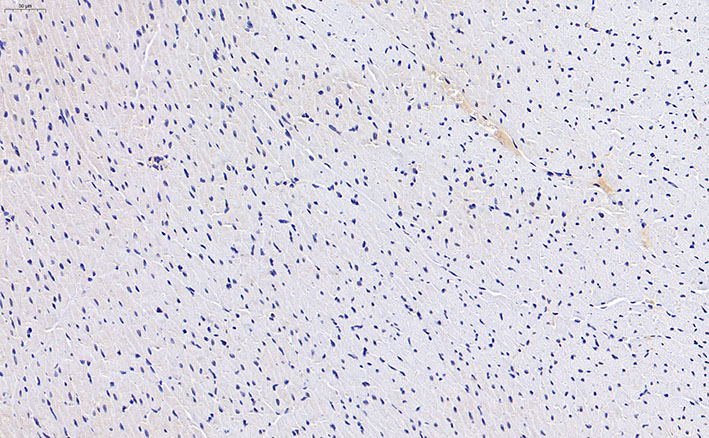

Supplement: Supplemental Information 3 [file peerj-11-16097-s003.zip › Raw data for IHC images/TGF-a┬/Sham+RIV/B.jpg]

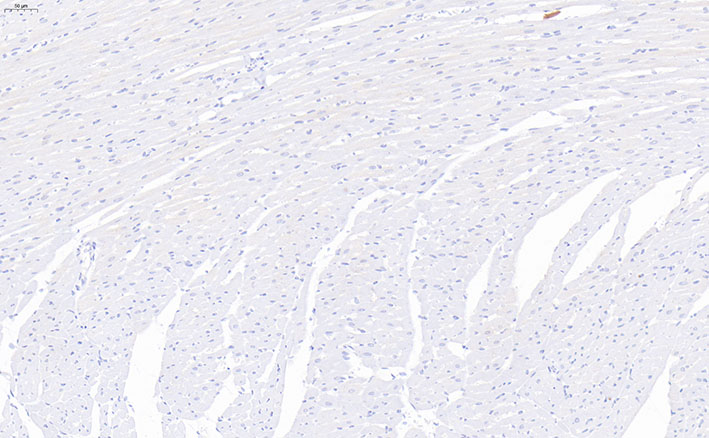

Supplement: Supplemental Information 3 [file peerj-11-16097-s003.zip › Raw data for IHC images/TGF-a┬/Sham+RIV/C.jpg]

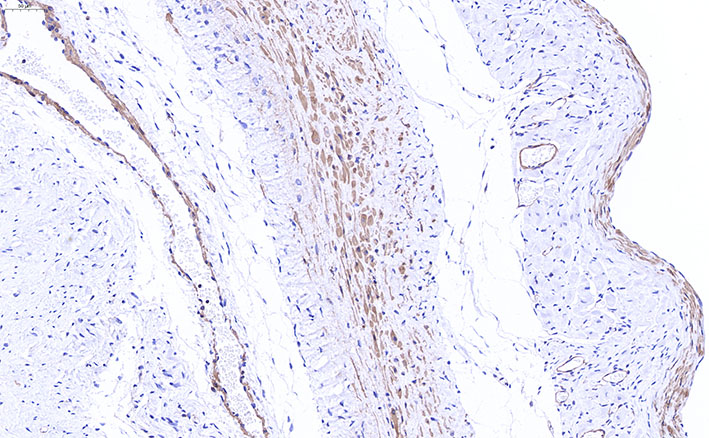

Supplement: Supplemental Information 3 [file peerj-11-16097-s003.zip › Raw data for IHC images/a┴-SMA/LAD/A.jpg]

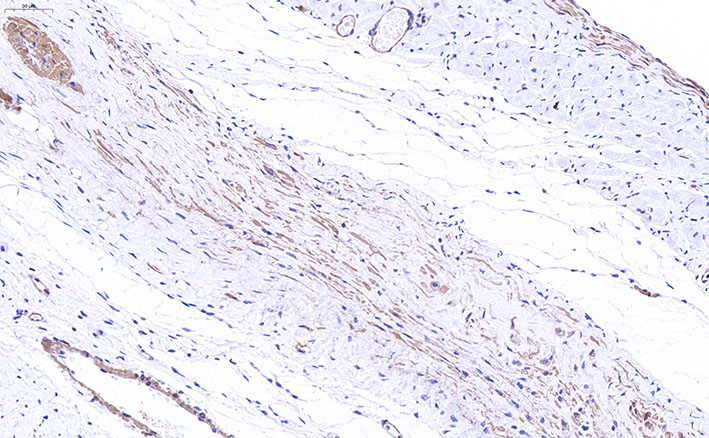

Supplement: Supplemental Information 3 [file peerj-11-16097-s003.zip › Raw data for IHC images/a┴-SMA/LAD/B.jpg]

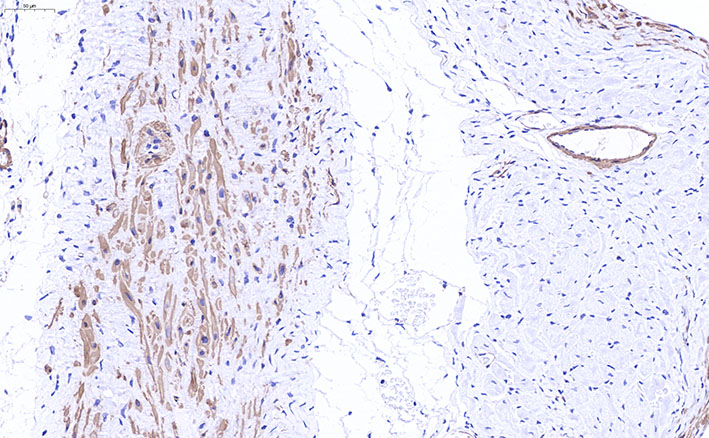

Supplement: Supplemental Information 3 [file peerj-11-16097-s003.zip › Raw data for IHC images/a┴-SMA/LAD/C.jpg]

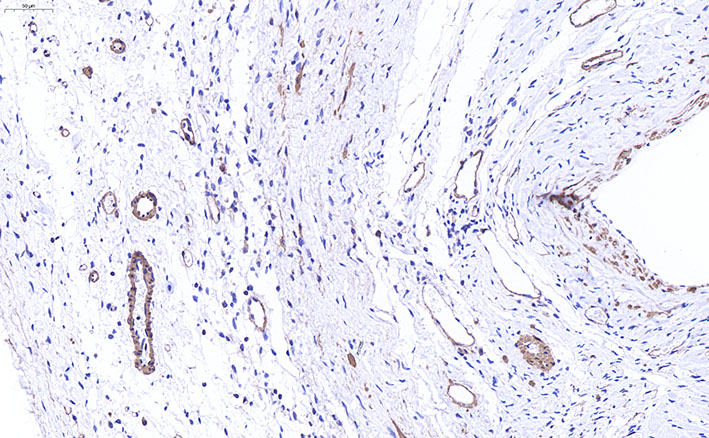

Supplement: Supplemental Information 3 [file peerj-11-16097-s003.zip › Raw data for IHC images/a┴-SMA/LAD+FSLLRY/A.jpg]

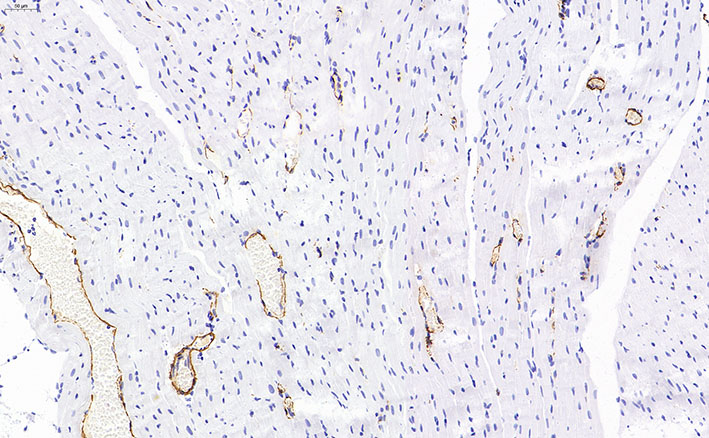

Supplement: Supplemental Information 3 [file peerj-11-16097-s003.zip › Raw data for IHC images/a┴-SMA/LAD+FSLLRY/B.jpg]

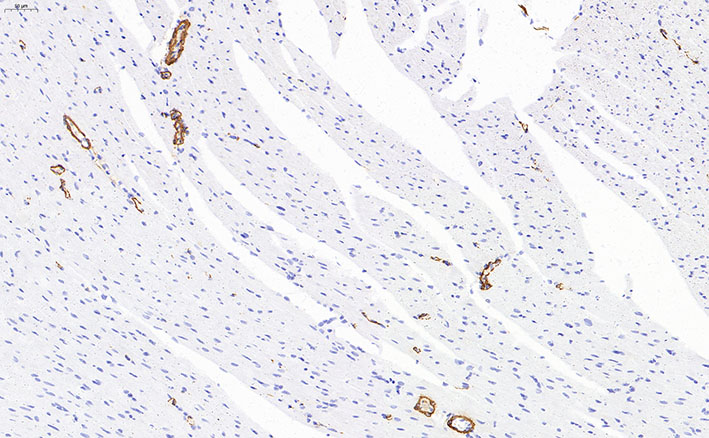

Supplement: Supplemental Information 3 [file peerj-11-16097-s003.zip › Raw data for IHC images/a┴-SMA/LAD+FSLLRY/C.jpg]

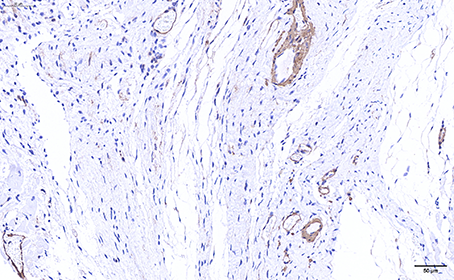

Supplement: Supplemental Information 3 [file peerj-11-16097-s003.zip › Raw data for IHC images/a┴-SMA/LAD+RIV/A.tif]

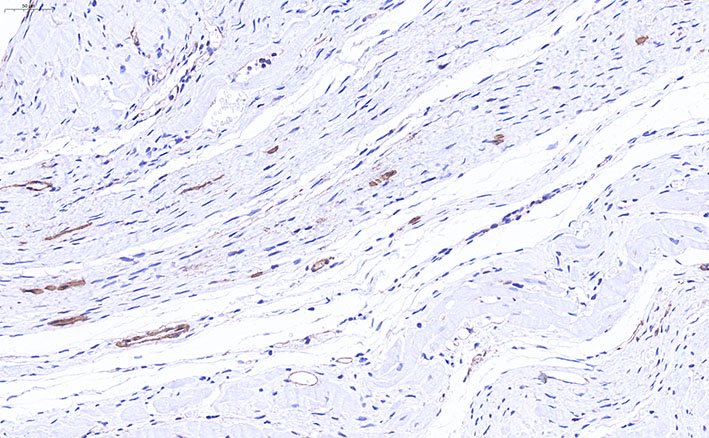

Supplement: Supplemental Information 3 [file peerj-11-16097-s003.zip › Raw data for IHC images/a┴-SMA/LAD+RIV/B.jpg]

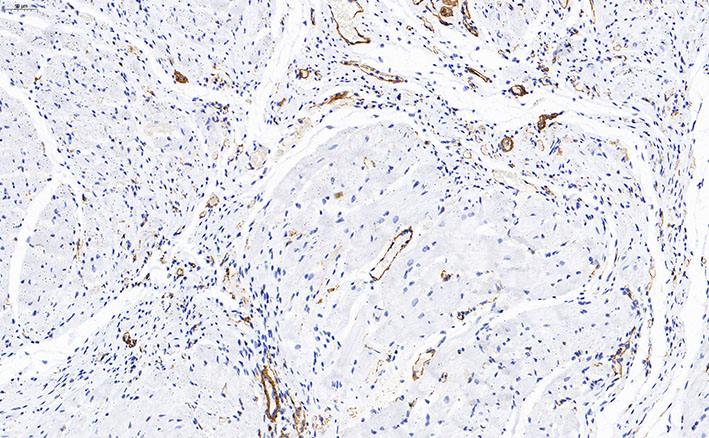

Supplement: Supplemental Information 3 [file peerj-11-16097-s003.zip › Raw data for IHC images/a┴-SMA/LAD+RIV/C.jpg]

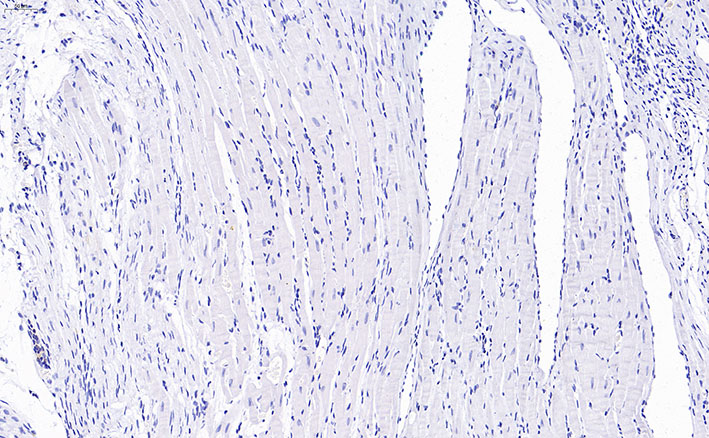

Supplement: Supplemental Information 3 [file peerj-11-16097-s003.zip › Raw data for IHC images/a┴-SMA/Negative control/LAD/A.jpg]

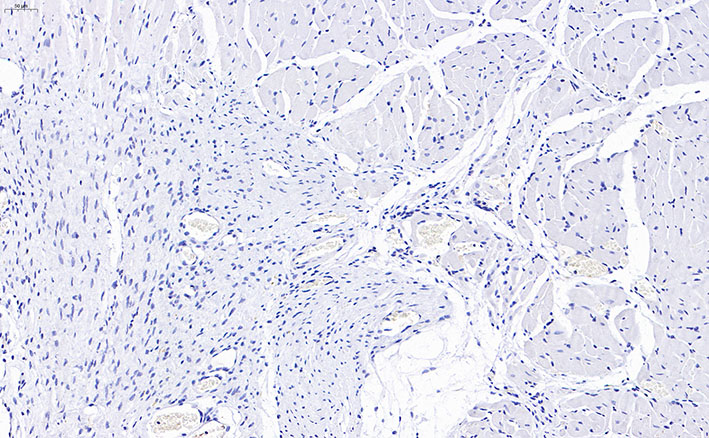

Supplement: Supplemental Information 3 [file peerj-11-16097-s003.zip › Raw data for IHC images/a┴-SMA/Negative control/LAD/B.jpg]

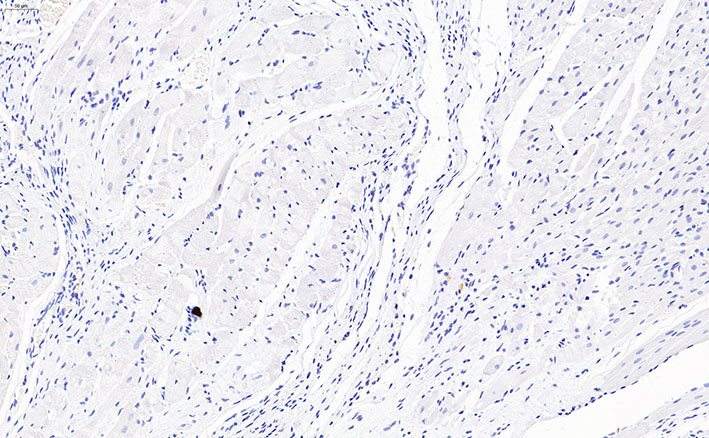

Supplement: Supplemental Information 3 [file peerj-11-16097-s003.zip › Raw data for IHC images/a┴-SMA/Negative control/LAD/C.jpg]

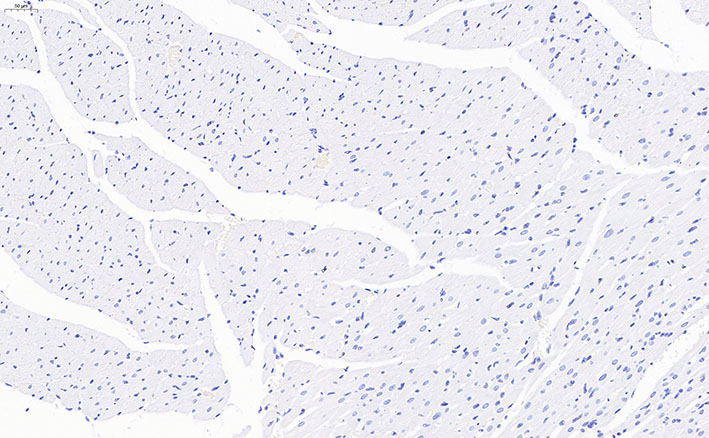

Supplement: Supplemental Information 3 [file peerj-11-16097-s003.zip › Raw data for IHC images/a┴-SMA/Negative control/LAD+FSLLRY/A.jpg]

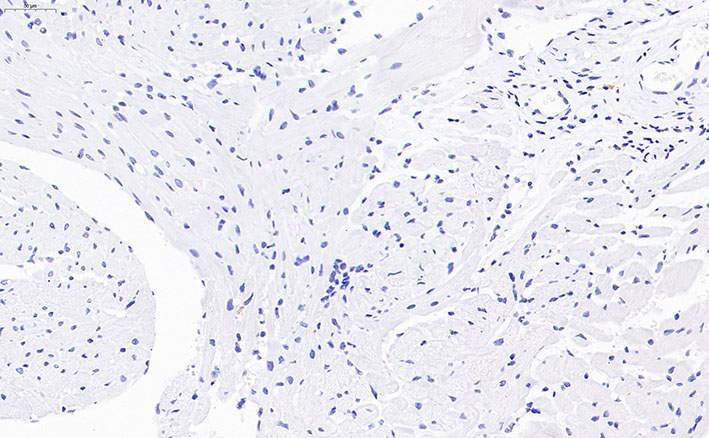

Supplement: Supplemental Information 3 [file peerj-11-16097-s003.zip › Raw data for IHC images/a┴-SMA/Negative control/LAD+FSLLRY/B.jpg]

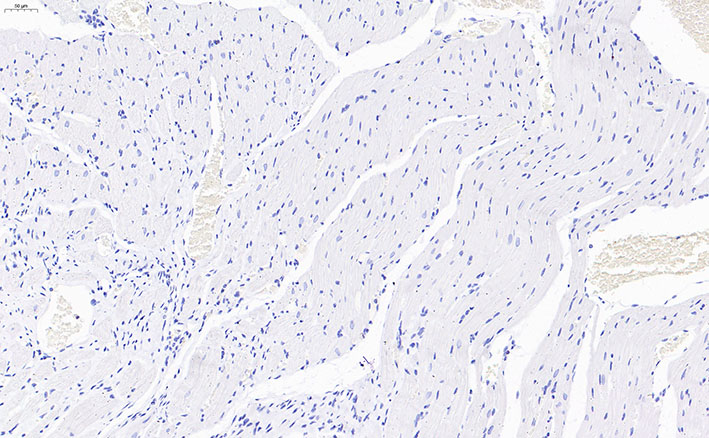

Supplement: Supplemental Information 3 [file peerj-11-16097-s003.zip › Raw data for IHC images/a┴-SMA/Negative control/LAD+FSLLRY/C.jpg]

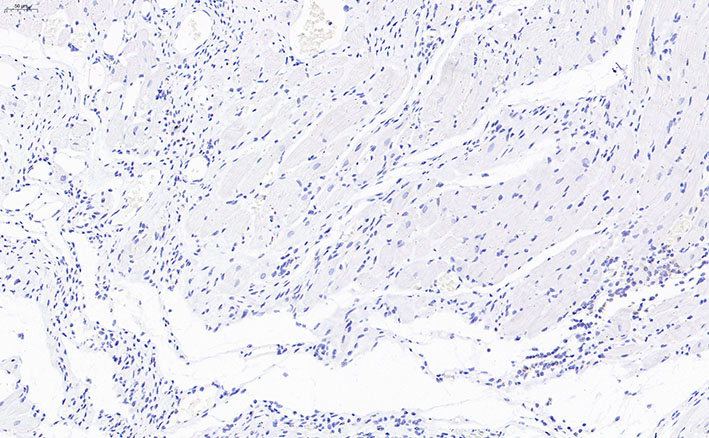

Supplement: Supplemental Information 3 [file peerj-11-16097-s003.zip › Raw data for IHC images/a┴-SMA/Negative control/LAD+RIV/A.jpg]

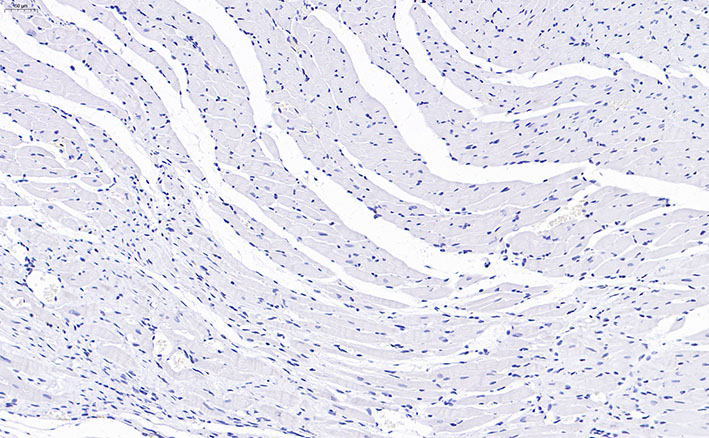

Supplement: Supplemental Information 3 [file peerj-11-16097-s003.zip › Raw data for IHC images/a┴-SMA/Negative control/LAD+RIV/B.jpg]

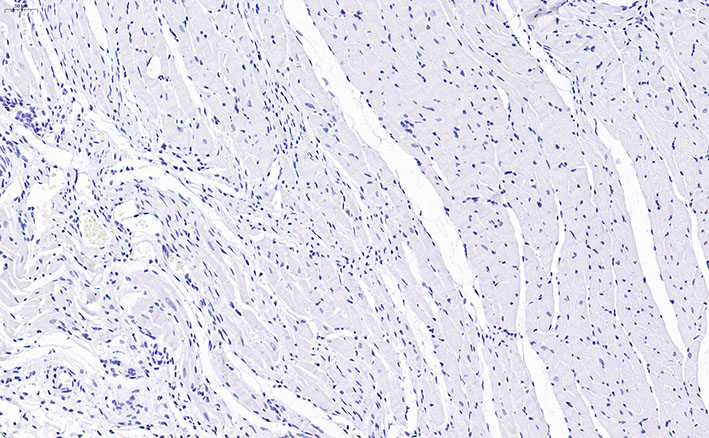

Supplement: Supplemental Information 3 [file peerj-11-16097-s003.zip › Raw data for IHC images/a┴-SMA/Negative control/LAD+RIV/C.jpg]

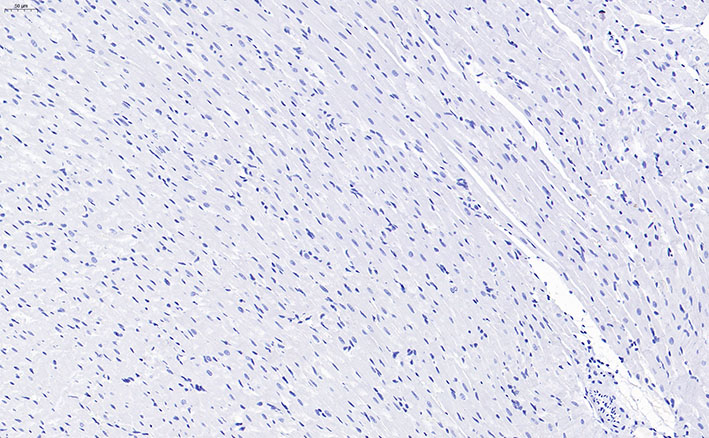

Supplement: Supplemental Information 3 [file peerj-11-16097-s003.zip › Raw data for IHC images/a┴-SMA/Negative control/Sham/A.jpg]

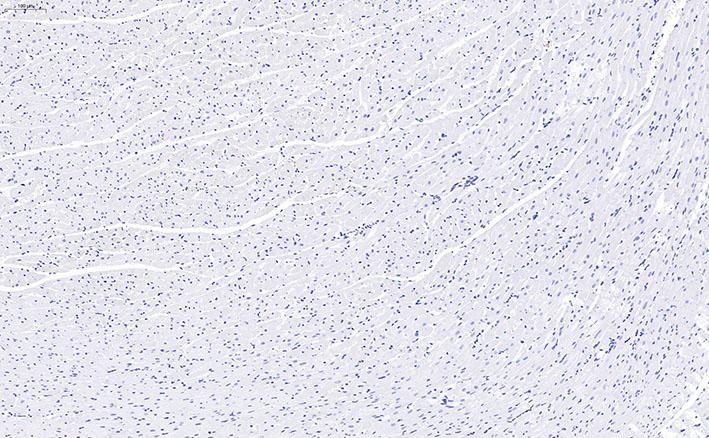

Supplement: Supplemental Information 3 [file peerj-11-16097-s003.zip › Raw data for IHC images/a┴-SMA/Negative control/Sham/B.jpg]

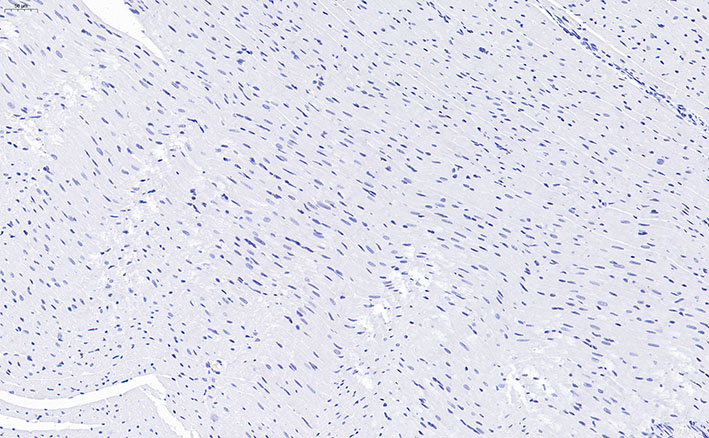

Supplement: Supplemental Information 3 [file peerj-11-16097-s003.zip › Raw data for IHC images/a┴-SMA/Negative control/Sham/C.jpg]

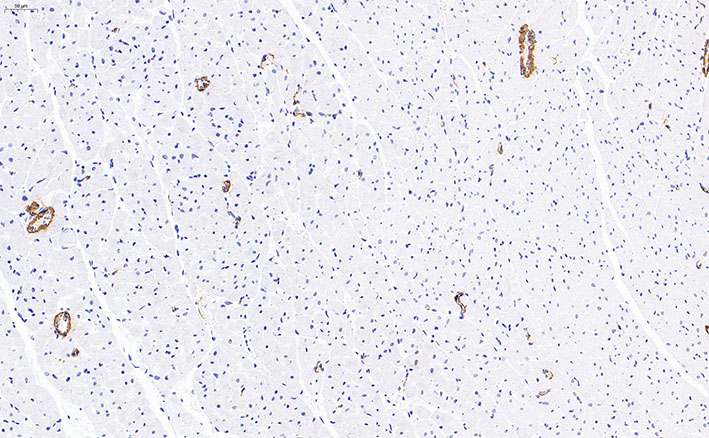

Supplement: Supplemental Information 3 [file peerj-11-16097-s003.zip › Raw data for IHC images/a┴-SMA/Sham/A.jpg]

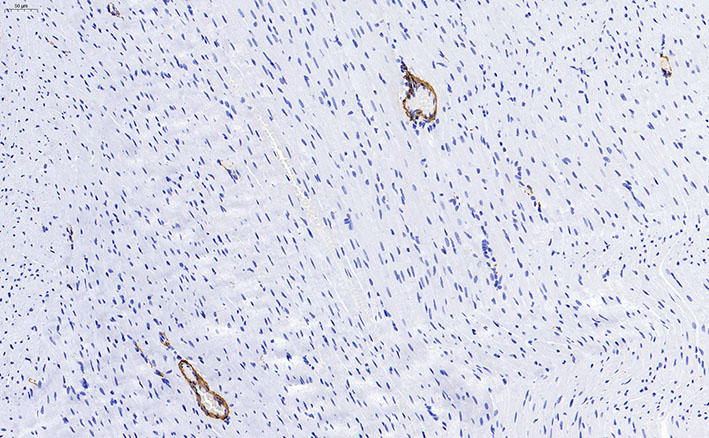

Supplement: Supplemental Information 3 [file peerj-11-16097-s003.zip › Raw data for IHC images/a┴-SMA/Sham/B.jpg]

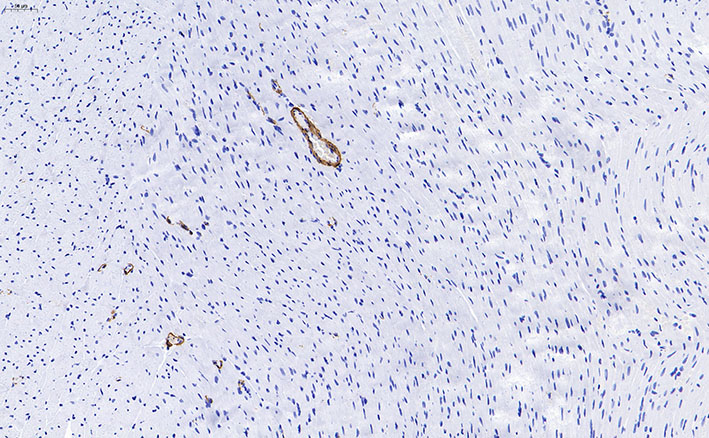

Supplement: Supplemental Information 3 [file peerj-11-16097-s003.zip › Raw data for IHC images/a┴-SMA/Sham/C.jpg]

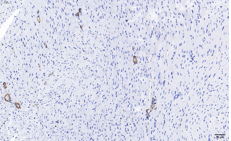

Supplement: Supplemental Information 3 [file peerj-11-16097-s003.zip › Raw data for IHC images/a┴-SMA/Sham+FSLLRY/A.png]

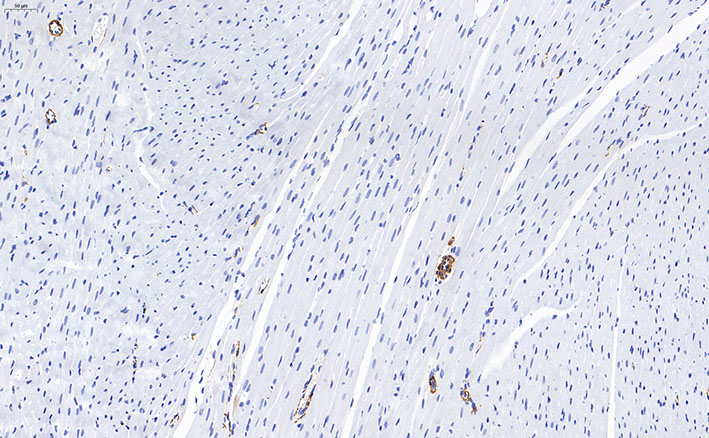

Supplement: Supplemental Information 3 [file peerj-11-16097-s003.zip › Raw data for IHC images/a┴-SMA/Sham+FSLLRY/B.jpg]

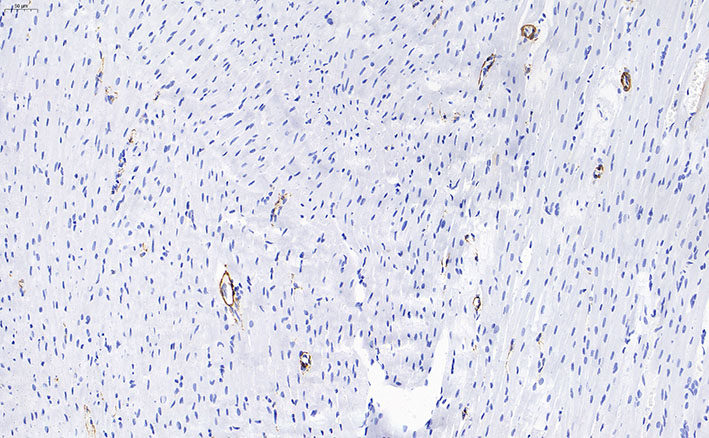

Supplement: Supplemental Information 3 [file peerj-11-16097-s003.zip › Raw data for IHC images/a┴-SMA/Sham+FSLLRY/C.jpg]

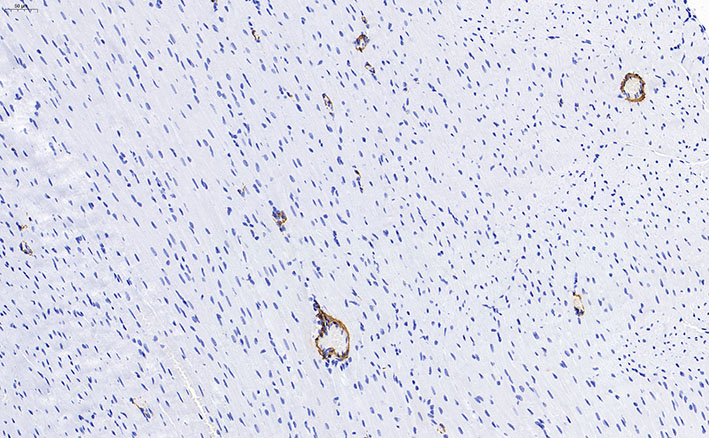

Supplement: Supplemental Information 3 [file peerj-11-16097-s003.zip › Raw data for IHC images/a┴-SMA/Sham+RIV/A.jpg]

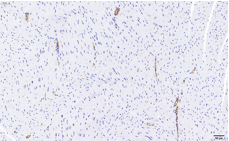

Supplement: Supplemental Information 3 [file peerj-11-16097-s003.zip › Raw data for IHC images/a┴-SMA/Sham+RIV/B.png]

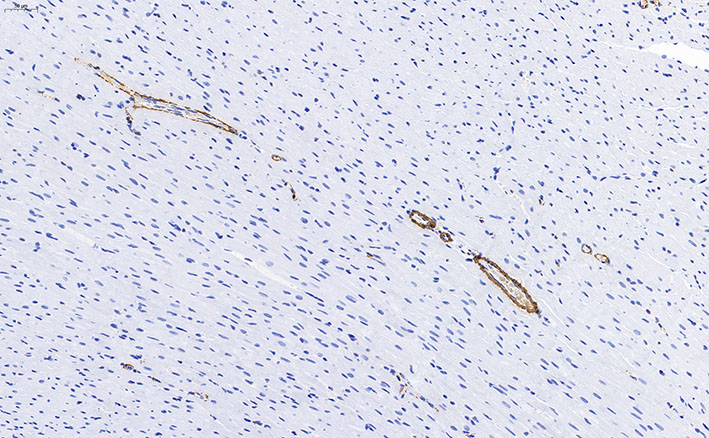

Supplement: Supplemental Information 3 [file peerj-11-16097-s003.zip › Raw data for IHC images/a┴-SMA/Sham+RIV/C.jpg]
